# A national internet-linked based database for pediatric interstitial lung diseases: the French network

**DOI:** 10.1186/1750-1172-7-40

**Published:** 2012-06-15

**Authors:** Nadia Nathan, Rola Abou Taam, Ralph Epaud, Christophe Delacourt, Antoine Deschildre, Philippe Reix, Raphaël Chiron, Ulrika de Pontbriand, Jacques Brouard, Michaël Fayon, Jean-Christophe Dubus, Lisa Giovannini-Chami, François Bremont, Katia Bessaci, Cyril Schweitzer, Marie-Laure Dalphin, Christophe Marguet, Véronique Houdouin, Françoise Troussier, Anne Sardet, Eglantine Hullo, Isabelle Gibertini, Malika Mahloul, Delphine Michon, Adrien Priouzeau, Laurie Galeron, Jean-François Vibert, Guillaume Thouvenin, Harriet Corvol, Jacques deBlic, Annick Clement, French RespiRare® Group

**Affiliations:** 1 AP-HP, Hôpital Trousseau, Pediatric Pulmonary Department, Paris, F-75012, France; 2 Université Pierre et Marie Curie-Paris6, Inserm, UMR S-U938, Paris, F-75012, France; 3 Pediatric Pulmonary Department, AP-HP, Hôpital Necker Enfants Malades, Paris, F-75015, France; 4 Université Paris Descartes-Paris5, Paris, F-75005, France; 5 Centre Hospitalier Intercommunal de Créteil, Pediatric Department, Inserm U955, Université Paris Est, Créteil, France; 6 Pediatric Department, Centre Hospitalier Universitaire de Lille, Lille, France; 7 Pediatric Pulmonary Department, Centre Hospitalier Universitaire de Lyon, Lyon, France; 8 Pediatric Pulmonary Department, Centre Hospitalier Universitaire de Montpellier, Montpellier, France; 9 Pediatric Pulmonary Department, Centre Hospitalier Universitaire de Nantes, Nantes, France; 10 Pediatric Department, Centre Hospitalier Universitaire de Caen, Caen, France; 11 Pediatric Department, Centre Hospitalier Universitaire de Bordeaux, Département de Pédiatrie, Centre d'Investigation Clinique (CIC 0005), F-33000, Bordeaux, France; 12 Pediatric Pulmonary Department, Centre Hospitalier Universitaire de Marseille, Marseille, France; 13 Pediatric Department, Centre Hospitalier Universitaire de Nice, Nice, France; 14 Pediatric Pulmonary Department, Centre Hospitalier Universitaire de Toulouse, Toulouse, France; 15 Centre Hospitalier Universitaire de Reims, Reims, France; 16 Pediatric Department, Centre Hospitalier Universitaire de Nancy, Nancy, France; 17 Pediatric Department, Centre Hospitalier Universitaire de Besançon, Besançon, France; 18 Pediatric Department, Centre Hospitalier Universitaire de Rouen, Rouen, France; 19 Pediatric Pulmonary Department, Hôpital Robert Debré, Paris, F-75019, France; 20 Pediatric Department, Centre Hospitalier Universitaire d’Angers, Angers, France; 21 Pediatric Department, Centre Hospitalier de Lens, Lens, France; 22 Pediatric Department, Centre Hospitalier Universitaire de Grenoble, Grenoble, France; 23 Pediatric Department, Centre Hospitalier Universitaire de Tours, Tours, France; 24 Inserm UMR S-707, Paris, F-75012, France

**Keywords:** Interstitial lung disease, Network, Epidemiology, Database

## Abstract

**Background:**

Interstitial lung diseases (ILDs) in children represent a heterogeneous group of rare respiratory disorders that affect the lung parenchyma. After the launch of the French Reference Centre for Rare Lung Diseases (RespiRare®), we created a national network and a web-linked database to collect data on pediatric ILD.

**Methods:**

Since 2008, the database has been set up in all RespiRare® centres. After patient's parents' oral consent is obtained, physicians enter the data of children with ILD: identity, social data and environmental data; specific aetiological diagnosis of the ILD if known, genetics, patient visits to the centre, and all medical examinations and tests done for the diagnosis and/or during follow up. Each participating centre has a free access to his own patients' data only, and cross-centre studies require mutual agreement. Physicians may use the system as a daily aid for patient care through a web-linked medical file, backed on this database.

**Results:**

Data was collected for 205 cases of ILD. The M/F sex ratio was 0.9. Median age at diagnosis was 1.5 years old [0–16.9]. A specific aetiology was identified in 149 (72.7%) patients while 56 (27.3%) cases remain undiagnosed. Surfactant deficiencies and alveolar proteinosis, haemosiderosis, and sarcoidosis represent almost half of the diagnoses. Median length of follow-up is 2.9 years [0–17.2].

**Conclusions:**

We introduce here the French network and the largest national database in pediatric ILDs. The diagnosis spectrum and the estimated incidence are consistent with other European databases. An important challenge will be to reduce the proportion of unclassified ILDs by a standardized diagnosis work-up. This database is a great opportunity to improve patient care and disease pathogenesis knowledge. A European network including physicians and European foundations is now emerging with the initial aim of devising a simplified European database/register as a first step to larger European studies.

## **Introduction**

Interstitial lung diseases (ILDs) in infants and children represent a heterogeneous group of rare and complex respiratory disorders that affect the parenchymal part of the lung, and are mostly chronic [1].

Disease complexity is reflected by the various underlying mechanisms initiated by the primary causes of injury. As a result, a major concern is the difficulty to define and to identify ILDs in children and especially in infants. A rigorous analytical process is required to explore correlation between pathological determinants and patient’s phenotypes, which in turn is essential to progress in disease classification and understanding.

ILDs are considered as rare diseases. In a growing number of countries worldwide, rare diseases have been recognized as a crucial health issue, and strategies are developed to implement appropriate public policies for patient care and research. In France, rare diseases have also been identified as a priority of public health needs, and among the various actions launched; a National Reference Centre for Rare Lung Diseases (RespiRare®, http://www.respirare.fr) was created in 2006 to care for all children in the country suspected or known to have one of them, including ILDs. The main objective of RespiRare® is to establish disease cohorts based on aggregation of patients followed over time, through a national organization with affiliated centres that was set up to cover the entire French pediatric population with respiratory diseases. For ILDs, a platform was built to provide appropriate resources to pediatricians to record patient information and to ensure prospective clinical data collection. After data standardization and monitoring for quality control, data is stored into a national ILD database through a web interface. The RespiRare® platform, which combines clinical, biological, functional and genetic information, is a unique national structure for the emergence of prospective registries and databases allowing the development of epidemiological, clinical and translational research studies.

Information provided herein describes the ILD database of the RespiRare® platform. Analyses of the data currently included are presented, as well as the potential for international collaborative studies.

## **Materials and methods**

### **Database structure and quality control**

The RespiRare® database structure for rare respiratory diseases was created to include extensive information on all forms of rare lung diseases grouped within four main categories: ILD, respiratory malformations, ciliary dyskinesia, and other rare diseases with chronic respiratory insufficiency. Data from patients is entered via a secured Internet protocol into a safe database through a web interface. The interface is visible from the web through the https secure protocol, on a DMZ network. It uses the LAMP system, with a MySQL database for data storage; PHP scripts interface for access to database, as well as a Apache 2 webserver. The structure is hosted on Linux servers, within the secure server network of Paris-6 University Pierre et Marie Curie (UPMC), the coordinator affiliated institution. The database server hosting the MySQL server is behind a firewall, linked to the webserver through a secure encrypted IP to IP tunnel. Access to the patient is filtered through a central identification number. Database and data collection have been approved by the French national data protection authorities: “Commission Nationale de l’Informatique et des Libertés” and the “Comité Consultatif sur le Traitement de l'Information en matière de Recherche dans le domaine de la Santé”.

The Reference centre and 8 affiliated centres in university hospitals (called Competence Centres) are spread all around the French national territory. Each of them covers a French area with a local network of affiliated centres (Additional file 1). Every participating centre only has access to their own patients’ data. For cross-centre studies, the respective centres must agree explicitly to share its anonymous data with other centres. A charter describes the rules, including general rules relating to organization and rules governing access to data. Each participating member is asked to accept the charter of good use. To access the database, users receive a personal user name and password. Users can see who modified the file, and if any modification is observed in data recording, a tracking system is available to get back to a previous version of the data. Patients must give their oral informed consent before their data is included into the database.

The quality of the data relies on the users who enter the data, and on a database scientific committee who regularly reviews proposed cases for inclusion. The RespiRare® administration offers each centre a specific training for proper use of the on-line database. A data manager and a technical team are in charge of quality control, monitoring for data coherence, absence of duplicates, and transfer of data if needed. The data is organized in a data warehouse to optimize data visualization and data retrieval.

To maintain motivation and to ensure long-term involvement of all participants, specific contacts with the coordinating team through e-mail, phone calls and local visits have been put in place. In addition, an annual meeting is organized with all affiliated centres.

### **Data collection and analysis**

The database we have created at the national level in France was elaborated by a team composed of clinicians and databank professionals, using data modelling techniques and methodologies with a Conceptual Data Model (CDM) providing a view of the key data entities and their relationships (Additional file 2). The RespiRare® database is structured as medical record for patients. Users can document a large amount of data: identity, social data, environmental data, and diagnosis of the ILD if known, genetic data, patients visits to the centre, and the entire medical examinations and tests done for the diagnosis or the follow up of the patient. Data can be entered at every visit of the patient or at least once a year in a minimal dataset containing an “annual form” and a “diagnosis form”, which is filled at the first visit and can be updated at any time (Additional file 3). The datasets for the 3 other disease groups (respiratory malformations, ciliary dyskinesia, and other rare diseases with chronic respiratory insufficiency) are organized in a similar way, with a diagnosis dataset and a follow-up dataset (on an annual basis). A common list of items for all groups includes clinical, functional, imaging, organ specific tests, treatments and outcome information. Extended disease-specific datasets contain information relevant to each group of rare diseases. The entire ILD French database dictionary is provided in the Additional file 4. These extended datasets have been defined by steering committees composed of experts in the respective groups of diseases. They are implemented continuously in order to provide extensive and detailed documentation of a given disease. The database also offers a system for inclusion of biological, microbiological, radiographic, histological, cytogenetic, molecular and genetic information.

The database is designed for a long-term use. In this view, the interface has been developed so that it is easy to use and friendly, with rolling menus and data entry forms accessible to unskilled users. In addition, RespiRare® database has been organized and structured as a medical record for patients. Consequently, physicians can use the system as a daily aid for patient care through a web accessible medical record backed on this database. It takes around 20 minutes to fill the form at the first visit and around 5 minutes at every additional visit.

### **ILD Patients**

Based on our previously described ILD classification (Clement *et al.* Orphan J Rar Dis 2010), all patients eligible to one of the ILD diagnosis can be proposed for inclusion in the database (Table 1). Moreover, if no diagnosis is known, any patient under 18 years old with a persistent interstitial pathway on CT-scan can also be included in the database. Acute interstitial diseases are not included. The diagnosis is based on clinical, radiological, and functional features, including tachypnea, diffuse infiltrates on chest imaging and impaired gas exchange. Written information is given to the patient/family before documenting the database. The physician of the Competence Centre in charge can recommend that patients be sent to the Reference Centre for an exam or a specific investigation.

**Table 1:** Interstitial lung diseases diagnoses proposed in the database

**Exposure related ILD**	Hypersensitivity pneumonitis
	Aspiration pneumonitis
	Medication or drug exposure
	Others
**Systemic disease-associated ILD**	Connective tissue diseases
	- Rheumatoid arthritis
	- Systemic sclerosis
	- Systemic lupus erythematosus
	Pulmonary vasculitis
	- Wegener’s granulomatosis
	- Churg-Strauss syndrome
	- Anti-glomerular basement membrane disease
	- Langerhans’ cell histiocytosis
	Others
**Granulomatous diseases**	Sarcoidosis
	Crohn disease
	Others
**Metabolic disorders**	Lysosomal diseases
	- Niemann-Pick diseases
	- Hermansky-Pudlak syndrome
	- Gaucher's disease
	Familial hypercalcemia with hypocalciuria
	Others
**Infectious ILD**	Bacterial infection
	Chlamydiae infection
	Mycoplasma infection
	Viral infection
	Others
**Alveolar disorder related ILD**	Eosinophilic lung diseases
	Alveolar microlithiasis
	Surfactant disorders
	Pulmonary alveolar proteinosis
	Others
**Alveolar vascular disorder related ILD**	Pulmonary capillary haemangiomatosis
	Diffuse alveolar haemorrhage and haemosiderosis
	Alveolar capillary dysplasia
	Others
**ILD specific to infancy**	Pulmonary glycogenosis
	Neuroendocrine cell hyperplasia of infancy
**ILD with no diagnosis**	

## **Results**

### **Pediatric ILD patients**

The RespiRare® database was launched in September 2008. The database was set up at the end of 2008. After their initial training, centres started to include patients one by one. At the beginning, after the database committee validation, centres included all patients with ILD less than 18 years old currently followed in the centres, with their entire past medical records. New patients were also included in the database in a prospective way. Thus, the database covers up to 17 years for some patients followed for a long time at the time of the database creation. This is an on-going process, and centres’ ability to propose patients for inclusion in the database is still variable in terms of speed. Multiple biases, such as recovered or deceased patients, cannot let us talk yet about prevalence or incidence.

At the present time, 217 patients were proposed for inclusion in the database. The database committee rejected 12 patients because the diagnosis of ILD wasn’t proven, or because insufficient data was provided. Thus, the number of ILD patients included in the database is now 205.

The study is presented to all patients/families during a medical visit. None of the family has refused to be enrolled. Of importance also is the observation that many physicians in charge of the patients consider this structured data collection of great help. Indeed, as mentioned above, in an increasing number of French pediatric pulmonology teams, RespiRare® database is now used as a medical record for patient care.

### **Sex and age distribution**

**Figure 1:**
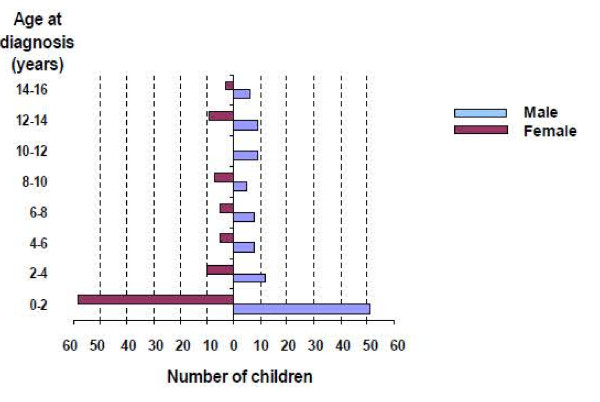
Age at diagnosis of interstitial lung disease according to sex.

Considering the 205 ILD children entered in the database, the sex ratio is 0.9 boy/girl. The median age at diagnosis is 1.5 years old [0–16.9]. More than half of ILD start before 2 years old (Figure 1). Median diagnostic delay is 1 year [0–12.6], largely influenced by the type of ILD. The median length of follow up into the database is currently 2.9 years [0–17.2].

### **Diagnosis distribution**

Aetiology for the ILD has been identified for 149 (72.7%) patients whereas 56 (27.3%) patients remain with no identified cause. Surfactant deficiencies and alveolar proteinosis, haemosiderosis and sarcoidosis, represent almost half of the diagnoses. The diagnosis breakdown, age at diagnosis, sex ratio and length of follow up are detailed on Table 2.

**Table 2:** Aetiologic spectrum of interstitial lung disease (ILD) in children

**Diagnoses**	**Patients (n=)**	**Patients (%)**	**Sex ratio M/F**	**Median age at diagnosis (years)**	**Range**
**Surfactant disorders**	36	17.6	1.0	0.3	(0–12.3)
**Haemosiderosis**	23	11.2	0.3	4.8	(0.7-14)
**Granulomatous diseases (sarcoidosis)**	21	10.2	1.0	9.6	(5.6-14.4)
**Alveolar proteinosis**	20	9.7	1.8	0.6	(0–13.6)
**Exposure diseases**	9	4.4	0.8	10.6	(2.2-14.4)
**Connective disorders and vascularitis**	9	4.4	0.6	12.4	(0.1-15.7)
**Metabolic disorders**	5	2.4	0.7	1.7	(0–4)
**Langerhans' cell histiocytosis**	5	2.4	1.5	1.1	(0.5-7.3)
**Infectious diseases**	4	2.0	1.0	0.2	(0–6.6)
**Eosinophilic lung diseases**	4	2.0	1.0	10.0	(5.1-16.9)
**Lymphatic disorders**	4	2.0	0.3	0	(0–14.5)
**Others**	9	4.4	2.0	1.3	(0–12.8)
**Undiagnosed**	56	27.3	1.0	0.7	(0–16.4)
**Total**	**205**	**100**	**0.9**	**1.5**	**(0–16.9)**

Abbreviations: M: male; F: female.

### **Surfactant disease and alveolar proteinosis**

For 36 children (17.5%), a surfactant disorder has been identified. These children have an early median age of diagnosis (0.3 year-old). Pulmonary surfactant-associated protein C gene (*SFTPC)*, Pulmonary surfactant-associated protein B gene (*SFTPB)*, ATP-binding cassette subfamily A, member 3 gene (*ABCA3)*, and Thyroid transcription factor-1 gene (*TITF1 = NKX2-1)* mutations were found (Table 3).

**Table 3:** Characteristics of patients with surfactant disorders

**Patient number**	**Sex**	**Age at diagnosis (years)**	**Follow up (years)**	**Surfactant disorder**	**Mutation**
1*	M	0.3	2.9	SP-C	c.424delC
2*	M	0.4	3.6	SP-C	c.566 G > A (C189Y)
3*	F	0.3	3.8	SP-C	c.566 G > A (C189Y)
4*	M	0.2	1.4	SP-C	c.325-1 G > A
5*	M	2.4	0.4	SP-C	c.218 T > C (I73T)
6*	F	5.8	0	SP-C	c.218 T > C (I73T)
7*	F	1.6	2.4	SP-C	c.218 T > C (I73T)
8*	F	0.4	1.4	SP-C	c.218 T > C (I73T)
9*	F	1.2	1.2	SP-C	c.218 T > C (I73T)
10*	M	0.8	2.5	SP-C	c.218 T > C (I73T)
11*	F	0.3	4.2	SP-C	c.218 T > C (I73T)
12*	F	0	1.8	SP-C	c.218 T > C (I73T)
13*	M	1.6	md	SP-C	c.218 T > C (I73T)
14*	M	0.7	4.2	SP-C	c.218 T > C (I73T)
15*	M	2.2	3.1	SP-C	c.218 T > C (I73T)
16*	F	0.2	6.2	SP-C	c.218 T > C (I73T)
17*	F	2.7	11.7	SP-C	c.218 T > C (I73T)
18*	F	0.5	12.4	SP-C	c.218 T > C (I73T)
19*	M	0.4	6.6	SP-C	c.218 T > C (I73T)
20*	M	0.3	2.4	SP-C	c.581 T > C (L194P)
21*	M	8.5	0	SP-C	c.563 T > C (L188P)
22*	F	0.4	17.2	SP-C	c.116 T > C (V39A)
23	M	0	0.1	SP-B	g.1549 C > GAA (121ins2) −/−
24	M	0	0.1	SP-B	g.1549 C > GAA (121ins2) −/−
25	M	0.2	0	SP-B	g.1549 C > GAA (121ins2) −/−
26	F	0	0.2	SP-B	g.1549 C > GAA (121ins2) −/−
27	M	0.1	0	SP-B	c.496delG −/−
28	M	0	0.1	SP-B	Y253X −/−
29	F	0	0.2	SP-B	672 + 2del5 −/−
30*	F	0	9.2	ABCA3	c.3518 C > G (T1173R) −/−
31*	M	0.3	10.9	ABCA3	c.757 G > C (D253H) −/−
32*	F	2.3	MD	ABCA3	c.3518 C > G (T1173R) −/−
33*	M	0.3	3.4	ABCA3	P585 −/−
34*	F	12.3	3	ABCA3	c.3518 C > G (T1173R) −/−
35*	F	0.1	4.8	TTF1	c.786_787del2 (p.L263fs)
36*	F	0.1	1.5	TTF1	c.493 C > T(R165W)
	**Sex ratio 1**	**Median 0.3**	**Median 2.4**		

* Published patients.

Abbreviations: M: male. F: female. MD: missing data.

*SFTPC* mutations are the most frequent genetic abnormalities (22 patients), representing 10.7% of all ILD patients. The recurrent mutation I73T represents two thirds of them. The median age at diagnosis is 6 months [0–8.5]. Lung biopsy was performed for 14 children, showing an alveolar wall thickening, type 2 alveolar epithelial cells hyperplasia, inflammation cells infiltration with or without macrophagic intra-alveolar accumulation with cholesterol crystals and occasionally fibrosis. All patients received steroids. Among them, nineteen patients underwent intravenous steroid pulses and azithromycin. Two patients died at 3 and 17 years old. Seven patients presenting neonatal respiratory distress had *SFTPB* homozygous mutations. All of them died before 2 months of life. *ABCA3* homozygous mutation was found in 5 patients with neonatal respiratory failure. Median age at diagnosis was 0.3 years old [0-12.3]. They all needed long-term steroid pulses. Two patients showed a brain lung thyroid syndrome with *TITF1/NKX2-1* mutation. One of them died at month 18.

Twenty patients were diagnosed with alveolar proteinosis (sex ratio = 1.9 boy/girl). Onset of symptoms was very early with a median age at diagnosis of 7 months. Most of them needed therapeutic broncho-alveolar lavages (BAL).

### **Haemosiderosis**

We collected data of 23 haemosiderosis (Table 4). There was a large predominance of girls (18 *vs.* 5 boys) and the median age at diagnostic was 4.8 years old [0.7-14]. Three of them were associated with another disease (cow proteins milk allergy, celiac disease, Down syndrome). Initial presentation was mainly acute with haemoptysis and major anaemia but some cases were more difficult to diagnose with cryptic chronic bleeding and no haemoptysis. Pulmonary hypertension was reported for only 2 children. Nine children underwent open lung biopsy or transbronchial biopsy to confirm the diagnosis. All children benefits from a steroid treatment, with steroid pulses for 11 of them. Four patients benefit from supplemental treatment by hydroxychloroquin and 4 from mycophenolate mofetil.

**Table 4:** Characteristics of haemosiderosis patients

**Patient number**	**Sex**	**Age at diagnosis (years)**	**Follow up (years)**	**Diagnosis based on**
1	F	2.3	1.3	BAL and LB
2	M	1.4	15.6	BAL and LB
3	F	3.2	13.2	BAL and LB
4	M	1.1	13.7	BAL and LB
5	M	5.4	9.8	BAL
6	F	7.2	10.8	BAL and LB
7	F	6.7	7.4	BAL
8	F	4.7	7.3	BAL and LB
9	F	12.3	MD	MD
10	F	4.8	2.3	BAL
11	F	10.6	5.8	BAL
12	F	5.1	4.8	BAL
13	M	1.4	4.6	BAL
14	F	7.2	4.7	BAL
15	M	14	3.8	BAL
16	F	0.8	2	BAL
17	F	0.7	2.6	BAL and LB
18	F	1.9	3.5	BAL and LB
19	F	7.2	0.6	BAL
20	M	2.3	2.7	BAL
21	F	1.9	MD	BAL and LB
22	F	6	MD	MD
23	F	11.6	0.5	BAL
**Total**	**Sex ratio 0.3**	**Median 4.8**	**Median 4.7**	**BAL = 21**
				**LB = 9**

* Published patients.

Abbreviations: M: male. F: female. MD: missing data. BAL: broncho-alveolar lavage. LB: lung biopsy.

### **Sarcoidosis**

Twenty-one children had interstitial granulomatous lung disease, which was for all of them a sarcoidosis (Table 5). Sex ratio was 1 boy/girl and median age at diagnosis was 9.4 years old. Sarcoidosis is one the most frequent pre-teenager ILD diagnosis with connective lung diseases and exposure related ILD. BAL was performed for 15 of them and showed in most of the cases a lymphocytic infiltration. CD4/CD8 ratio was available for 7 patients and was >1 for 6 of them. Five patients underwent transbronchial or open lung biopsy to assert diagnosis. All patients were treated with steroids and among them, 11 received steroid pulses.

**Table 5:** Characteristics of sarcoidosis patients

**Patient number**	**Sex**	**Age at diagnosis (years)**	**Follow up (years)**	**BAL lymphocytes (%)**	**Organs involved (biopsied organ)**
1*	M	6.5	9.4	42	Lung, lymphadenopathy
2	M	8.0	3.6	48	Lung, lymphadenopathy, eye (uveitis)
3	F	13.8	2	64	Lung, liver
4	M	13.5	3.8	69	Lung, lymphadenopathy, liver
5	M	12.7	4.2	28	Lung, lymphadenopathy
6	F	8.2	2.7	49	Lung, lymphadenopathy, bone marrow, spleen
7	F	7.1	2.4	30	Lung, lymphadenopathy, bowel
8	F	11.2	4	MD	Lung, skin
9	M	14.4	3.1	20	Lung, lymphadenopathy, parotid, eye, joints
10	M	5.9	6.7	29	Lung, skin, spleen
11*	F	9.1	9.1	59	Lung, lymphadenopathy, liver, eye
12	F	13	3.3	MD	Lung, eye, liver, kidney, skin
13	F	11.5	2.6	72	Lung, joints, lymphadenopathy
14	F	10.8	2	12	Lung
15	M	12.8	2.5	18	Lung, liver, skin, eye, bone marrow
16	M	5.6	5.2	MD	Lung, larynx, tonsils
17	F	14	4	MD	Lung, joints, eye
18	F	9.6	0.8	23	Lung, liver, lymphadenopathy
19	F	8.3	0.4	MD	Lung
20	M	9	9.2	50	Lung
21	M	9.3	MD	MD	Lung, lymphadenopathy, central nervous system
**Total**	**Sex ratio 1**	**Median 9.4**	**Median** **3.4**		**Restricted to the lung = 3, multi-organic = 18**

* Published patients.

Abbreviations: M: male. F: female. MD: missing data. BAL: broncho-alveolar lavage.

### **Other diagnosis**

Nine children had exposure related ILD: 5 had hypersensitivity pneumonitis (turtledove, pigeon, straw), 1 was due to total body irradiation, and the causal agent was not specified for the others. Outcomes were good with no residual symptoms after eviction and, for some of the patients, short steroid therapy.

Connective lung diseases were diagnosed for 9 children (rheumatoid arthritis, systemic sclerosis, mixed connective tissue disease). Three children presented with vasculitis of unknown origin. No capillaritis has been included in the database at this time. All together, there are twice as many girls than boys and teenagers are largely represented, but 3 children were diagnosed before 5 years old.

ILD related with metabolic disorders were diagnosed for 5 children. Among them, 4 had a Niemann-Pick B or C disease and one had a Cobalamine C deficiency.

Five children had Langerhans’ cell histiocytosis with ILD. All but one had a multifocal disease with bone involvement.

Infectious diseases leading to ILD was diagnosed for 4 children and attributed to *Chlamydiae trachomatis* for 2 of them. All of them were free of symptoms at the end of the follow up (after 6 month to 3 years).

We also collected data for 4 eosinophilic lung diseases and 4 lymphatic disorders.

Nine children presented an ILD related to others diagnosis: Crohn disease (n = 1), inhalations (n = 1), alpha-1-antitrypsin deficiency (n = 1), xanthogranulomatous disease (n = 1), constrictive bronchiolitis (n = 1), hamartoma (n = 2), others (n = 2).

### **No diagnosis**

Finally, out of 205 patients presenting with ILD, 56 children (27.3%) remained with no diagnosis (Additional file 5). Sex ratio is 1 male/female and median age at diagnosis is young: 0.7 years [0–16.4]. Of importance is the observation that 34.5% of children with ILD diagnosed before 2 years old remain with no diagnosis whereas it is so for 19.1% of ILD diagnosed after 2 years old. For a large part of them, the absence of diagnosis was concluded after a number of investigations, including genetic studies (n = 40) and BAL (n = 27). Only 22 of them underwent lung biopsy. However, some patients did not benefit from these explorations, or are missing data, so that the absence of diagnosis cannot be ascertained.

## **Discussions**

In the present article, we describe the first national internet-based patient database for pediatric ILD. Analysis of the collected data provides information on the current diagnosis distribution of these heterogeneous disorders.

The need for a centralized database was clearly identified, with the main concern being how to build its infrastructure. Indeed, the system would have to serve clinicians treating the patients, researchers for conducting national and international programs, epidemiologists to gather demographic data, patients and their families seeking accurate information on their disease, as well as the pharmaceutical industry expressing increased interest for rare disease databases. At the present time, the system includes personal/demographic data and clinical information.

As for other patient registries and central databases, the major challenge is data standardization and quality. Data quality is the cornerstone of the registry system, and we are extremely focused on quality control and data accuracy. As indicated above, the RespiRare® system is headed by the reference centre in close connection with the affiliated centres in French university hospitals. After several months of development, involvement of affiliated centres is increasing. To ensure data quality, an intensive monitoring has been organized with the coordinating team and a database committee, that review all cases proposed for inclusion in the database. To facilitate switch from usual paper-based documentation and on-line implementation, several solutions, depending on local possibilities have been set up. Of major interest for improving data accuracy is the progressive use by physicians of the system as a daily aid for patient care through a web accessible medical record backed on the database.

Since its creation, RespiRare® has made significant efforts to collect valuable information on ILD and other rare lung diseases. Certainly, a major achievement has been the ability to gather and connect the entire medical teams involved in the network. Among the tasks in progress, current efforts include the standardization of diagnoses and algorithm for explorations in strong connection with Orphanet, as well as the definition of minimal datasets that need to be collected for evaluation of disease progression, and the link with biospecimen repositories for the development of clinical and research studies.

Obviously, our database is not a register and presents multiple selection bias. The fist one is based on the fact that physicians are responsible for the allocation of patients and can omit to propose some of them for inclusion. The second bias is that the participating physicians are pediatric pulmonologist, who are not working in intensive care units (ICU). Thus, extremely severe neonatal or pediatric cases leading to death, where RespiRare® physicians were not consulted are not included in the database. In order to improve this bias, we are currently reinforcing links between pulmonologists and local ICU physicians. Very mild ILD diseases can also represent a bias given that they are not always referred to a reference centre. Finally, children above 15 years old can, in France, be referred to an adult pulmonologic department. Our pediatric network can also miss those patients, despite our focused on reinforcing links with adult teams. As a result, our database is not exhaustive, and cannot at this time provide an incidence or prevalence. However, we are working hard to promote the database in France and Europe.

We introduce here the largest national database for children ILD. Information available in the database includes diagnosis breakdown, phenotypic data on age at diagnosis, date of diagnosis, length of follow up, clinical presentation, BAL, functional tests, genetic tests and lung biopsy. The number of patient entries is growing continuously [2]. Our present data confirms similarities with the 2 other ILD databases in Europe, which are currently used in Germany and United Kingdom. The first one reviews all diffuse parenchymal lung diseases (DPLD) in children in Germany and describes 38 DPLD children [3]. The calculated incidence was 1.32 cases per 1 million of children per year, which is close to our estimations. We observe a comparable sex ratio around 0.9 male to female and a large part of onset of disease before 2 years old (one third before 1 year old in the German cohort and 42% in this study).

The UK database (BPOLD) focuses on 9 diseases including ILD, pulmonary alveolar proteinosis, pleural and pulmonary lymphangiectasia, and idiopathic pulmonary haemosiderosis. All together, it includes 29 children [4,5]. The described diagnosis breakdown of ILD is similar to the German study and confirms that the main diagnoses of ILD are surfactant disorders, haemosiderosis, granulomatous diseases (sarcoidosis) and alveolar proteinosis [1,3].

Regarding surfactant disorders, we confirm that *SFTPC* mutation I73T (c.218 T > C) is the more prevalent mutation, but numerous other mutations have been described [6,7]. Recently, *SFTPC* mutations have been described in an adult cohort of familial idiopathic fibrosis [8]. This observation together with the extremely heterogeneous phenotypes of SP-C deficiency, ranging from early fatal respiratory failure to children and adults chronic respiratory disease should encourage to enlarge indications of *SFTPC* sequencing in children and adults with chronicle respiratory symptoms and alveolo-interstitial pattern on CT-scan [7,9-11]. Recessive mutations in the *ABCA3* gene were first attributed to fatal respiratory failure in term neonates but are increasingly being recognized as a cause of ILD in older children and young adults [12]. Over 100 *ABCA3* mutations have been identified in neonates with respiratory failure and in older children with ILD [13-20]. The 5 children we report here are still alive and the oldest is now 16 years old. Our 7 diagnosed children for SP-B deficiency died within the first days of life in a refractory hypoxemia condition. Mutations in the *TITF-1* gene have been associated with “brain-lung-thyroid syndrome” [21-27]. We report 2 children with this extremely rare disease, of which only one is still alive at 5 years old. So far, few mutations have been reported, mostly in exon 3 [28,29].

The present study reports the largest pediatric haemosiderosis cohort with 23 cases. Haemosiderosis is a diagnosis of exclusion of diffuse alveolar haemorrhage syndromes (DAH) based on patient presentation with acute, subacute, or recurrent DAH, on the results of BAL showing more than 10% siderophages and lung biopsy showing evidence of ‘bland’ pulmonary haemorrhage (i.e., without capillaritis or vascularitis) [1,30]. When diagnosing idiopathic pulmonary haemosiderosis in a child, particular attention has to be paid to diseases induced by environmental factors such as pesticide and cow’s milk (Heiner’s syndrome) [31]. One child in our study presented this particular acute situation. After the initial potential extreme anaemia, outcome is good in children with steroid treatment. Some children in our study needed additional hydroxychloroquin treatment as it is recommended for adults’ haemosiderosis. No capillaritis is accounted for in the database. Vascularitis related ILD are extremely rare in children, but are probably under-diagnosed. This database is a starting point and enables us to outline missing data. This dynamic process is ongoing and efforts are currently made to sensitize physicians to recognize and to include those patients.

Sarcoidosis represents also a large part of ILD in children. We report here 21 pediatric sarcoidosis, which are mostly teenagers. Clinical findings are various, depending on the different organ involvement [1,32-36]. We also confirm that lung function abnormalities are frequently observed in children with restrictive pulmonary pattern and abnormal diffusing capacity [37]. All children with sarcoidosis were treated with steroids. This probably highlights that only the most severe sarcoidosis were included by physicians in the database.

Of importance, the present study highlights that more than a quarter of ILDs remain with no diagnosis, and the number is a third for patients included before 2 years old. This is in agreement with other previous reports. Undiagnosed cases are heterogeneous in terms of initial presentation, follow-up and management. An important challenge is certainly to reduce the number of unclassified ILDs, especially in youngest children, maybe with a precise phenotypic description and a better frequency and observation of lung biopsy. In this perspective, a more systematic approach to diagnostic is required, ensuring that the entire appropriate examinations currently available have been preformed at presentation and during the follow-up. Although huge efforts have been made in genomics, the phenotypic description of ILD cohort is still insufficient [38]. To progress, a well-implemented database with biologic, functional and histological markers represents an essential step for a large phenomic study on pediatric ILD.

To conclude, we introduce here the French network for rare lung diseases and the first national Internet based database for pediatric ILD. The database is now prospectively growing and more accurate data is being implemented in order to get better phenoms of these rare lung diseases. Moreover, a biologic bank related to the web-based database is currently being created. This database is a great potential for improving patient care and disease understanding. A European network of physicians, scientists and families’ foundations is currently emerging and we are working, together with the US ChILD foundation on a simplified European database register as a first step to European large studies [39-41].

## **Competing interests**

The author(s) declare that they have no competing interests.

## **Authors’ contributions**

NN and AC drafted the manuscript, with DM and JFV’s contribution for the database chapter. All authors contributed to enter patients’ data in the RespiRare® database and to check their data before publication. AC, JdB and RE provided a special contribution to the data and the manuscript correction, as heads of the Reference Centre. All authors read and approved the final manuscript.

## Supplementary Material

Additional file 1: **S1. The French Reference Centre for Rare Lung Diseases (RespiRare®) network.** Each colour represents one of the 9 French areas of RespiRare® with its affiliated centres, related to their Competence Centre. The Reference Centre (Paris) is responsible for all Competence Centres. Legend: **PARIS:** Reference, Centre **Marseille**: Competence Centre, **Angers**: Affiliated Centre. Click here for file

Additional file 2: **S2. Conceptual Data Model (CDM) for the French interstitial lung diseases database.** The diagram illustrates the way data models are developed based on the data requirements for the ILD program, with entity types, attributes, and relationships. Click here for file

Additional file 3: S3. Essential dataset for interstitial lung disease. Click here for file

Additional file 4: **S4. Extensive interstitial lung disease (ILD) database dictionary (in French).** Common dataset to the 4 groups of diagnosis (interstitial lung diseases, respiratory malformations, ciliary dyskinesia, and other rare diseases with chronic respiratory insufficiency) are highlighted in yellow. Click here for file

Additional file 5: S5. Characteristics of interstitial lung disease (ILD) patients with no precise diagnosis. Click here for file
