# Supplementary figures and images for: A national internet-linked based database for pediatric interstitial lung diseases: the French network
Source: Orphanet J Rare Dis. 2012 Jun 15;7:40. doi: 10.1186/1750-1172-7-40 (PMC3458912; doi:10.1186/1750-1172-7-40)

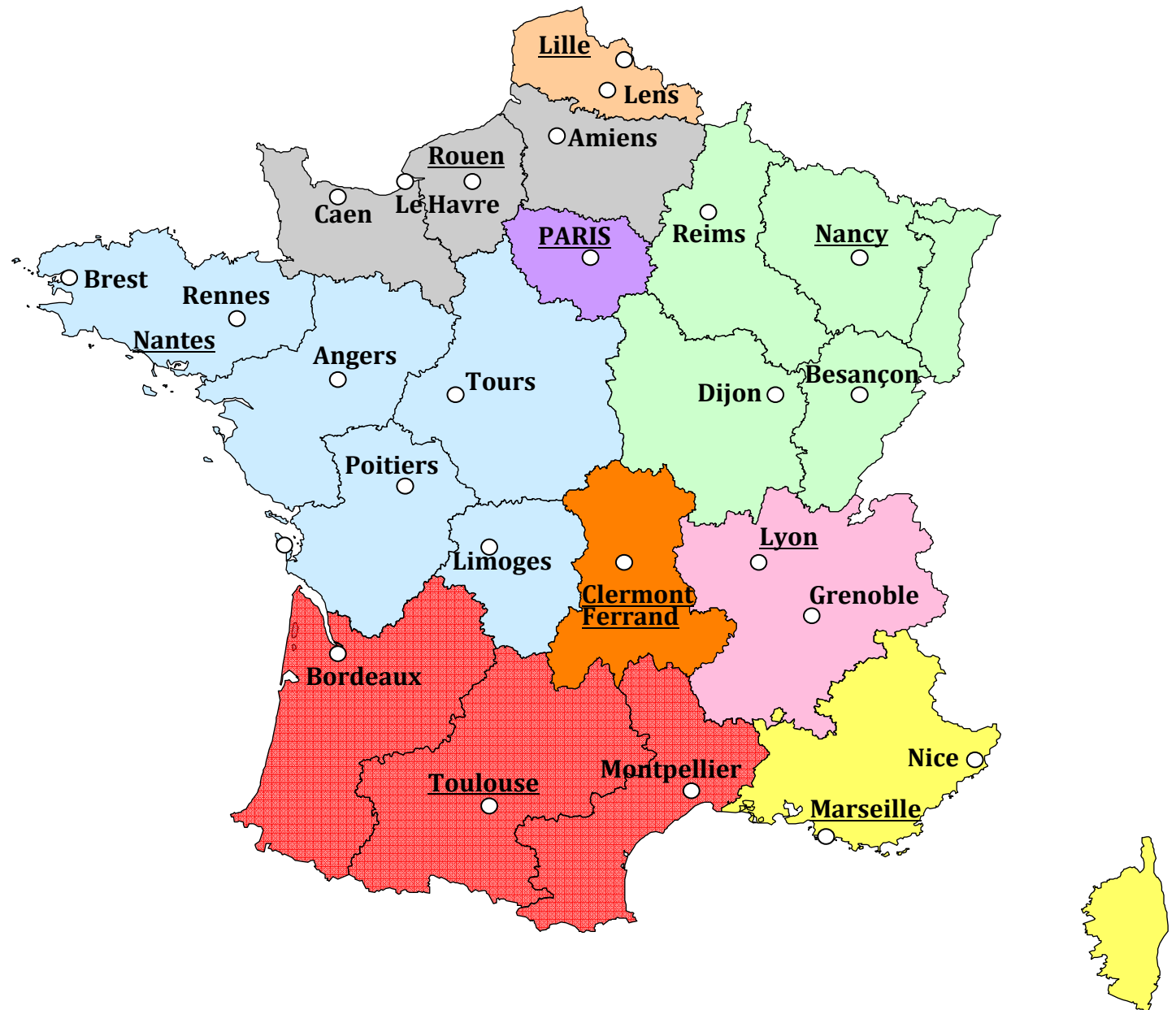

Supplement: Additional file 1: — S1. The French Reference Centre for Rare Lung Diseases (RespiRare®) network. Each colour represents one of the 9 French areas of RespiRare® with its affiliated centres, related to their Competence Centre. The Reference Centre (Paris) is responsible for all Competence Centres. Legend: PARIS: Reference, Centre Marseille: Competence Centre, Angers: Affiliated Centre. [file 1750-1172-7-40-S1.pdf]

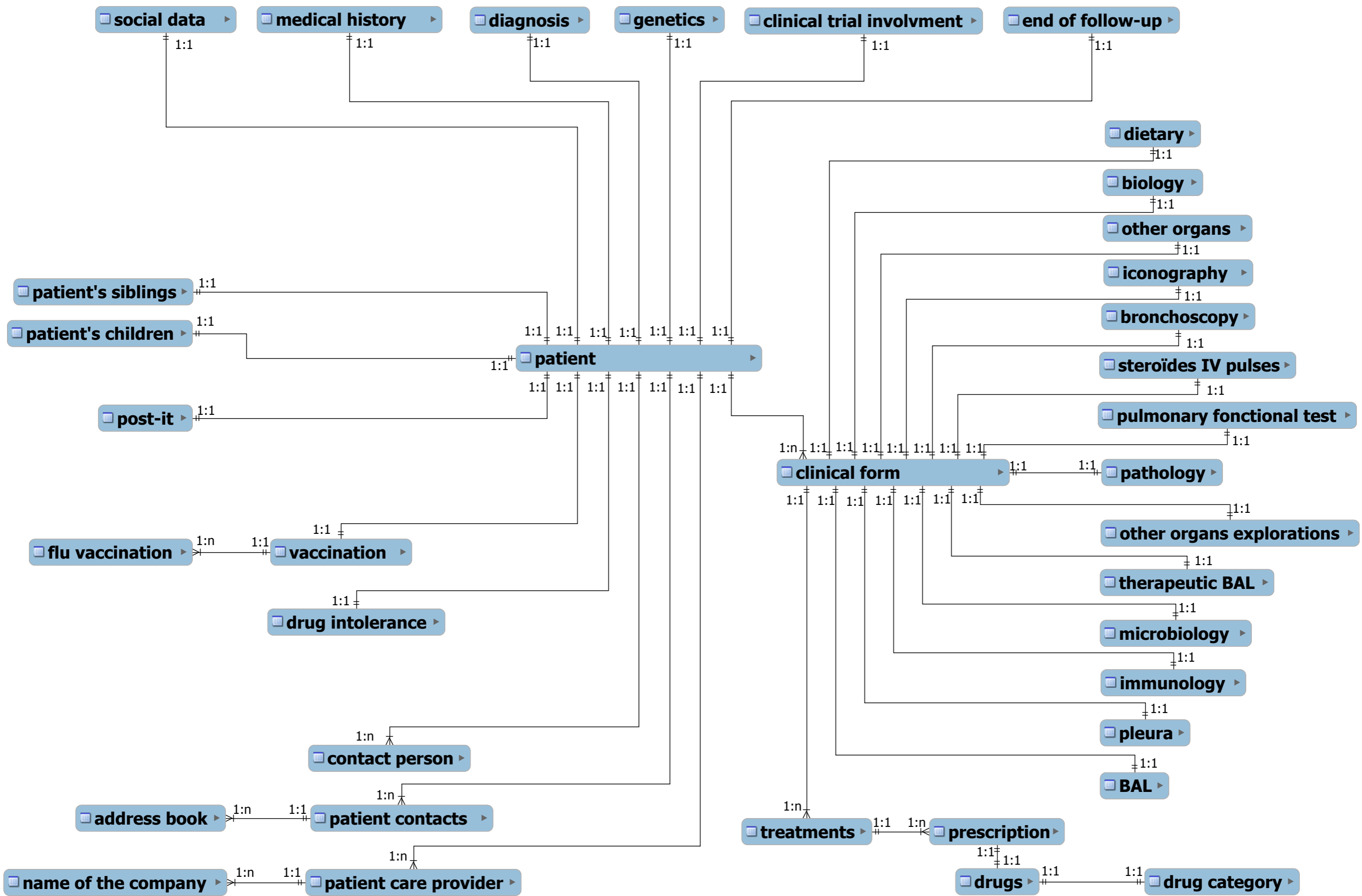

Supplement: Additional file 2: — S2. Conceptual Data Model (CDM) for the French interstitial lung diseases database. The diagram illustrates the way data models are developed based on the data requirements for the ILD program, with entity types, attributes, and relationships. [file 1750-1172-7-40-S2.pdf]
