# Supplementary material for: A national internet-linked based database for pediatric interstitial lung diseases: the French network
Source: Orphanet J Rare Dis. 2012 Jun 15;7:40. doi: 10.1186/1750-1172-7-40 (PMC3458912; doi:10.1186/1750-1172-7-40)
Supplement: Additional file 3: — S3. Essential dataset for interstitial lung disease. [file 1750-1172-7-40-S3.doc]

**Supplemental file 3: essential dataset for interstitial lung diseases**

**Part one: diagnosis form**

- Diagnosis
  - Date of diagnosis, age at diagnosis
  - Date of onset
  - Associated diagnosis
- Basis of the diagnosis
  - Thoracic CT-scan (date, result)
  - Respiratory functional tests and blood gas
  - Lung biopsy
  - Immune tests
  - BAL
  - Genetics (patient and family): *SFTPB*, *SFTPC*, *SFTPA*, *ABCA3*, *TITF1*, others

**Part two: annual follow-up form**

- Medical examination
  - Weight, high, respiratory rate, thoracic deformation, oxygen rate on air or oxygen requirement, number of exacerbations, others
- Surgical events
  - Lung biopsy, others
- Tests
  - Chest radiography, CT-scan, arterial blood gas, respiratory functional tests, sleep monitoring, cardiac echography, others
- Treatment
  - Steroids (pulses / oral), immunosuppressive treatments, azithromycin, enteral feeding, oxygen therapy, physiotherapy, non invasive ventilation, others
- Outcome
  - On-going follow up / move to another centre, another country (precise) / lost to follow up / deceased (date, reason for death) /
