# Supplementary material for: A national internet-linked based database for pediatric interstitial lung diseases: the French network
Source: Orphanet J Rare Dis. 2012 Jun 15;7:40. doi: 10.1186/1750-1172-7-40 (PMC3458912; doi:10.1186/1750-1172-7-40)
Supplement: Additional file 4: — S4. Extensive interstitial lung disease (ILD) database dictionary (in French). Common dataset to the 4 groups of diagnosis (interstitial lung diseases, respiratory malformations, ciliary dyskinesia, and other rare diseases with chronic respiratory insufficiency) are highlighted in yellow. [file 1750-1172-7-40-S4.doc]

**Supplemental file 4: extensive interstitial lung disease (ILD) database dictionary (in French).**

Common dataset to the 4 groups of diagnosis (interstitial lung diseases, respiratory malformations, ciliary dyskinesia, and other rare diseases with chronic respiratory insufficiency) are highlighted in yellow.

Legend:

| Needed data |
| --- |
| Common dataset |

| **Table** | **Libelle** | **Valeur** |
| --- | --- | --- |
| anapath | identifiant de anapath |  |
| anapath | ID_fiche_clinique | int(10) unsigned |
| anapath | **Vaisseaux** |  |
| anapath | **Prélèvement pulmonaire** | enum('Non renseigné','Oui','Non') |
| anapath | Autre(s) tissu(s) prélevé(s) | longtext |
| anapath | Date de l'examen | date |
| anapath | **Nom du médecin** | varchar(50) |
| anapath | Type | enum('Non renseigné','Biopsie chirurgicale','Biopsie transbronchique','Pièce opératoire','Autopsie') |
| anapath | Volume | enum('Non renseigné','< 1 cm','[ 1 - 5 ] cm','> 5 cm') |
| anapath | Poids (g) | varchar(4) |
| anapath | Congélation | enum('Non renseigné','Oui','Non') |
| anapath | Microbiologie | enum('Non renseigné','Oui','Non') |
| anapath | *Précisez* | longtext |
| anapath | Fixation formol | enum('Non renseigné','Oui','Autre') |
| anapath | *Si autre, précisez* | varchar(50) |
| anapath | Cytologie (appositions) | enum('Non renseigné','Oui','Non') |
| anapath | *Précisez* | longtext |
| anapath | Microscopie électronique | enum('Non renseigné','Oui','Non') |
| anapath | *Précisez* | longtext |
| anapath | Pathologie pulmonaire préalable | enum('Non renseigné','Oui','Non') |
| anapath | Siège de la biopsie | set('Lobe supérieur','Lobe moyen','Lobe inférieur') |
| anapath | Lobe | set('Droit','Gauche') |
| anapath | **Bloc** | enum('Non renseigné','Oui','Non') |
| anapath | Intensité (ABCA3) | enum('Non renseigné','0','+','++','+++') |
| anapath | Localisation (ABCA3) | set('Granulaire','Membranaire','Cytoplasmique','Autre') |
| anapath | Commentaire (ABCA3) | varchar(50) |
| anapath | Intensité (TTF1) | enum('Non renseigné','0','+','++','+++') |
| anapath | Localisation (TTF1) | set('Granulaire','Membranaire','Cytoplasmique','Autre') |
| anapath | Commentaire (TTF1) | varchar(50) |
| anapath | **Biopsie** | enum('Non renseigné','Contributif','Non contributif','Autre') |
| anapath | **Diagnostic définitif** | varchar(50) |
| anapath | Commentaire(s) | longtext |
| anapath | Conclusion pour le résumé | longtext |
| anapath | Nombre de blocs | varchar(50) |
| anapath | N° de référence | varchar(50) |
| anapath | Lames | enum('Non renseigné','Oui','Non') |
| anapath | Colorations | enum('Non renseigné','Oui','Non') |
| anapath | *Type de coloration* | set('HES','Autre') |
| anapath | *Si autre, préciser* | varchar(50) |
| anapath | Immunohistochimie | enum('Non renseigné','Oui','Non') |
| anapath | *Anticorps étudiés* | set('EMA','SP-B','pro-SPC','ABC A3','TTF 1','Autre') |
| anapath | *Si autre, préciser* | longtext |
| anapath | Intensité (EMA) | enum('Non renseigné','0','+','++','+++') |
| anapath | Localisation (EMA) | set('Granulaire','Membranaire','Cytoplasmique','Autre') |
| anapath | Commentaire (EMA) | varchar(50) |
| anapath | Intensité (SP-B) | enum('Non renseigné','0','+','++','+++') |
| anapath | Localisation (SP-B) | set('Granulaire','Membranaire','Cytoplasmique','Autre') |
| anapath | Commentaire (SP-B) | varchar(50) |
| anapath | Intensité (pro-SPC) | enum('Non renseigné','0','+','++','+++') |
| anapath | Localisation (pro-SPC) | set('Granulaire','Membranaire','Cytoplasmique','Autre') |
| anapath | Commentaire (pro-SPC) | varchar(50) |
| anapath | **Inflammation** | enum('Non renseigné','Absente','Présente') |
| anapath | Si présente, précisez | varchar(50) |
| anapath | **Voies aériennes - Bronches membraneuses** | enum('Non renseigné','Libres','Rétrecies','Inflammatoires') |
| anapath | **Bronchiole terminale et au-delà** | enum('Non renseigné','Libre','Inflammatoire','Végétation','Nodule') |
| anapath | Vaisseaux artériels | enum('Non renseigné','Normaux','Pathologiques') |
| anapath | Vaisseaux veineux | enum('Non renseigné','Normaux','Pathologiques') |
| anapath | Vaisseaux lymphatiques | enum('Non renseigné','Normaux','Pathologiques') |
| anapath | **Plèvre** | enum('Non renseigné','Normale','Inflammatoire') |
| anapath | Plèvre septa-perilobulaire | enum('Non renseigné','Normale','Epaississement') |
| anapath | **Divers** | longtext |
| anapath | **Lumière alvéolaire** | enum('Non renseigné','Libre','Cellules','Substance','Microkyste') |
| anapath | **Images JPEG** | enum('Non renseigné','Oui','Non') |
| anapath | Image 1 | varchar(50) |
| anapath | Image 2 | varchar(50) |
| anapath | Image 3 | varchar(50) |
| anapath | Image 4 | varchar(50) |
| anapath | Image 5 | varchar(50) |
| anapath | Image 6 | varchar(50) |
| anapath | Image 7 | varchar(50) |
| anapath | Image 8 | varchar(50) |
| anapath | Image 9 | varchar(50) |
| anapath | Image 10 | varchar(50) |
| anapath | *Si cellules, type* | set('Macrophages','Neutrophiles','Eosinophiles','Pneumocytes','Lymphocytes','Siderophages') |
| anapath | *Si substance, type* | enum('Non renseigné','Protéinose','Cholestérol') |
| anapath | **Bordure alvéolaire** | enum('Non renseigné','Normale','Métaplasique') |
| anapath | **Interstitium alvéolaire** | enum('Non renseigné','Normal','Anormal') |
| anapath |  | set('Cellules','Capillaires') |
| anapath | *Si cellules, type* | set('Macrophages','Polynucléaires','Fibroblastes','Lymphocytes dispersés','Lymphocytes en nodules') |
| anapath | **Interstitium peribronchiolaire** | enum('Non renseigné','Normal','Cellules','Capillaires','Lymphatiques') |
| anapath | *Si lymphatique* | set('Dilaté','Musclé','HMB 45') |
| anapath | Si cellules, type | set('Macrophages','Polynucléaires','Fibroblastes','Lymphocytes dispersés','Lymphocytes en nodules') |
| anapath | Nom du ou des pathologiste(s) | varchar(50) |
| anapath | Nom de l'hôpital où l'analyse est effectuée | varchar(50) |
| anapath | Ville | varchar(50) |
| anapath | **Lecture** | enum('Non renseigné','Première','Relecture') |
| antecedent_diag | **Maladies chroniques dans la famille** | |
| antecedent_diag | **Autres antécédents familiaux** | longtext |
| antecedent_diag | **Maladies respiratoires chroniques autres que l'asthme** | enum('Non renseigné','Oui','Non') |
| antecedent_diag | *Si oui, préciser* | set('Père','Mère','Fratrie','Grand-père paternel','Grand-père maternel','Grand-mère paternelle','Grand-mère maternelle') |
| antecedent_diag | **Asthme** | enum('Non renseigné','Oui','Non') |
| antecedent_diag | *Si oui, préciser* | set('Père','Mère','Fratrie') |
| antecedent_diag | **Allergies** | enum('Non renseigné','Oui','Non') |
| antecedent_diag | *Si oui, préciser* | set('Père','Mère','Fratrie') |
| antecedent_diag | **Détresse respiratoire néonatale** | enum('Non renseigné','Oui','Non') |
| antecedent_diag | *Si oui, préciser* | set('Père','Mère','Fratrie','Grand-père paternel','Grand-père maternel','Grand-mère paternelle','Grand-mère maternelle') |
| antecedent_diag | **Décès néonatal inexpliqué** | enum('Non renseigné','Oui','Non') |
| antecedent_diag | *Si oui, préciser* | set('Fratrie','Cousin paternel','Cousin maternel','Oncle paternel','Oncle maternel','Tante paternelle','Tante maternelle') |
| antecedent_diag | *Si autre(s) virus, précisez* | longtext |
| antecedent_diag | *Si sérotype, précisez* | longtext |
| antecedent_diag | Si virus retrouvé(s), préciser | set('Avant 1 an','Entre 1 et 18 ans','Après 18 ans') |
| antecedent_diag | Si entre 1 et 18 ans, préciser | enum('Non renseigné','1','2','3','4','5','6','7','8','9','10','11','12','13','14','15','16','17','18') |
| antecedent_diag | Commentaire | longtext |
| antecedent_diag | **Mycoplasme** | enum('Non renseigné','Non','Suspecté','Documenté') |
| antecedent_diag | Si documenté, méthode | set('Sérologie','PCR') |
| antecedent_diag | Date de début | date |
| antecedent_diag | Age | varchar(50) |
| antecedent_diag | **Chlamydiae** | enum('Non renseigné','Non','Suspecté','Documenté') |
| antecedent_diag | Si documenté, méthode | set('Sérologie','PCR','Autre') |
| antecedent_diag | Si documentée | enum('Non renseigné','Pneumoniae','Trachomatis','Psittaci') |
| antecedent_diag | Date de début | date |
| antecedent_diag | Age | varchar(50) |
| antecedent_diag | **Pneumocystis jiroveci** | enum('Non renseigné','Non','Suspecté','Documenté') |
| antecedent_diag | Si documenté, méthode | set('PCR','Coloration (GROCOTT)','Autre') |
| antecedent_diag | Date de début | date |
| antecedent_diag | Age | varchar(50) |
| antecedent_diag | **Infection bactérienne** | enum('Non renseigné','Non','Suspectée','Documentée') |
| antecedent_diag | Nombre | varchar(50) |
| antecedent_diag | Date à la 1ère infection | date |
| antecedent_diag | Age à la 1ère infection | varchar(50) |
| antecedent_diag | *Si documentée, précisez* | longtext |
| antecedent_diag | **Autre(s) germe(s)** | longtext |
| antecedent_diag | **Autres antécédents pulmonaires** | longtext |
| antecedent_diag | **Hémoptysie** | enum('Non renseigné','Oui','Non') |
| antecedent_diag | Si oui, date de début | date |
| antecedent_diag | Age | varchar(50) |
| antecedent_diag | *Nombre d'épisodes* | enum('Non renseigné','1','2','3','4','5','6','7','8','9','10') |
| antecedent_diag | **Pneumothorax** | enum('Non renseigné','Oui','Non') |
| antecedent_diag | Si oui, date de début | date |
| antecedent_diag | Age | varchar(50) |
| antecedent_diag | *Nombre d'épisodes* | enum('Non renseigné','1','2','3','4','5','6','7','8','9','10') |
| antecedent_diag | **Infections** |  |
| antecedent_diag | **Virus** | enum('Non renseigné','Non','Suspecté','Documenté') |
| antecedent_diag | Virus retrouvé | set('Adenovirus','CMV','EBV','Influenzae','Parainfluenzae','Rougeole','VIH','VRS','Autre') |
| antecedent_diag | **Troubles de déglutition** | enum('Non renseigné','Oui','Non') |
| antecedent_diag | Si oui, date de début | date |
| antecedent_diag | Age | varchar(50) |
| antecedent_diag | **Autres antécédents digestifs** | longtext |
| antecedent_diag | **Reflux gastro-oesophagien** | enum('Non renseigné','Oui','Non') |
| antecedent_diag | Si oui, date de début | date |
| antecedent_diag | Age | varchar(50) |
| antecedent_diag | **Atteinte hépatique** | enum('Non renseigné','Oui','Non') |
| antecedent_diag | Si oui, date de début | date |
| antecedent_diag | Si oui, type d'atteinte | set('Cholestase','Cirrhose','Cytolyse','Hypertension portale') |
| antecedent_diag | **Diarrhée chronique** | enum('Non renseigné','Oui','Non') |
| antecedent_diag | **Nutrition entérale au long cours** | enum('Non renseigné','Oui','Non') |
| antecedent_diag | Si oui, méthode | enum('Non renseigné','Par sonde','Gastrostomie') |
| antecedent_diag | Date de mise en route | date |
| antecedent_diag | Age de début | varchar(50) |
| antecedent_diag | **Antécédents médicaux** | longtext |
| antecedent_diag | **Antécédents chirurgicaux** | longtext |
| antecedent_diag | Antécédents majeurs | longtext |
| antecedent_diag | **Autres antécédents** | longtext |
| antecedent_diag | Appendicectomie | varchar(4) |
| antecedent_diag | ID_antecedent_diag |  |
| antecedent_diag | ID_patient | int(10) unsigned |
| antecedent_diag | **Date de mise à jour** | date |
| antecedent_diag | Grossesse | set('Unique','Multiple','Induite') |
| antecedent_diag | Anomalies échographiques | enum('Non renseigné','Oui','Non') |
| antecedent_diag | *Si oui, préciser* | varchar(50) |
| antecedent_diag | Liquide amniotique | enum('Non renseigné','Clair','Teinté','Méconial') |
| antecedent_diag | Infection materno-foetale | enum('Non renseigné','Non','Suspectée','Confirmée') |
| antecedent_diag | **Détresse respiratoire** | enum('Non renseigné','Oui','Non') |
| antecedent_diag | Intubation | enum('Non renseigné','Oui','Non') |
| antecedent_diag | Si oui, moment | enum('Non renseigné','Avant la 1ère heure de vie','Après la 1ère heure de vie') |
| antecedent_diag | Si après la 1ère heure de vie, à quelle heure | varchar(50) |
| antecedent_diag | Durée | varchar(50) |
| antecedent_diag |  | enum('Non renseigné','Minute(s)','Heure(s)','Jour(s)') |
| antecedent_diag | Type de ventilation | set('Ventilation conventionnelle','OHF','AREC','ECMO') |
| antecedent_diag | Ventilation non invasive | enum('Non renseigné','Oui','Non') |
| antecedent_diag | Durée | varchar(50) |
| antecedent_diag |  | enum('Non renseigné','Heure(s)','Jour(s)') |
| antecedent_diag | Oxygénothérapie | enum('Non renseigné','Oui','Non') |
| antecedent_diag | Durée | varchar(50) |
| antecedent_diag |  | enum('Non renseigné','Heure(s)','Jour(s)') |
| antecedent_diag | Diagnostic anténatal de la maladie interstitielle | enum('Non renseigné','Oui','Non') |
| antecedent_diag | **Instillation de surfactant** | enum('Non renseigné','Oui','Non') |
| antecedent_diag | Nombre d'instillations | enum('Non renseigné','1','2','3','4','5','6','7','8','9','10') |
| antecedent_diag | **Corticothérapie systémique post-natale** | enum('Non renseigné','Oui','Non') |
| antecedent_diag | Age de début (en jours) | varchar(50) |
| antecedent_diag | Durée (en jours) | varchar(50) |
| antecedent_diag | Corticothérapie maternelle prénatale | enum('Non renseigné','Oui','Non') |
| antecedent_diag | Accouchement | enum('Non renseigné','Voie basse','Césarienne') |
| antecedent_diag | Terme (semaines d'aménorrhée) | varchar(255) |
| antecedent_diag | Poids de naissance (g) | varchar(255) |
| antecedent_diag | Taille à la naissance (cm) | varchar(255) |
| antecedent_diag | Périmètre crnien (cm) | varchar(255) |
| antecedent_diag | Score APGAR à 1' | enum('Non renseigné','0','1','2','3','4','5','6','7','8','9','10') |
| antecedent_diag | Score APGAR à 5' | enum('Non renseigné','0','1','2','3','4','5','6','7','8','9','10') |
| antecedent_diag | Score APGAR à 10' | enum('Non renseigné','0','1','2','3','4','5','6','7','8','9','10') |
| antecedent_diag | Souffrance foetale aiguë | enum('Non renseigné','Oui','Non') |
| antecedent_diag | **Tabagisme** | enum('Non renseigné','Non','Actif','Passif') |
| antecedent_diag | *Si passif, préciser* | set('Père','Mère','Fratrie','Conjoint','Nourrice') |
| antecedent_diag | *Si actif, nombre de paquets/année* | varchar(50) |
| antecedent_diag | **Addictions** | varchar(50) |
| antecedent_diag | **Animaux** | enum('Non renseigné','Oui','Non') |
| antecedent_diag | Si oui, lesquels | set('Chat','Chien','Cheval','Oiseau','Rongeur','Autre') |
| antecedent_diag | *Si autre, précisez* | varchar(50) |
| antecedent_diag | Depuis quand (si plusieurs dates, les séparer par un point-virgule) | varchar(50) |
| antecedent_diag | **Autres facteurs environnementaux** | longtext |
| antecedent_diag | **Patient ayant des frères et s**œurs | enum('Non renseigné','Oui','Non') |
| antecedent_diag | Si oui, nombre | varchar(50) |
| antecedent_diag | **Commentaires** | longtext |
| antecedent_diag | **Frères et sœurs** |  |
| antecedent_diag | Autres personnes atteintes d'une pathologie interstitielle dans la famille | enum('Non renseigné','Oui','Non') |
| antecedent_diag | Nombre de personnes atteintes d'une pathologie interstitielle dans la famille | varchar(50) |
| antecedent_diag | **Patient ayant des enfants** | enum('Non renseigné','Oui','Non') |
| antecedent_diag | Si oui, nombre | varchar(50) |
| antecedent_diag | **Enfants** |  |
| autres_organes | identifiant de autres_organes |  |
| autres_organes | ID_fiche_clinique | int(10) unsigned |
| autres_organes | Hépatomégalie | enum('Non renseigné','Oui','Non') |
| autres_organes | Déformation articulaire | enum('Non renseigné','Oui','Non') |
| autres_organes | Ictère | enum('Non renseigné','Oui','Non') |
| autres_organes | Aphte | enum('Non renseigné','Oui','Non') |
| autres_organes | Douleur osseuse | enum('Non renseigné','Oui','Non') |
| autres_organes | Hypertension portale | enum('Non renseigné','Oui','Non') |
| autres_organes | Oedeme maculaire | enum('Non renseigné','Oui','Non') |
| autres_organes | Splénomégalie | enum('Non renseigné','Oui','Non') |
| autres_organes | Oedeme papillaire | enum('Non renseigné','Oui','Non') |
| autres_organes | Epanchement articulaire | enum('Non renseigné','Oui','Non') |
| autres_organes | Hémorragie rétinienne | enum('Non renseigné','Oui','Non') |
| autres_organes | Néovascularisation rétinienne | enum('Non renseigné','Oui','Non') |
| autres_organes | Malformation vertébrale | enum('Non renseigné','Oui','Non') |
| autres_organes | Macule | enum('Non renseigné','Oui','Non') |
| autres_organes | Myalgie | enum('Non renseigné','Oui','Non') |
| autres_organes | Pustule | enum('Non renseigné','Oui','Non') |
| autres_organes | Papule | enum('Non renseigné','Oui','Non') |
| autres_organes | Ostéolyse | enum('Non renseigné','Oui','Non') |
| autres_organes | Tubercule | enum('Non renseigné','Oui','Non') |
| autres_organes | Nodule | enum('Non renseigné','Oui','Non') |
| autres_organes | Ostéoporose | enum('Non renseigné','Oui','Non') |
| autres_organes | Sclérodermie | enum('Non renseigné','Oui','Non') |
| autres_organes | Synovite | enum('Non renseigné','Oui','Non') |
| autres_organes | Polymyosite | enum('Non renseigné','Oui','Non') |
| autres_organes | Erythème | enum('Non renseigné','Oui','Non') |
| autres_organes | Dysphagie | enum('Non renseigné','Oui','Non') |
| autres_organes | Gingivite | enum('Non renseigné','Oui','Non') |
| autres_organes | Achalasie | enum('Non renseigné','Oui','Non') |
| autres_organes | Granulome | enum('Non renseigné','Oui','Non') |
| autres_organes | Episclérite | enum('Non renseigné','Oui','Non') |
| autres_organes | Lupus pernio | enum('Non renseigné','Oui','Non') |
| autres_organes | Plaques psoriasiformes | enum('Non renseigné','Oui','Non') |
| autres_organes | Trouble de la motricité oesophagienne | enum('Non renseigné','Oui','Non') |
| autres_organes | Anomalie de courbure du rachis | enum('Non renseigné','Oui','Non') |
| autres_organes | Exophtalmie | enum('Non renseigné','Oui','Non') |
| autres_organes | Arthralgie inflammatoire | enum('Non renseigné','Oui','Non') |
| autres_organes | Glaucome | enum('Non renseigné','Oui','Non') |
| autres_organes | Arthralgie mécanique | enum('Non renseigné','Oui','Non') |
| autres_organes | Ascite | enum('Non renseigné','Oui','Non') |
| autres_organes | Vascularite leucocytoclasique | enum('Non renseigné','Oui','Non') |
| autres_organes | Arthrite monoarticulaire | enum('Non renseigné','Oui','Non') |
| autres_organes | Malabsorption | enum('Non renseigné','Oui','Non') |
| autres_organes | Arthrite polyarticulaire | enum('Non renseigné','Oui','Non') |
| autres_organes | Sténose du canal lacrymal | enum('Non renseigné','Oui','Non') |
| autres_organes | Maladie inflammatoire du tube digestif | enum('Non renseigné','Oui','Non') |
| autres_organes | Déficit force musculaire | enum('Non renseigné','Oui','Non') |
| autres_organes | Elargissement glandes lacrymales | enum('Non renseigné','Oui','Non') |
| autres_organes | Obstruction glandes lacrymales | enum('Non renseigné','Oui','Non') |
| autres_organes | **Atteinte cardiaque** | enum('Non renseigné','Oui','Non') |
| autres_organes | Cardiomyopathie | enum('Non renseigné','Oui','Non') |
| autres_organes | Embolie pulmonaire | enum('Non renseigné','Oui','Non') |
| autres_organes | Thrombose veineuse | enum('Non renseigné','Oui','Non') |
| autres_organes | Thrombose artérielle | enum('Non renseigné','Oui','Non') |
| autres_organes | HTA | enum('Non renseigné','Oui','Non') |
| autres_organes | HTAP | enum('Non renseigné','Oui','Non') |
| autres_organes | Anomalie de perfusion myocardique (microcirculation) | enum('Non renseigné','Oui','Non') |
| autres_organes | **Autres atteintes** | longtext |
| autres_organes | **Commentaires** | longtext |
| autres_organes | Souffle cardiaque | enum('Non renseigné','Oui','Non') |
| autres_organes | Orthopnée | enum('Non renseigné','Oui','Non') |
| autres_organes | Insuffisance valvulaire | enum('Non renseigné','Oui','Non') |
| autres_organes | Insuffisance cardiaque | enum('Non renseigné','Oui','Non') |
| autres_organes | Trouble du rythme | enum('Non renseigné','Oui','Non') |
| autres_organes | Trouble de conduction | enum('Non renseigné','Oui','Non') |
| autres_organes | Péricardite | enum('Non renseigné','Oui','Non') |
| autres_organes | Myocardite | enum('Non renseigné','Oui','Non') |
| autres_organes | Endocardite | enum('Non renseigné','Oui','Non') |
| autres_organes | Coronarite | enum('Non renseigné','Oui','Non') |
| autres_organes | **Atteinte ORL** | enum('Non renseigné','Oui','Non') |
| autres_organes | Polypes nasaux | enum('Non renseigné','Oui','Non') |
| autres_organes | Obstruction nasale | enum('Non renseigné','Oui','Non') |
| autres_organes | Epistaxis | enum('Non renseigné','Oui','Non') |
| autres_organes | Rhinorrhée séro-sanglante | enum('Non renseigné','Oui','Non') |
| autres_organes | Rhinite croûteuse | enum('Non renseigné','Oui','Non') |
| autres_organes | Ulcérations nasales | enum('Non renseigné','Oui','Non') |
| autres_organes | Déformation en selle du cartilage nasal | enum('Non renseigné','Oui','Non') |
| autres_organes | Anosmie | enum('Non renseigné','Oui','Non') |
| autres_organes | Ulcération du voile du palais | enum('Non renseigné','Oui','Non') |
| autres_organes | Anomalie des glandes salivaires | enum('Non renseigné','Oui','Non') |
| autres_organes | Parotidite | enum('Non renseigné','Oui','Non') |
| autres_organes | Otite séromuqueuse | enum('Non renseigné','Oui','Non') |
| autres_organes | Otorrhée | enum('Non renseigné','Oui','Non') |
| autres_organes | Hypoacousie | enum('Non renseigné','Oui','Non') |
| autres_organes | Surdité | enum('Non renseigné','Oui','Non') |
| autres_organes | Cholestéatome | enum('Non renseigné','Oui','Non') |
| autres_organes | **Autres atteintes** | longtext |
| autres_organes | **Commentaires** | longtext |
| autres_organes | Dyspnée laryngée | enum('Non renseigné','Oui','Non') |
| autres_organes | Dysphonie | enum('Non renseigné','Oui','Non') |
| autres_organes | Stridor | enum('Non renseigné','Oui','Non') |
| autres_organes | Sténose laryngée | enum('Non renseigné','Oui','Non') |
| autres_organes | Ulcération laryngée | enum('Non renseigné','Oui','Non') |
| autres_organes | Cordes vocales | enum('Non renseigné','Normales','Anormales') |
| autres_organes | Si anormales, précisez | longtext |
| autres_organes | Sinusite | enum('Non renseigné','Oui','Non') |
| autres_organes | **Atteinte neurologique** | enum('Non renseigné','Oui','Non') |
| autres_organes | Parésie diaphragmatique | enum('Non renseigné','Oui','Non') |
| autres_organes | Névralgie | enum('Non renseigné','Oui','Non') |
| autres_organes | Hypotonie | enum('Non renseigné','Oui','Non') |
| autres_organes | Retard mental | enum('Non renseigné','Oui','Non') |
| autres_organes | Régression cognitive | enum('Non renseigné','Oui','Non') |
| autres_organes | Troubles du comportement | enum('Non renseigné','Oui','Non') |
| autres_organes | **Autres atteintes** | longtext |
| autres_organes | **Commentaires** | longtext |
| autres_organes | Neuropathie | enum('Non renseigné','Oui','Non') |
| autres_organes | Atteinte des nerfs crniens | enum('Non renseigné','Oui','Non') |
| autres_organes | Ataxie | enum('Non renseigné','Oui','Non') |
| autres_organes | Syndrome Pyramidal | enum('Non renseigné','Oui','Non') |
| autres_organes | Syndrome Extrapyramidal | enum('Non renseigné','Oui','Non') |
| autres_organes | Epilepsie | enum('Non renseigné','Oui','Non') |
| autres_organes | Méningite | enum('Non renseigné','Oui','Non') |
| autres_organes | **Atteinte dermatologique** | enum('Non renseigné','Oui','Non') |
| autres_organes | Calcinose | enum('Non renseigné','Oui','Non') |
| autres_organes | Erythème noueux | enum('Non renseigné','Oui','Non') |
| autres_organes | Icthyose | enum('Non renseigné','Oui','Non') |
| autres_organes | Lésions papulonodulaires | enum('Non renseigné','Oui','Non') |
| autres_organes | Panniculite | enum('Non renseigné','Oui','Non') |
| autres_organes | Prurit | enum('Non renseigné','Oui','Non') |
| autres_organes | Purpura | enum('Non renseigné','Oui','Non') |
| autres_organes | Raynaud | enum('Non renseigné','Oui','Non') |
| autres_organes | Sclérose cutanée | enum('Non renseigné','Oui','Non') |
| autres_organes | Acrocyanose | enum('Non renseigné','Oui','Non') |
| autres_organes | Télangiectasie | enum('Non renseigné','Oui','Non') |
| autres_organes | **Autres atteintes** | longtext |
| autres_organes | **Commentaires** | longtext |
| autres_organes | Alopécie | enum('Non renseigné','Oui','Non') |
| autres_organes | Angiome | enum('Non renseigné','Oui','Non') |
| autres_organes | Anomalies tissu de soutien | enum('Non renseigné','Oui','Non') |
| autres_organes | Atrophie cutanée | enum('Non renseigné','Oui','Non') |
| autres_organes | Atteintes muqueuses | enum('Non renseigné','Oui','Non') |
| autres_organes | **Atteinte osseuse** | enum('Non renseigné','Oui','Non') |
| autres_organes | Commentaire(s) | longtext |
| autres_organes | **Autres atteintes** | longtext |
| autres_organes | **Commentaires** | longtext |
| autres_organes | Atteinte articulaire | enum('Non renseigné','Oui','Non') |
| autres_organes | Commentaire(s) | longtext |
| autres_organes | Atteinte musculaire | enum('Non renseigné','Oui','Non') |
| autres_organes | Commentaire(s) | longtext |
| autres_organes | Atteinte osseuse | enum('Non renseigné','Oui','Non') |
| autres_organes | Commentaire(s) | longtext |
| autres_organes | Atteinte tendineuse | enum('Non renseigné','Oui','Non') |
| autres_organes | **Atteinte ophtalmo** | enum('Non renseigné','Oui','Non') |
| autres_organes | **Autres atteintes** | longtext |
| autres_organes | **Commentaires** | longtext |
| autres_organes | Rétinite pigmentaire | enum('Non renseigné','Oui','Non') |
| autres_organes | Conjonctivite | enum('Non renseigné','Oui','Non') |
| autres_organes | Kératite | enum('Non renseigné','Oui','Non') |
| autres_organes | Uvéite antérieure | enum('Non renseigné','Oui','Non') |
| autres_organes | Uvéite postérieure | enum('Non renseigné','Oui','Non') |
| autres_organes | Choriorétinite | enum('Non renseigné','Oui','Non') |
| autres_organes | Névrite optique | enum('Non renseigné','Oui','Non') |
| autres_organes | Syndrome sec | enum('Non renseigné','Oui','Non') |
| autres_organes | Atteinte des glandes lacrymales | enum('Non renseigné','Oui','Non') |
| autres_organes | **Atteinte néphrologique** | enum('Non renseigné','Oui','Non') |
| autres_organes | Hypertension artérielle | enum('Non renseigné','Oui','Non') |
| autres_organes | Glomérulopathie à complexes immuns circulants | enum('Non renseigné','Oui','Non') |
| autres_organes | **Autres atteintes** | longtext |
| autres_organes | **Commentaires** | longtext |
| autres_organes | Insuffisance rénale | enum('Non renseigné','Oui','Non') |
| autres_organes | Syndrome néphrotique | enum('Non renseigné','Oui','Non') |
| autres_organes | Syndrome néphritique | enum('Non renseigné','Oui','Non') |
| autres_organes | Protéinurie | enum('Non renseigné','Oui','Non') |
| autres_organes | Hématurie | enum('Non renseigné','Oui','Non') |
| autres_organes | Glomérulonéphrite | enum('Non renseigné','Oui','Non') |
| autres_organes | Néphrite interstitielle | enum('Non renseigné','Oui','Non') |
| autres_organes | Tubulopathie proximale / Dysfonctionnement du tubule proximal | enum('Non renseigné','Oui','Non') |
| autres_organes | Tubulopathie distale / Dysfonctionnement du tubule distal | enum('Non renseigné','Oui','Non') |
| autres_organes | **Atteinte hématologique** | enum('Non renseigné','Oui','Non') |
| autres_organes | Trouble de la coagulation | enum('Non renseigné','Oui','Non') |
| autres_organes | **Autres atteintes** | longtext |
| autres_organes | **Commentaires** | longtext |
| autres_organes | Anémie ferriprive | enum('Non renseigné','Oui','Non') |
| autres_organes | Anémie hémolytique | enum('Non renseigné','Oui','Non') |
| autres_organes | Leucopénie | enum('Non renseigné','Oui','Non') |
| autres_organes | Lymphopénie | enum('Non renseigné','Oui','Non') |
| autres_organes | Thrombopénie centrale | enum('Non renseigné','Oui','Non') |
| autres_organes | Thrombopénie périphérique | enum('Non renseigné','Oui','Non') |
| autres_organes | Hyperéosinophilie | enum('Non renseigné','Oui','Non') |
| autres_organes | Polyglobulie | enum('Non renseigné','Oui','Non') |
| autres_organes | Thrombocytose | enum('Non renseigné','Oui','Non') |
| autres_organes | Sydrome myéloprolifératif | enum('Non renseigné','Oui','Non') |
| autres_organes | Syndrome lymphoprolifératif | enum('Non renseigné','Oui','Non') |
| autres_organes | Déficit immunitaire humoral | enum('Non renseigné','Oui','Non') |
| autres_organes | Déficit immunitaire cellulaire | enum('Non renseigné','Oui','Non') |
| autres_organes | **Atteinte endocrinologique** | enum('Non renseigné','Oui','Non') |
| autres_organes | Si oui, précisez | longtext |
| autres_organes | **Autres atteintes** | longtext |
| autres_organes | **Commentaires** | longtext |
| autres_organes | Pubère | enum('Non renseigné','Oui','Non') |
| autres_organes | Diabète | enum('Non renseigné','Oui','Non') |
| autres_organes | Si oui, précisez | enum('Non renseigné','Type I','Type II') |
| autres_organes | Intolérance glucidique | enum('Non renseigné','Oui','Non') |
| autres_organes | Hypothyroïdie | enum('Non renseigné','Oui','Non') |
| autres_organes | Hyperthyroïdie | enum('Non renseigné','Oui','Non') |
| autres_organes | Insuffisance surrénale | enum('Non renseigné','Oui','Non') |
| autres_organes | Diabète insipide | enum('Non renseigné','Oui','Non') |
| autres_organes | Maladie métabolique | enum('Non renseigné','Oui','Non') |
| autres_organes | **Atteinte hépatique/gastrologique** | enum('Non renseigné','Oui','Non') |
| autres_organes | Cirrhose | enum('Non renseigné','Oui','Non') |
| autres_organes | **Autres atteintes** | longtext |
| autres_organes | **Commentaires** | longtext |
| autres_organes | Dénutrition | enum('Non renseigné','Oui','Non') |
| autres_organes | Diarrhée chronique | enum('Non renseigné','Oui','Non') |
| autres_organes | Trouble de déglutition | enum('Non renseigné','Oui','Non') |
| autres_organes | RGO | enum('Non renseigné','Oui','Non') |
| autres_organes | Hémorragie digestive | enum('Non renseigné','Oui','Non') |
| autres_organes | Insuffisance hépatocellulaire | enum('Non renseigné','Oui','Non') |
| autres_organes | Cholestase | enum('Non renseigné','Oui','Non') |
| autres_organes | Date de l'examen | date |
| autres_organes_explo | identifiant de autres_organes_explo | |
| autres_organes_explo | ID_fiche_clinique | int(10) unsigned |
| autres_organes_explo | Date | date |
| autres_organes_explo | **Coeur gauche** | enum('Non renseigné','Exploré','Non exploré') |
| autres_organes_explo | Index de masse myocardiaque (g/m2) | varchar(50) |
| autres_organes_explo | **Coeur droit** | enum('Non renseigné','Exploré','Non exploré') |
| autres_organes_explo | Diamètre télédiastolique du VD (mm) | varchar(5) |
| autres_organes_explo | Dilatation du VD | enum('Non renseigné','Oui','Non') |
| autres_organes_explo | Diamètre VCI en expiration (mm) | varchar(5) |
| autres_organes_explo | Diamètre VCI en inspiration (mm) | varchar(5) |
| autres_organes_explo | Insuffisance tricuspide (m/s) | varchar(5) |
| autres_organes_explo | **Flux pulmonaire en doppler pulsé** | enum('Non renseigné','Mesuré','Non mesuré') |
| autres_organes_explo | Vitesse maximale (m/s) | varchar(5) |
| autres_organes_explo | Temps d'accélération du flux pulmonaire (msec) | varchar(5) |
| autres_organes_explo | **Flux d'insuffisance pulmonaire en doppler continu** | enum('Non renseigné','Mesuré','Non mesuré') |
| autres_organes_explo | **Echographie cardiaque** | enum('Non renseigné','Oui','Non') |
| autres_organes_explo | A la date du | date |
| autres_organes_explo | Commentaire | longtext |
| autres_organes_explo | Pression télésystolique VD (gradient OD - VD + 5 ou 10 mm Hg) | varchar(5) |
| autres_organes_explo | Pression pulmonaire diastolique (mmHg) | varchar(5) |
| autres_organes_explo | Pression pulmonaire moyenne (mmHg) | varchar(5) |
| autres_organes_explo | Commentaire | longtext |
| autres_organes_explo | **Epreuve d'effort** | enum('Non renseigné','Oui','Non') |
| autres_organes_explo | Date | date |
| autres_organes_explo | Commentaire | longtext |
| autres_organes_explo | Diamètre télédiastolique VG (mm) | varchar(5) |
| autres_organes_explo | Diamètre télésystolique VG (mm) | varchar(5) |
| autres_organes_explo | Fraction de raccourcissement (%) | varchar(5) |
| autres_organes_explo | **Cathétérisme cardiaque** | enum('Non renseigné','Oui','Non') |
| autres_organes_explo | Date | date |
| autres_organes_explo | PAP moyenne | varchar(50) |
| autres_organes_explo | PAP diastolique | varchar(50) |
| autres_organes_explo | Résistance | varchar(50) |
| autres_organes_explo | Réactivité | enum('Non renseigné','Oui','Non') |
| autres_organes_explo | Commentaire | longtext |
| autres_organes_explo | Septum interventriculaire en TD(mm) | varchar(5) |
| autres_organes_explo | Paroi postérieure en TD (mm) | varchar(5) |
| autres_organes_explo | Flux mitral | varchar(5) |
| autres_organes_explo | Rapport E/A | varchar(5) |
| autres_organes_explo | Temps de relaxation isovolumétrique | varchar(5) |
| autres_organes_explo | Temps de décélération (ms) | varchar(50) |
| autres_organes_explo | **Atteinte valvulaire** | enum('Non renseigné','Oui','Non') |
| autres_organes_explo | *Si oui, préciser* | set('Valve mitrale','Valve aortique','Valve tricuspide','Valve pulmonaire') |
| autres_organes_explo | **Biopsie** | enum('Non renseigné','Fait','Non fait') |
| autres_organes_explo | Date | date |
| autres_organes_explo | **Immunologie** | enum('Non renseigné','Fait','Non fait') |
| autres_organes_explo | **Autre(s) exploration(s)** | longtext |
| autres_organes_explo | **Commentaires** | longtext |
| autres_organes_explo | **Test au Synacthène®** | enum('Non renseigné','Fait','Non fait') |
| autres_organes_explo | Date | date |
| autres_organes_explo | **Ostéodensitométrie** | enum('Non renseigné','Fait','Non fait') |
| autres_organes_explo | Date | date |
| autres_organes_explo | **Autre(s) exploration(s)** | longtext |
| autres_organes_explo | **Commentaires** | longtext |
| autres_organes_explo | **Biologie** | enum('Non renseigné','Fait','Non fait') |
| autres_organes_explo | **Myélogramme** | enum('Non renseigné','Fait','Non fait') |
| autres_organes_explo | Date | date |
| autres_organes_explo | **Echographie abdominale** | enum('Non renseigné','Oui','Non') |
| autres_organes_explo | Date | date |
| autres_organes_explo | **EMG de la déglutition** | enum('Non renseigné','Fait','Non fait') |
| autres_organes_explo | Date | date |
| autres_organes_explo | Commentaire EMG | longtext |
| autres_organes_explo | **Radiocinéma de la déglutition** | enum('Non renseigné','Fait','Non fait') |
| autres_organes_explo | Date | date |
| autres_organes_explo | Commentaire radiocinéma de la déglutition | longtext |
| autres_organes_explo | **TOGD** | enum('Non renseigné','Normal','Engagement hiatal','Béance cardiale') |
| autres_organes_explo | Date | date |
| autres_organes_explo | Si autre, préciser | varchar(50) |
| autres_organes_explo | Commentaire TOGD | longtext |
| autres_organes_explo | **pHmétrie** | enum('Non renseigné','Oui','Non') |
| autres_organes_explo | Date | date |
| autres_organes_explo | Vésicule | enum('Non renseigné','Non vue','Normale','Lithiasique') |
| autres_organes_explo | Antécédent de cholécystectomie | varchar(4) |
| autres_organes_explo | Taille (diamètre sagittal maximal en cm) | varchar(5) |
| autres_organes_explo | Durée d'enregistrement | varchar(5) |
| autres_organes_explo | Pourcentage de temps avec un pH < 4 | varchar(5) |
| autres_organes_explo | Commentaire pHmétrie | longtext |
| autres_organes_explo | **Cholangiographie par IRM** | enum('Non renseigné','Oui','Non') |
| autres_organes_explo | Date | date |
| autres_organes_explo | Commentaire cholangiographie | longtext |
| autres_organes_explo | **Fibroscopie digestive** | enum('Non renseigné','Oui','Non') |
| autres_organes_explo | Date | date |
| autres_organes_explo | Oesophage | enum('Non renseigné','Normale','Grade I','Grade II','Grade III','Grade IV') |
| autres_organes_explo | Varices oesophagiennes | enum('Non renseigné','Stade I','Stade II','Stade III') |
| autres_organes_explo | Estomac | enum('Non renseigné','Normal','Hernie hiatale','Béance cardiale','Malposition cardiotubérositaire','Gastrite') |
| autres_organes_explo | Duodénum | enum('Non renseigné','Normal','Ulcère duodénal') |
| autres_organes_explo | Commentaire fibroscopie digestive | longtext |
| autres_organes_explo | **Manométrie** | enum('Non renseigné','Oui','Non') |
| autres_organes_explo | Si oui, précisez | longtext |
| autres_organes_explo | **Intolérance au gluten** | enum('Non renseigné','Fait','Non fait') |
| autres_organes_explo | Date | date |
| autres_organes_explo | Documentée | enum('Non renseigné','Oui','Non') |
| autres_organes_explo | Examens réalisés | longtext |
| autres_organes_explo | **Autre(s) exploration(s)** | longtext |
| autres_organes_explo | Foie | enum('Non renseigné','Normal','Hyperéchogène','Nodulaire','Scléroatrophique','Stéatose') |
| autres_organes_explo | Signes d'hypertension portale | varchar(4) |
| autres_organes_explo | Flèche hépatique (cm) | varchar(5) |
| autres_organes_explo | Rate | enum('Non renseigné','Normal','Ptosée','Hypertrophique') |
| autres_organes_explo | Flèche (cm) | varchar(5) |
| autres_organes_explo | Intestins | enum('Non renseigné','Épaisseur de la paroi normale','Épaisseur de la paroi augmentée') |
| autres_organes_explo | Mucocèle appendiculaire | varchar(5) |
| autres_organes_explo | Reins - Lithiase | enum('Non renseigné','Oui','Non') |
| autres_organes_explo | Autres | varchar(100) |
| autres_organes_explo | Commentaire échographie abdominale | longtext |
| autres_organes_explo | **Biochimie** | enum('Non renseigné','Fait','Non fait') |
| autres_organes_explo | **Echographie rénale** | enum('Non renseigné','Fait','Non fait') |
| autres_organes_explo | Date | date |
| autres_organes_explo | **Autre(s) exploration(s)** | longtext |
| autres_organes_explo | **Commentaires** | longtext |
| autres_organes_explo | **Scanner cérébral** | enum('Non renseigné','Fait','Non fait') |
| autres_organes_explo | Date | date |
| autres_organes_explo | **IRM** | enum('Non renseigné','Fait','Non fait') |
| autres_organes_explo | Date | date |
| autres_organes_explo | **Autre(s) exploration(s)** | longtext |
| autres_organes_explo | **Commentaires** | longtext |
| autres_organes_explo | **Acuité visuelle** | enum('Non renseigné','Fait','Non fait') |
| autres_organes_explo | Date | date |
| autres_organes_explo | **Electrorétinogramme** | enum('Non renseigné','Fait','Non fait') |
| autres_organes_explo | Date | date |
| autres_organes_explo | **Potentiels évoqués visuels** | enum('Non renseigné','Fait','Non fait') |
| autres_organes_explo | Date | date |
| autres_organes_explo | **Fond d'oeil** | enum('Non renseigné','Fait','Non fait') |
| autres_organes_explo | Date | date |
| autres_organes_explo | **Examen à la lampe à fente** | enum('Non renseigné','Fait','Non fait') |
| autres_organes_explo | Date | date |
| autres_organes_explo | **Vision des couleurs** | enum('Non renseigné','Fait','Non fait') |
| autres_organes_explo | Date | date |
| autres_organes_explo | **Nasofibroscopie** | enum('Non renseigné','Fait','Non fait') |
| autres_organes_explo | Date | date |
| autres_organes_explo | **Autre(s) exploration(s)** | longtext |
| autres_organes_explo | **Commentaires** | longtext |
| autres_organes_explo | **Audiométrie** | enum('Non renseigné','Fait','Non fait') |
| autres_organes_explo | Date | date |
| autres_organes_explo | **Scanner des sinus** | enum('Non renseigné','Fait','Non fait') |
| autres_organes_explo | Date | date |
| autres_organes_explo | **PEA** | enum('Non renseigné','Fait','Non fait') |
| autres_organes_explo | Date | date |
| autres_organes_explo | **Autres examens** | longtext |
| autres_organes_explo | **Absorptiométrie** | enum('Non renseigné','Fait','Non fait') |
| autres_organes_explo | Masse grasse (médiane pour l'ge) | varchar(5) |
| autres_organes_explo | Masse maigre (médiane pour l'ge) | varchar(10) |
| autres_organes_explo | **Densité minérale osseuse (Z(t))** | enum('Non renseigné','Fait','Non fait') |
| autres_organes_explo | A la date du | date |
| autres_organes_explo | Score rachis | varchar(2) |
| autres_organes_explo | Score hanche | varchar(2) |
| autres_organes_explo | **Age osseux** | varchar(20) |
| autres_organes_explo | A la date du | date |
| biologie | ID_biologie |  |
| biologie | ID_fiche_clinique | int(10) unsigned |
| biologie | Autres | longtext |
| biologie | Na+ (mmol/l) | varchar(50) |
| biologie | K+ (mmol/l) | varchar(50) |
| biologie | Bicarbonates (mmol/l) | varchar(50) |
| biologie | Cl- (mmol/l) | varchar(50) |
| biologie | Bilirubine conjuguée (µmol/l) | varchar(50) |
| biologie | Bilirubine totale (µmol/l) | varchar(50) |
| biologie | ASAT-TGO (UI/l) | varchar(50) |
| biologie | ALAT-TGP (UI/l) | varchar(50) |
| biologie | GammaGT (UI/l) | varchar(50) |
| biologie | Amylase (UI/l) | varchar(50) |
| biologie | Lipase (UI/l) | varchar(50) |
| biologie | Préalbumine (mg/l) | varchar(50) |
| biologie | Albumine (g/l) | varchar(50) |
| biologie | Beta2 microalbumine (mg/l) | varchar(50) |
| biologie | Fer (µmol/l) | varchar(50) |
| biologie | Ferritine (µg/l) | varchar(50) |
| biologie | Coefficient de saturation | varchar(50) |
| biologie | Transferrine (g/l) | varchar(50) |
| biologie | Urée (mmol/l) | varchar(50) |
| biologie | Acide urique (mmol/l) | varchar(50) |
| biologie | Créatinine (µmol/l) | varchar(50) |
| biologie | Glycémie à jeun (mmol/l) | varchar(50) |
| biologie | Glucose (mmol/l) | varchar(50) |
| biologie | Protides (g/l) | varchar(50) |
| biologie | Trou anionique (mmol/l) | varchar(50) |
| biologie | CRP (mg/l) | varchar(50) |
| biologie | Calcium (mmol/l) | varchar(50) |
| biologie | Phosphore (mmol/l) | varchar(50) |
| biologie | Autres | longtext |
| biologie | **Test de la sueur** | enum('Non renseigné','Fait','Non fait') |
| biologie | Date du test | date |
| biologie | Vitamine A (µg/l) | varchar(50) |
| biologie | Vitamine E (mg/l) | varchar(50) |
| biologie | Vitamine D1-25 OH (ng/ml) | varchar(50) |
| biologie | Vitamine D3-25 OH (ng/ml) | varchar(50) |
| biologie | Vitamine K (ng/ml) | varchar(50) |
| biologie | Retinol Binding Protein (mg/l) | varchar(50) |
| biologie | Parathormone (ng/l) | varchar(50) |
| biologie | Ostéocalcine (µg/l) | varchar(50) |
| biologie | Cholestérol total (mmol/l) | varchar(50) |
| biologie | HDL (mmol/l) | varchar(50) |
| biologie | LDL (mmol/l) | varchar(50) |
| biologie | Triglycérides (mmol/l) | varchar(50) |
| biologie | Méthode utilisée | enum('Non renseigné','Gibson','Conductivité','Exsudose','Potentiométrie') |
| biologie | Lpa (mg/l) | varchar(50) |
| biologie | Apo A1 (g/l) | varchar(50) |
| biologie | Apo B (g/l) | varchar(50) |
| biologie | Glycémie moyenne à jêun (mmol/l) | varchar(50) |
| biologie | Glycémie post prandiale (mmol/l) | varchar(50) |
| biologie | Hémoglobine 1C glycosylée (%) | varchar(50) |
| biologie | C peptide (µg/l) | varchar(50) |
| biologie | HGPO |  |
| biologie | Glycémie à T0 | varchar(50) |
| biologie | Glycémie à T120 | varchar(50) |
| biologie |  | enum('Non renseigné','Normale','Intolérance glucidique','Diabète') |
| biologie | IGF1 (µg/l) | varchar(50) |
| biologie | IGFBP2 (µg/l) | varchar(50) |
| biologie | IGFBP3 (µg/l) | varchar(50) |
| biologie | GH (µg/l) | varchar(50) |
| biologie | T3 (µg/l) | varchar(50) |
| biologie | T4 (µg/l) | varchar(50) |
| biologie | TSH (µU/ml) | varchar(50) |
| biologie | Cortisol (µg/100ml) | varchar(50) |
| biologie | 1ère mesure (mmol/ l Cl) | varchar(50) |
| biologie | D pyridoline / Créate | varchar(50) |
| biologie | Crosslab | varchar(50) |
| biologie | 2ème mesure (mmol/ l Cl) | varchar(50) |
| biologie | Résultat (mmol/ l Cl) | varchar(50) |
| biologie | ECA (UI/l) | varchar(50) |
| biologie | Lysozyme (mg/l) | varchar(50) |
| biologie | **NFS normale** | enum('Non renseigné','Oui','Non') |
| biologie | Autres | longtext |
| biologie | Fibrinogène (g/l) | varchar(50) |
| biologie | Hématies (10^12/l) | varchar(50) |
| biologie | Hémoglobine (g/dl) | varchar(50) |
| biologie | Hématocrite (%) | varchar(50) |
| biologie | Plaquettes (10^9/l) | varchar(50) |
| biologie | Leucocytes (10^9/l) | varchar(50) |
| biologie | Neutrophiles (%) | varchar(50) |
| biologie | Eosinophiles (%) | varchar(50) |
| biologie | Basophiles (%) | varchar(50) |
| biologie | Lymphocytes (%) | varchar(50) |
| biologie | Monocytes (%) | varchar(50) |
| biologie | TP (%) | varchar(50) |
| biologie | INR | varchar(50) |
| biologie | TCA (Patient/Témoin) | varchar(50) |
| biologie | Facteur II (%) | varchar(50) |
| biologie | Facteur V (%) | varchar(50) |
| biologie | Facteur VII + X (%) | varchar(50) |
| biologie | Autres | longtext |
| biologie | Na+ (mmol/l) | varchar(50) |
| biologie | K+ (mmol/l) | varchar(50) |
| biologie | Microalbumine (mg/l) | varchar(50) |
| biologie | Alpha1 microglobuline (mg/l) | varchar(50) |
| biologie | Urée (mmol/l) | varchar(50) |
| biologie | Créatinine (µmol/l) | varchar(50) |
| biologie | Protides (g/l) | varchar(50) |
| biologie | Glucose (mmol/l) | varchar(50) |
| biologie | Osmolarité (mmol/l) | varchar(50) |
| biologie | Calcium (mmol/l) | varchar(50) |
| biologie | Phosphore (mmol/l) | varchar(50) |
| biologie | Date de l'examen | date |
| biologie | Date de la prescription | date |
| bolus | identifiant de bolus |  |
| bolus | ID_fiche_clinique | int(10) unsigned |
| bolus | **Corticothérapie interbolus** | enum('Non renseigné','Oui','Non') |
| bolus | **Hydrocortisone** | enum('Non renseigné','Oui','Non') |
| bolus | Quantité (mg/kg) | varchar(50) |
| bolus | Quantité (mg) | varchar(50) |
| bolus | Equivalent prednisone | varchar(50) |
| bolus | Nutrition entérale | varchar(50) |
| bolus | PaO2 (mmHg) : Valeur mesurée | varchar(50) |
| bolus | PaCO2 (mmHg) : Valeur mesurée | varchar(50) |
| bolus | pH : Valeur mesurée | varchar(50) |
| bolus | Corticothérapie interbolus (mg) | varchar(50) |
| bolus | **Prednisone** | enum('Non renseigné','Oui','Non') |
| bolus | Quantité (mg/kg) | varchar(50) |
| bolus | Quantité (mg) | varchar(50) |
| bolus | **Prednisolone** | enum('Non renseigné','Oui','Non') |
| bolus | Quantité (mg/kg) | varchar(50) |
| bolus | Quantité (mg) | varchar(50) |
| bolus | Equivalent prednisone | varchar(50) |
| bolus | Traitements associés | longtext |
| bolus | **Betaméthasone** | enum('Non renseigné','Oui','Non') |
| bolus | Quantité (mg/kg) | varchar(50) |
| bolus | Quantité (mg) | varchar(50) |
| bolus | Equivalent prednisone | varchar(50) |
| bolus | **N° du bolus** | int(50) |
| bolus | Date de début | date |
| bolus | Surface corporelle (m²) | varchar(50) |
| bolus | **Administration de bolus de Méthylprednisolone** | |
| bolus | Posologie (mg/jour) | varchar(50) |
| bolus | Posologie (mg/m²/jour) | varchar(50) |
| bolus | Administration | enum('Non renseigné','Par jour','Sur 2 jours','Sur 3 jours') |
| bolus | Soluté utilisé | enum('Non renseigné','G 5%','NaCl 0.9%') |
| bolus | Durée de la perfusion / J1 | varchar(50) |
| bolus | Durée de la perfusion / J2 | varchar(50) |
| bolus | Durée de la perfusion / J3 | varchar(50) |
| bolus | Tolérance | enum('Non renseigné','Bonne','Mauvaise') |
| bolus | *Si mauvaise* | set('Hypertension','Hyperglycémie','Glycosurie') |
| bolus | EFR | enum('Non renseigné','Fait','Non fait') |
| bolus | Gaz du sang | enum('Non renseigné','Fait','Non fait') |
| bolus | TDM | enum('Non renseigné','Fait','Non fait') |
| bolus | Echographie cardiaque | enum('Non renseigné','Fait','Non fait') |
| carnet_d_adresses | Poste | int(10) unsigned |
| carnet_d_adresses | identificateur du carnet d'adresses | |
| carnet_d_adresses | Titre | enum('Dr.','Pr.','Mme','Mlle','M.') |
| carnet_d_adresses | Nom | varchar(20) |
| carnet_d_adresses | Prénom | varchar(20) |
| carnet_d_adresses | Spécialité | int(10) unsigned |
| carnet_d_adresses | Btiment | varchar(50) |
| carnet_d_adresses | Numéro | varchar(50) |
| carnet_d_adresses | Rue | varchar(60) |
| carnet_d_adresses | Code postal | varchar(20) |
| carnet_d_adresses | Ville | varchar(60) |
| carnet_d_adresses | Pays | int(11) |
| carnet_d_adresses | Si autre pays, préciser | varchar(50) |
| carnet_d_adresses | e-mail | varchar(50) |
| carnet_d_adresses | Téléphone du travail | varchar(20) |
| carnet_d_adresses | Numéro de fax | varchar(20) |
| carnet_d_adresses | Téléphone portable | varchar(20) |
| carnet_d_adresses | Téléphone du domicile | varchar(20) |
| carnet_d_adresses | Hôpital | varchar(60) |
| carnet_d_adresses | Service | varchar(80) |
| diagnostic | identifiant de diagnostic |  |
| diagnostic | ID_patient | int(10) unsigned |
| diagnostic | Date du diagnostic | date |
| diagnostic | Age au diagnostic de pathologie interstitielle | varchar(50) |
| diagnostic | Si PI liée à une maladie granulomateuse, préciser | enum('Non renseigné','Sarcoïdose','Autre') |
| diagnostic | *Si autre, préciser* | varchar(50) |
| diagnostic | Si PI liée à une maladie métabolique, préciser | enum('Non renseigné','Maladie lysosomiale','Hypercalcémie avec hypocalciurie familiale','Autre') |
| diagnostic | *Si autre, préciser* | varchar(50) |
| diagnostic | Si PI liée à l'atteinte d'autres organes, préciser | enum('Non renseigné','Maladie de Crohn','Maladie coeliaque','Cirrhose biliaire primitive','Hépatite chronique','Sclérose tubéreuse de Bourneville','Neurofibromatose','Ataxie-télangiectasie','Amyloïdose','Autre') |
| diagnostic | *Si autre, préciser* | varchar(50) |
| diagnostic | Si PI spécifique à l'enfant, préciser | enum('Non renseigné','Hyperplasie des cellules neuro-endocrines','Pneumonie chronique de l''enfant','Autre') |
| diagnostic | *Si autre, préciser* | varchar(50) |
| diagnostic | **Commentaire sur le diagnostic** | longtext |
| diagnostic | Histoire de la maladie | longtext |
| diagnostic | Date de la 1ère consultation | date |
| diagnostic | Age à la 1ère consultation | varchar(50) |
| diagnostic | **Le diagnostic a été suggéré par** | set('antécédents familiaux','retard de croissance','dénutrition','problème respiratoire','autre') |
| diagnostic | *Si autre, préciser* | varchar(50) |
| diagnostic | **Diagnostic** | enum('Non renseigné','Fait','Non fait','Non étiqueté') |
| diagnostic |  | set('Pneumopathie interstitielle d''exposition','PI liée à une connectivite','PI liée à une vascularite','PI liée à une maladie granulomateuse','PI liée à une maladie métabolique','Histiocytose langheransienne','Autre PI liée à l''atteinte d''autres organ |
| diagnostic | *Si autre, préciser* | varchar(50) |
| diagnostic | Si pneumopathie interstitielle d'exposition, préciser | enum('Non renseigné','Pneumopathie d''hypersensibilité','Pneumopathie toxique (médicamenteuse, radique tabagique)','Autre') |
| diagnostic | *Si autre, préciser* | varchar(50) |
| diagnostic | Si PI liée à une connectivite, préciser | enum('Non renseigné','Arthrite rhumatoïde','Sclérose systémique','Lupus érythémateux aigu disséminé','Syndrome de Sjögren','Dermatomyosite et polymyosite','Spondylarthrite ankylosante','Connectivite mixte','Autre') |
| diagnostic | *Si autre, préciser* | varchar(50) |
| diagnostic | Si PI liée à une vascularite, préciser | enum('Non renseigné','Vascularite pulmonaire à ANCA (granulomatose de Wegener, syndrome de Churg et Strauss, polyangéite microscopique)','Maladie des Ac anti membrane basale glomérulaire','Purpura rhumatoïde','Cryoglobulinémie','Autre') |
| diagnostic | *Si autre, préciser* | varchar(50) |
| dietetique | identifiant de dietetique |  |
| dietetique | ID_fiche_clinique | int(10) unsigned |
| dietetique | **Date de la consultation diététique** | date |
| dietetique | **Noms des diététiciens** | varchar(50) |
| dietetique | Téléphone | varchar(50) |
| dietetique | Résumé de la précédente prise en charge | longtext |
| dietetique | Motif de la prise en charge | longtext |
| dietetique | Résultat de l'évaluation alimentaire | longtext |
| dietetique | Diagnostic diététique | longtext |
| dietetique | Objectifs diététiques | longtext |
| dietetique | Conseils donnés | longtext |
| dietetique | **Supplément calorique** | enum('Non renseigné','Oui','Non') |
| dietetique | Suppléments à faire prescrire | longtext |
| dietetique | **Nutrition entérale** | enum('Non renseigné','Oui','Non') |
| dietetique | *Si oui, précisez* | enum('Non renseigné','Sonde nasogastrique','Gastrostomie') |
| dietetique | Besoin calorique (Kcal/j) | varchar(50) |
| dietetique | Nb de calories apportées par NEDC /j | varchar(50) |
| dietetique | Commentaires | longtext |
| dietetique | Produit | varchar(50) |
| dietetique | Quantité | varchar(50) |
| dietetique | Durée moyenne | varchar(50) |
| dietetique | Poids | varchar(50) |
| dietetique | Taille | varchar(50) |
| dietetique | Age statural | varchar(50) |
| dietetique | Apport recommandé | varchar(200) |
| dietetique | Estimation du besoin | varchar(200) |
| donnees_sociales | identifiant de donnees_sociales | |
| donnees_sociales | clé étrangère patient | int(10) unsigned |
| donnees_sociales | Appendicectomie | varchar(4) |
| donnees_sociales | **Rhinite saisonnière** | enum('Non renseigné','Oui','Non') |
| donnees_sociales | Allocations perçues | set('Aucune','Allocation d''éducation de l''enfant handicapé','Allocation adulte handicapé','Allocation logement','Autre (préciser ci-dessous)') |
| donnees_sociales | N° de sécurité sociale | varchar(255) |
| donnees_sociales | Protocole de soin | enum('Non renseigné','100%','Autre') |
| donnees_sociales | *Si autre, préciser* | varchar(255) |
| donnees_sociales | Date de début | date |
| donnees_sociales | Date de fin | date |
| donnees_sociales | Autre | varchar(150) |
| donnees_sociales | Compléments | varchar(255) |
| efr | identifiant de efr |  |
| efr | clé étrangère vers fiche clinique | int(10) unsigned |
| efr | saturation_valeur_pourcentage | varchar(255) |
| efr | Sexe | enum('Fille','Garçon') |
| efr | Conditions de réalisation | enum('Non renseigné','Assis','Couché','Debout') |
| efr | Corset | enum('Non renseigné','Oui','Non') |
| efr | Interface | enum('Non renseigné','Embout buccal','Masque facial') |
| efr | Date des EFR | date |
| efr | VR/CPT : Valeur attendue | varchar(50) |
| efr | VR/CPT : Valeur mesurée | varchar(10) |
| efr | VR/CPT (%) | varchar(50) |
| efr | ClDyn (ml/cmH2O) : Valeur attendue | varchar(50) |
| efr | ClDyn (ml/cmH2O) : Valeur mesurée | varchar(10) |
| efr | ClDyn (%) | varchar(50) |
| efr | ClDyn/CRF (ml/cmH2O/l) : Valeur attendue | varchar(50) |
| efr | ClDyn/CRF (ml/cmH2O/l) : Valeur mesurée | varchar(50) |
| efr | ClDyn/CRF (%) | varchar(50) |
| efr | VmaxCRF (l/s) : Valeur attendue | varchar(50) |
| efr | VmaxCRF (l/s) : Valeur mesurée | varchar(10) |
| efr | VmaxCRF (%) | varchar(50) |
| efr | CRF helium/azote (l) : Valeur attendue | varchar(50) |
| efr | CRF helium/azote (l) : Valeur mesurée | varchar(10) |
| efr | CRF helium/azote (%) | varchar(50) |
| efr | CRFpl (l) : Valeur attendue | varchar(50) |
| efr | CRFpl (l) : Valeur mesurée | varchar(10) |
| efr | CRFpl (%) | varchar(50) |
| efr | CVL (l) : Valeur attendue | varchar(50) |
| efr | CVL (l) : Valeur mesurée | varchar(10) |
| efr | CVL (%) | varchar(50) |
| efr | CVF (l) : Valeur attendue | varchar(50) |
| efr | CVF (l) : Valeur mesurée | varchar(10) |
| efr | CVF (%) | varchar(50) |
| efr | VEMS (l) : Valeur attendue | varchar(50) |
| efr | VEMS (l) : Valeur mesurée | varchar(10) |
| efr | VEMS (%) | varchar(50) |
| efr | VEMS/CVF : Valeur attendue | varchar(50) |
| efr | VEMS/CVF : Valeur mesurée | varchar(50) |
| efr | VEMS/CVF (%) | varchar(50) |
| efr | VE 0,4 s (l) | varchar(50) |
| efr | VE 0,5 s (l) | varchar(50) |
| efr | VE 0,75 s (l) | varchar(50) |
| efr | VE x s (l) | varchar(50) |
| efr | Préciser x | varchar(50) |
| efr | DEP (l/s) : Valeur attendue | varchar(50) |
| efr | DEP (l/s) : Valeur mesurée | varchar(10) |
| efr | DEP (%) | varchar(50) |
| efr | DEM 25-75 (l/s) : Valeur attendue | varchar(50) |
| efr | DEM 25-75 (l/s) : Valeur mesurée | varchar(10) |
| efr | DEM 25-75 (%) | varchar(50) |
| efr | DEM 50 (l/s) : Valeur attendue | varchar(50) |
| efr | DEM 50 (l/s) : Valeur mesurée | varchar(10) |
| efr | DEM 50 (%) | varchar(50) |
| efr | **Age du patient** | varchar(30) |
| efr | **Classe d'ge du patient** | enum('Nourrisson (< 3 ans)','Enfant','Adolescent','Adulte') |
| efr | DEM 25 (l/s) : Valeur attendue | varchar(50) |
| efr | DEM 25 (l/s) : Valeur mesurée | varchar(10) |
| efr | DEM 25 (%) | varchar(50) |
| efr | **Résistances** |  |
| efr | RPT (cmH2O/l/s) : Valeur attendue | varchar(50) |
| efr | RPT (cmH2O/l/s) : Valeur mesurée | varchar(10) |
| efr | RPT (%) | varchar(50) |
| efr | Raw (kPa/l/s) : Valeur attendue | varchar(50) |
| efr | Raw (kPa/l/s) : Valeur mesurée | varchar(10) |
| efr | Raw (%) | varchar(50) |
| efr | sRaw (kPa/l/s) : Valeur attendue | varchar(50) |
| efr | sRaw (kPa/l/s) : Valeur mesurée | varchar(50) |
| efr | sRaw (%) | varchar(50) |
| efr | Rint (kPa/l/s) : Valeur attendue | varchar(50) |
| efr | Rint (kPa/l/s) : Valeur mesurée | varchar(50) |
| efr | Rint (%) | varchar(50) |
| efr | ROF (kPa/l/s) : Valeur attendue | varchar(50) |
| efr | ROF (kPa/l/s) : Valeur mesurée | varchar(50) |
| efr | ROF (%) | varchar(50) |
| efr | **Mesure de la diffusion** |  |
| efr | DLCO apnée (mmol/min/kPa) : Valeur attendue | varchar(50) |
| efr | DLCO apnée (mmol/min/kPa) : Valeur mesurée | varchar(50) |
| efr | DLCO apnée (%) | varchar(50) |
| efr | KCO (DLCO/VA) apnée (mmol/min/kPa/l) : Valeur attendue | varchar(50) |
| efr | KCO (DLCO/VA) apnée (mmol/min/kPa/l) : Valeur mesurée | varchar(50) |
| efr | KCO (DLCO/VA) apnée (%) | varchar(50) |
| efr | Poids (en kg) | varchar(255) |
| efr | Taille ou envergure (en cm) | varchar(255) |
| efr | DLCO réinspiration (mmol/min/kPa) : Valeur attendue | varchar(50) |
| efr | DLCO réinspiration (mmol/min/kPa) : Valeur mesurée | varchar(50) |
| efr | DLCO réinspiration (%) | varchar(50) |
| efr | KCO (DLCO/VA) réinspiration (mmol/min/kPa/l) : Valeur attendue | varchar(50) |
| efr | KCO (DLCO/VA) réinspiration (mmol/min/kPa/l) : Valeur mesurée | varchar(50) |
| efr | KCO (DLCO/VA) réinspiration (%) | varchar(50) |
| efr | TLCO (mmol/min/kPa) : Valeur attendue | varchar(50) |
| efr | TLCO (mmol/min/kPa) : Valeur mesurée | varchar(50) |
| efr | TLCO (%) | varchar(50) |
| efr | TLCO VA (mmol/min/kPa) : Valeur attendue | varchar(50) |
| efr | TLCO VA (mmol/min/kPa) : Valeur mesurée | varchar(50) |
| efr | TLCO VA (%) | varchar(50) |
| efr | **NO expiré** |  |
| efr | FeNO 0.05 l/s (ppb) | varchar(50) |
| efr | FeNO x l/s (ppb) | varchar(50) |
| efr | Préciser x | varchar(50) |
| efr | CalvNO (ppb) | varchar(50) |
| efr | J'awNO (nl/mn) | varchar(50) |
| efr | J'awNO (x/x) | varchar(50) |
| efr | Préciser x/x | varchar(50) |
| efr | NO nasal (ppb) | varchar(50) |
| efr | Débit du NO nasal (nL/mn) | varchar(50) |
| efr | **Test BD** | enum('Non renseigné','Fait','Non fait') |
| efr | **Commentaires** | longtext |
| efr | CPT (l) : Valeur attendue | varchar(50) |
| efr | CPT (l) : Valeur mesurée | varchar(10) |
| efr | CPT (%) | varchar(50) |
| efr | VR (l) : Valeur attendue | varchar(50) |
| efr | VR (l) : Valeur mesurée | varchar(10) |
| efr | VR (%) | varchar(50) |
| efr | **Date** | date |
| efr | Matériel utilisé | varchar(50) |
| efr | Lieu de réalisation | enum('Non renseigné','Hôpital','Domicile','Centre','Autre') |
| efr | *Si autre, précisez* | varchar(50) |
| efr | *Précisez le débit* | varchar(50) |
| efr | Précisez le ventilateur utilisé | varchar(50) |
| efr | Moment d'enregistrement | enum('Non renseigné','Sieste','Nuit') |
| efr | Index apnée+hypopnée | varchar(50) |
| efr | Efficacité du sommeil (%) | varchar(50) |
| efr | Index de désaturation (chute >3 %) | varchar(50) |
| efr | PCO2 | enum('Non renseigné','Transcutanée','Exhalée') |
| efr | Variation maximale entre éveil et sommeil (KPa ou mmHg) | varchar(50) |
| efr | Commentaires | longtext |
| efr | Temps de sommeil Non REM (mn) | varchar(50) |
| efr | Temps de sommeil Non REM (%) | varchar(50) |
| efr | Temps de sommeil REM (mn) | varchar(50) |
| efr | Temps de sommeil REM (%) | varchar(50) |
| efr | Index d'apnée | varchar(50) |
| efr | Index d'hypopnée | varchar(50) |
| efr | Index d'apnée centrale | varchar(50) |
| efr | Index d'apnée obstructive | varchar(50) |
| efr | Microréveils | enum('Non renseigné','Oui','Non') |
| efr | Si oui, index | varchar(50) |
| efr | Saturation moyenne (%) | varchar(50) |
| efr | Saturation minimale (%) | varchar(50) |
| efr | Temps passé à une saturation <90% (mn) | varchar(50) |
| efr | Temps passé à une saturation <90% (% du tps sommeil) | varchar(50) |
| efr | Temps passé à une saturation entre 90 et 92 (mn) | varchar(50) |
| efr | Temps passé à une saturation entre 90 et 92 (% du tps sommeil) | varchar(50) |
| efr | Temps passé à une saturation <85% (mn) | varchar(50) |
| efr | Temps passé à une saturation <85% (% du tps sommeil) | varchar(50) |
| efr | Fréquence cardiaque moyenne (batt/min) | varchar(50) |
| efr | Fréquence cardiaque maximale (batt/min) | varchar(50) |
| efr | Fréquence cardiaque minimale (batt/min) | varchar(50) |
| efr | PO2 moyenne eveil (mm Hg ou KPa) | varchar(50) |
| efr | PO2 maximale eveil (mm Hg ou KPa) | varchar(50) |
| efr | PO2 minimale eveil (mm Hg ou KPa) | varchar(50) |
| efr | PO2 moyenne sommeil (mm Hg ou KPa) | varchar(50) |
| efr | PO2 moyenne sommeil (mm Hg ou KPa) | varchar(50) |
| efr | PO2 minimale sommeil (mm Hg ou KPa) | varchar(50) |
| efr | PCO2 moyenne éveil (mm Hg ou KPa) | varchar(50) |
| efr | PCO2 maximale éveil (mm Hg ou KPa) | varchar(50) |
| efr | PCO2 minimale éveil (mm Hg ou KPa) | varchar(50) |
| efr | PCO2 moyenne sommeil (mm Hg ou KPa) | varchar(50) |
| efr | PCO2 maximale sommeil (mm Hg ou KPa) | varchar(50) |
| efr | PCO2 minimale sommeil (mm Hg ou KPa) | varchar(50) |
| efr | Temps passé avec PCO2 > 45 mmHg (mn) | varchar(50) |
| efr | Temps passé avec PCO2 > 45 mmHg (% du tps sommeil) | varchar(50) |
| efr | Temps passé avec PCO2 > 50 mmHg (mn) | varchar(50) |
| efr | Temps passé avec PCO2 > 50 mmHg (% du tps sommeil) | varchar(50) |
| efr | Conditions de réalisation | enum('Non renseigné','Ventilation spontanée','Ventilation mécanique') |
| efr | *Précisez* | enum('Non renseigné','Sous air','Sous O2') |
| efr | Durée enregistrement (mn) | varchar(50) |
| efr | Durée totale temps de sommeil (mn) | varchar(50) |
| efr | **Examen du gaz du sang effectué** | enum('Non renseigné','Repos','Marche de 6 minutes','Exercice') |
| efr | Si marche de 6 minutes, distance parcourue (m) | varchar(50) |
| efr | Si exercice, consommation VO2 max (%) | varchar(50) |
| efr | SaO2 (%) : Valeur attendue | varchar(10) |
| efr | SaO2 (%) : Valeur mesurée | varchar(10) |
| efr | PaO2 (mmHg) : Valeur attendue | varchar(10) |
| efr | PaO2 (mmHg) : Valeur mesurée | varchar(10) |
| efr | PaCO2 (mmHg) : Valeur attendue | varchar(10) |
| efr | PaCO2 (mmHg) : Valeur mesurée | varchar(10) |
| efr | pH : Valeur attendue | varchar(10) |
| efr | pH : Valeur mesurée | varchar(10) |
| efr | Débit expiratoir maximal à la toux (l/mn) | varchar(50) |
| efr | Pression nasale lors du reniflement (cm H2O) | varchar(50) |
| efr | Autre(s) exploration(s) des muscles respiratoires | longtext |
| efr | Pression expiratoire maximale (cm H2O) : valeur attendue | varchar(50) |
| efr | Pression expiratoire maximale (cm H2O) : valeur mesurée | varchar(50) |
| efr | Pression expiratoire maximale (%) | varchar(50) |
| efr | Pression inspiratoire maximale (cm H2O) : valeur attendue | varchar(50) |
| efr | Pression inspiratoire maximale (cm H2O) : valeur mesurée | varchar(50) |
| efr | Pression inspiratoire maximale (%) | varchar(50) |
| efr | **Examen du gaz du sang effectué** | enum('Non renseigné','Air ambiant','O2') |
| efr | Si O2, débit (L/min) | varchar(50) |
| efr | Type GDS | enum('Non renseigné','Artériel','Veineux','Capillaire artérialisé') |
| efr | SaO2 (%) : Valeur attendue | varchar(50) |
| efr | SaO2 (%) : Valeur mesurée | varchar(10) |
| efr | PaO2 (mmHg) : Valeur attendue | varchar(50) |
| efr | PaO2 (mmHg) : Valeur mesurée | varchar(10) |
| efr | PaCO2 (mmHg) : Valeur attendue | varchar(50) |
| efr | PaCO2 (mmHg) : Valeur mesurée | varchar(10) |
| efr | pH : Valeur attendue | varchar(50) |
| efr | pH : Valeur mesurée | varchar(10) |
| efr | CPT (l) : Valeur attendue | varchar(50) |
| efr | CPT (l) : Valeur mesurée | varchar(50) |
| efr | CPT (%) : | varchar(50) |
| efr | % variation | varchar(50) |
| efr | CVL (l) : Valeur attendue | varchar(50) |
| efr | CVL (l) : Valeur mesurée | varchar(50) |
| efr | CVL (%) : | varchar(50) |
| efr | % variation | varchar(50) |
| efr | CVF (l) : Valeur attendue | varchar(50) |
| efr | CVF (l) : Valeur mesurée | varchar(50) |
| efr | CVF (%) : | varchar(50) |
| efr | % variation | varchar(50) |
| efr | VEMS (l) : Valeur attendue | varchar(50) |
| efr | VEMS (l) : Valeur mesurée | varchar(50) |
| efr | VEMS (%) : | varchar(50) |
| efr | % variation | varchar(50) |
| efr | VEMS/CVF : Valeur attendue | varchar(50) |
| efr | VEMS/CVF : Valeur mesurée | varchar(50) |
| efr | VEMS/CVF (%) : | varchar(50) |
| efr | % variation | varchar(50) |
| efr | DEM 25-75 (l/s) : Valeur attendue | varchar(50) |
| efr | DEM 25-75 (l/s) : Valeur mesurée | varchar(50) |
| efr | DEM 25-75 (%) : | varchar(50) |
| efr | % variation | varchar(50) |
| efr | DEM 50 (l/s) : Valeur attendue | varchar(50) |
| efr | DEM 50 (l/s) : Valeur mesurée | varchar(50) |
| efr | DEM 50 (%) : | varchar(50) |
| efr | % variation | varchar(50) |
| efr | DEM 25 (l/s) : Valeur attendue | varchar(50) |
| efr | DEM 25 (l/s) : Valeur mesurée | varchar(50) |
| efr | DEM 25 (%) : | varchar(50) |
| efr | % variation | varchar(50) |
| efr | VR (l) : Valeur attendue | varchar(50) |
| efr | VR (l) : Valeur mesurée | varchar(50) |
| efr | VR (%) : | varchar(50) |
| efr | % variation | varchar(50) |
| efr | Commentaires | longtext |
| efr | VR/CPT : Valeur attendue | varchar(50) |
| efr | VR/CPT : Valeur mesurée | varchar(50) |
| efr | VR/CPT (%) : | varchar(50) |
| efr | % variation | varchar(50) |
| efr | RVApl (kPa/l) : Valeur attendue | varchar(50) |
| efr | RVApl (kPa/l) : Valeur mesurée | varchar(50) |
| efr | RVApl (%) : | varchar(50) |
| efr | % variation | varchar(50) |
| efr | RVAo (kPa/l) : Valeur attendue | varchar(50) |
| efr | RVAo (kPa/l) : Valeur mesurée | varchar(50) |
| efr | RVAo (%) : | varchar(50) |
| efr | % variation | varchar(50) |
| efr | VmaxCRF (l/s) : Valeur attendue | varchar(50) |
| efr | VmaxCRF (l/s) : Valeur mesurée | varchar(50) |
| efr | VmaxCRF (%) : | varchar(50) |
| efr | % variation | varchar(50) |
| efr | CRF helium/azote (l) : Valeur attendue | varchar(50) |
| efr | CRF helium/azote (l) : Valeur mesurée | varchar(50) |
| efr | CRF helium/azote (%) : | varchar(50) |
| efr | % variation | varchar(50) |
| efr | CRFpl (l) : Valeur attendue | varchar(50) |
| efr | CRFpl (l) : Valeur mesurée | varchar(50) |
| efr | CRFpl (%) : | varchar(50) |
| efr | % variation | varchar(50) |
| endoscopie | identifiant de endoscopie |  |
| endoscopie | clé étrangère fiche clinique | int(10) unsigned |
| endoscopie | **Commentaires** | longtext |
| endoscopie | **Date de l'examen** | date |
| endoscopie | **Anesthésie générale** | enum('Non renseigné','Oui','Non') |
| endoscopie | Sécrétions | enum('Non renseigné','Oui','Non') |
| endoscopie | *Si oui, aspect* | varchar(50) |
| endoscopie | Orifices libres | enum('Non renseigné','Oui','Non') |
| endoscopie | *Si non, préciser* | varchar(50) |
| endoscopie | **Anomalies anatomiques** | enum('Non renseigné','Oui','Non') |
| endoscopie | *Si oui, préciser* | varchar(50) |
| endoscopie | **Larynx** | enum('Non renseigné','Exploré','Non exploré') |
| endoscopie | Inflammation | enum('Non renseigné','Oui','Non') |
| endoscopie | Dyskinésie | enum('Non renseigné','Oui','Non') |
| endoscopie | Sécrétions | enum('Non renseigné','Oui','Non') |
| endoscopie | *Si oui, aspect* | varchar(50) |
| endoscopie | **Trachée** | enum('Non renseigné','Exploré','Non exploré') |
| endoscopie | Inflammation | enum('Non renseigné','Oui','Non') |
| endoscopie | Dyskinésie | enum('Non renseigné','Oui','Non') |
| endoscopie | Sécrétions | enum('Non renseigné','Oui','Non') |
| endoscopie | *Si oui, aspect* | varchar(50) |
| endoscopie | **Bronches droites** | enum('Non renseigné','Exploré','Non exploré') |
| endoscopie | Inflammation | enum('Non renseigné','Oui','Non') |
| endoscopie | Dyskinésie | enum('Non renseigné','Oui','Non') |
| endoscopie | Sécrétions | enum('Non renseigné','Oui','Non') |
| endoscopie | *Si oui, aspect* | varchar(50) |
| endoscopie | Orifices libres | enum('Non renseigné','Oui','Non') |
| endoscopie | *Si non, préciser* | varchar(50) |
| endoscopie | **Bronches gauches** | enum('Non renseigné','Exploré','Non exploré') |
| endoscopie | Inflammation | enum('Non renseigné','Oui','Non') |
| endoscopie | Dyskinésie | enum('Non renseigné','Oui','Non') |
| endoscopie | Date de la fiche clinique | date |
| enfant | identifiant de enfant |  |
| enfant | clé étrangère vers patient | int(10) unsigned |
| enfant | Rang de cet enfant | varchar(50) |
| enfant | Sexe | enum('Non renseigné','Féminin','Masculin') |
| enfant | Atteint d'une pathologie interstitielle | enum('Non renseigné','Oui','Non') |
| enfant | Décédé d'une pathologie interstitielle | enum('Non renseigné','Oui','Non') |
| enfant | Référencé dans e-Pi | enum('Non renseigné','Oui','Non') |
| enfant | Lieu de suivi | varchar(50) |
| fiche_clinique | Type de fiche clinique | enum('Consultation','Consultation téléphonique','Hospitalisation de jour','Hospitalisation programmée','Hospitalisation d''urgence','Examen ou ordonnance hors visite') |
| fiche_clinique | Date de la fiche clinique | date |
| fiche_clinique | Si hospitalisation, du | date |
| fiche_clinique | au | date |
| fiche_clinique | Code séjour hôpital | varchar(255) |
| fiche_clinique | Médecin | int(10) unsigned |
| fiche_clinique | Responsable de salle | varchar(50) |
| fiche_clinique | Interne | varchar(50) |
| fiche_clinique | **Activité** | enum('Non renseigné','Normale','Modérément limitée','Limitée','Très limitée') |
| fiche_clinique | **Animaux** | enum('Non renseigné','Oui','Non') |
| fiche_clinique | Si oui, lesquels | set('Chat','Chien','Cheval','Oiseau','Rongeur','Autre') |
| fiche_clinique | *Si autre, précisez* | varchar(50) |
| fiche_clinique | Depuis quand (si plusieurs dates, les séparer par un point-virgule) | varchar(50) |
| fiche_clinique | **Autres facteurs environnementaux** | longtext |
| fiche_clinique | Nombre d'hospitalisations (hors centre) | varchar(50) |
| fiche_clinique | **Signes respiratoires** | set('Aucun','Symptômes non modifiés','Amélioration','Recrudescence de la toux','Aggravation de la gêne respiratoire (dyspnée)','Douleurs thoraciques','Hémoptysie','Pneumothorax') |
| fiche_clinique | **Signes digestifs** | set('Aucun','Douleurs abdominales','Diarrhée','Vomissements') |
| fiche_clinique | Situation professionnelle | enum('Non renseigné','Sans activité','Etudiant ou scolaire','Actif à temps plein','Actif à temps partiel') |
| fiche_clinique | Scolarité (Classe) ou activité professionnelle | varchar(50) |
| fiche_clinique | Sports habituellement pratiqués | varchar(100) |
| fiche_clinique | **Commentaire** | longtext |
| fiche_clinique | **Evènements chirurgicaux** | enum('Non renseigné','Oui','Non') |
| fiche_clinique | **Précisez le type de chirurgie** | set('Nissen','Pose chambre implantable','Gastrostomie','Autre') |
| fiche_clinique | Si nissen, date | date |
| fiche_clinique | Si pose d'une chambre implantable, date | date |
| fiche_clinique | Si gastrostomie, date | date |
| fiche_clinique | Si autre chirurgie, précisez | longtext |
| fiche_clinique | **Bolus à l'extérieur** | enum('Non renseigné','Oui','Non') |
| fiche_clinique | Si oui, nombre | varchar(50) |
| fiche_clinique | **Grossesse ou paternité** | enum('Non renseigné','Non applicable','Oui','Non') |
| fiche_clinique | **Contraception** | enum('Non renseigné','Oui','Non') |
| fiche_clinique | Si oui, date de début | date |
| fiche_clinique | Nature | varchar(50) |
| fiche_clinique | **Absentéisme scolaire ou professionnel** | enum('Non renseigné','Non applicable','Oui','Non') |
| fiche_clinique | Nombre de jours | varchar(4) |
| fiche_clinique | **Tabagisme** | enum('Non renseigné','Actif','Passif','Non') |
| fiche_clinique | *Si passif, préciser* | set('Père','Mère','Fratrie','Conjoint','Nourrice') |
| fiche_clinique | Si actif, nombre de paquets/année | varchar(50) |
| fiche_clinique | **Addictions** | varchar(50) |
| fiche_clinique | **Signes généraux** | set('Aucun','Asthénie','Fièvre','Anorexie','Activité diminuée') |
| fiche_clinique | **Toux** | set('Non renseigné','Absente','Occasionnelle','Quotidienne','Nocturne','Diurne') |
| fiche_clinique | **Dyspnée** | enum('Non renseigné','Absente','Si effort important','Si effort modéré','Au repos') |
| fiche_clinique | **Autre** | longtext |
| fiche_clinique | **Nombre d'hospitalisations** | varchar(5) |
| fiche_clinique | **Poids (Kg)** | varchar(50) |
| fiche_clinique | Poids attendu pour la taille (Kg) | varchar(10) |
| fiche_clinique | Poids attendu pour la taille (%) | varchar(4) |
| fiche_clinique | Evolution pondérale (g) | varchar(50) |
| fiche_clinique | **Pubère** | enum('Non renseigné','Oui','Non') |
| fiche_clinique | Si oui, date des premières règles | date |
| fiche_clinique | **Examen respiratoire** | enum('Non renseigné','Normal','Anormal') |
| fiche_clinique | **Dyspnée** | enum('Non renseigné','Oui','Non') |
| fiche_clinique | Classe d'ge | enum('Non renseigné','Nourrisson','Enfant') |
| fiche_clinique | Dyspnée | set('Au repos','A l''effort') |
| fiche_clinique | Stade | enum('Non renseigné','Stade 1','Stade 2','Stade 3','Stade 4') |
| fiche_clinique | Cyanose | enum('Non renseigné','Oui','Non') |
| fiche_clinique | **Taille (cm)** | varchar(50) |
| fiche_clinique | Taille attendue pour l'ge (cm) | varchar(10) |
| fiche_clinique | Taille attendue pour l'ge (%) | varchar(4) |
| fiche_clinique | Evolution staturale (cm) | varchar(50) |
| fiche_clinique | Hippocratisme digital | enum('Non renseigné','Oui','Non') |
| fiche_clinique | Déformation thoracique | enum('Non renseigné','Oui','Non') |
| fiche_clinique | Auscultation pulmonaire | enum('Non renseigné','Normale','Anormale') |
| fiche_clinique | Rles bronchiques | enum('Non renseigné','Localisés','Diffus') |
| fiche_clinique | Crépitants et sous-crépitants | enum('Non renseigné','Localisés','Diffus') |
| fiche_clinique | Sibilants | enum('Non renseigné','Localisés','Diffus') |
| fiche_clinique | Commentaires respiratoires | longtext |
| fiche_clinique | **Examen digestif** | enum('Non renseigné','Normal','Anormal') |
| fiche_clinique | Ballonnement abdominal | enum('Non renseigné','Oui','Non') |
| fiche_clinique | Hépatomégalie | enum('Non renseigné','Oui','Non') |
| fiche_clinique | Splénomégalie | enum('Non renseigné','Oui','Non') |
| fiche_clinique | DS Poids | varchar(255) |
| fiche_clinique | DS Taille | varchar(255) |
| fiche_clinique | Surface corporelle (m²) | varchar(50) |
| fiche_clinique | Commentaires digestifs | longtext |
| fiche_clinique | **Adénopathies** | enum('Non renseigné','Oui','Non') |
| fiche_clinique | *Si oui, préciser* | varchar(50) |
| fiche_clinique | **Atteinte d'autres organes** | enum('Non renseigné','Oui','Non') |
| fiche_clinique | **Autres** | longtext |
| fiche_clinique | **Commentaires** | longtext |
| fiche_clinique | **IMC - z score** | varchar(4) |
| fiche_clinique | IMC | varchar(50) |
| fiche_clinique | **Périmètre crnien (cm)** | varchar(50) |
| fiche_clinique | **Fréquence respiratoire (cycles/mn)** | varchar(255) |
| fiche_clinique | **Saturation O2** | set('Sous air','Sous O2') |
| fiche_clinique | SaO2 : sous air (%) | varchar(4) |
| fiche_clinique | Débit (l/mn) | varchar(50) |
| fiche_clinique | SaO2 : sous O2 (%) | varchar(50) |
| fiche_clinique | **Fréquence cardiaque (cycles/mn)** | varchar(255) |
| fiche_clinique | Tension artérielle systolique (mmHg) | varchar(6) |
| fiche_clinique | Tension artérielle diastolique (mmHg) | varchar(50) |
| fiche_clinique | **Tension artérielle moyenne (mmHg)** | varchar(50) |
| fiche_clinique | ID_fiche_clinique |  |
| fiche_clinique | ID patient | int(11) |
| fiche_clinique | CR envoyé | varchar(50) |
| fiche_clinique | Dyspnée à l'effort | enum('Non renseigné','Stade 1','Stade 2','Stade 3','Stade 4') |
| fiche_clinique |  | enum('Non renseigné','Heure(s)','Jour(s)') |
| fiche_clinique |  | enum('Non renseigné','Heure(s)','Jour(s)') |
| fiche_clinique |  | enum('Non renseigné','Heure(s)','Jour(s)') |
| fiche_clinique |  | enum('Non renseigné','Heure(s)','Jour(s)') |
| fiche_clinique | Température (°C) | varchar(255) |
| fiche_clinique | CR fait | varchar(50) |
| fiche_clinique | CR validé | varchar(50) |
|  | Synthèse |  |
| fiche_clinique | Respiratoire | set('Bilan','Thérapeutique','Pose CI','Décompensation respiratoire','Bilan prégreffe','Autre motif') |
| fiche_clinique | Autres | longtext |
| fiche_clinique |  | longtext |
| fiche_clinique |  | longtext |
| fiche_clinique | Bolus |  |
| fiche_clinique | LBA thérapeutique |  |
| fiche_clinique |  | longtext |
| fiche_clinique | **Actes chirurgicaux** | enum('Non renseigné','Oui','Non') |
| fiche_clinique | Type de chirurgie | set('Nissen','Pose chambre implantable','Gastrostomie','Autre') |
| fiche_clinique | Si autre chirurgie, précisez | varchar(50) |
| fiche_clinique |  | longtext |
| fiche_clinique | **Oxygénothérapie** | enum('Non renseigné','Oui','Non') |
| fiche_clinique | Posologie (mg/kg) | varchar(50) |
| fiche_clinique | Posologie (mg) | varchar(50) |
| fiche_clinique | Equivalent prednisone | varchar(50) |
| fiche_clinique | **Betaméthasone** | enum('Non renseigné','Oui','Non') |
| fiche_clinique | Posologie (mg/kg) | varchar(50) |
| fiche_clinique | Posologie (mg) | varchar(50) |
| fiche_clinique | Equivalent prednisone | varchar(50) |
| fiche_clinique | **Hydrocortisone** | enum('Non renseigné','Oui','Non') |
| fiche_clinique | Posologie (mg/kg) | varchar(50) |
| fiche_clinique | Posologie (mg) | varchar(50) |
| fiche_clinique | Equivalent prednisone | varchar(50) |
| fiche_clinique | Azithromycine | enum('Non renseigné','Oui','Non') |
| fiche_clinique | Posologie | varchar(50) |
| fiche_clinique | **Mesures associées** | enum('Non renseigné','Oui','Non') |
| fiche_clinique | Si oui, précisez | set('Bactrim','Vitamine D','Calcium','Régime sans sel','Régime pauvre en sucre','Autre') |
| fiche_clinique | Débit diurne (l/mn) | varchar(50) |
| fiche_clinique | Type du débit | enum('Non renseigné','Continu','Discontinu') |
| fiche_clinique | Si discontinu, durée (en h/24h) | varchar(50) |
| fiche_clinique | Si autre, précisez | longtext |
| fiche_clinique | **Nutrition entérale** | enum('Non renseigné','Oui','Non') |
| fiche_clinique | Mode d'administration | set('Sonde naso-gastrique','Gastrostomie') |
| fiche_clinique | Produit | varchar(50) |
| fiche_clinique | Dose | varchar(50) |
| fiche_clinique | Durée (h) | varchar(50) |
| fiche_clinique | Volume (ml) | varchar(50) |
| fiche_clinique | Débit (ml/h) | varchar(50) |
| fiche_clinique | **Traitement du RGO** | enum('Non renseigné','Oui','Non') |
| fiche_clinique | Si oui, précisez | set('IPP','Prokinétiques','Pansement gastrique','Autre') |
| fiche_clinique | Si autre, précisez | varchar(50) |
| fiche_clinique | **Immunosuppresseurs** | enum('Non renseigné','Oui','Non') |
| fiche_clinique | Débit nocturne (l/mn) | varchar(50) |
| fiche_clinique | Type du débit | enum('Non renseigné','Continu','Discontinu') |
| fiche_clinique | Si discontinu, durée (en h/24h) | varchar(50) |
| fiche_clinique | Molécule(s) | set('Hydroxychloroquine','Mycophénolate','Cyclophosphamide','Autre') |
| fiche_clinique | Si autre, précisez | varchar(50) |
| fiche_clinique | Date de début | date |
| fiche_clinique | Date de fin | date |
| fiche_clinique | Autre(s) traitement(s) | longtext |
| fiche_clinique | Commentaire(s) | longtext |
| fiche_clinique | **Corticothérapie** | enum('Non renseigné','Oui','Non') |
| fiche_clinique | **Prednisone** | enum('Non renseigné','Oui','Non') |
| fiche_clinique | Posologie (mg/kg) | varchar(50) |
| fiche_clinique | Posologie (mg) | varchar(50) |
| fiche_clinique | **Prednisolone** | enum('Non renseigné','Oui','Non') |
| fiche_clinique | **Oxygénothérapie** | enum('Non renseigné','Oui','Non') |
| fiche_clinique | Posologie (mg/kg) | varchar(50) |
| fiche_clinique | Posologie (mg) | varchar(50) |
| fiche_clinique | Equivalent prednisone | varchar(50) |
| fiche_clinique | **Betaméthasone** | enum('Non renseigné','Oui','Non') |
| fiche_clinique | Posologie (mg/kg) | varchar(50) |
| fiche_clinique | Posologie (mg) | varchar(50) |
| fiche_clinique | Equivalent prednisone | varchar(50) |
| fiche_clinique | **Hydrocortisone** | enum('Non renseigné','Oui','Non') |
| fiche_clinique | Posologie (mg/kg) | varchar(50) |
| fiche_clinique | Posologie (mg) | varchar(50) |
| fiche_clinique | Equivalent prednisone | varchar(50) |
| fiche_clinique | Azithromycine | enum('Non renseigné','Oui','Non') |
| fiche_clinique | Si oui, posologie | varchar(50) |
| fiche_clinique | **Mesures associées** | enum('Non renseigné','Oui','Non') |
| fiche_clinique | Si oui, précisez | set('Bactrim','Vitamine D','Calcium','Régime sans sel','Régime pauvre en sucre','Autre') |
| fiche_clinique | Débit diurne (l/mn) | varchar(50) |
| fiche_clinique | Type du débit | enum('Non renseigné','Continu','Discontinu') |
| fiche_clinique | Si discontinu, durée (en h/24h) | varchar(50) |
| fiche_clinique | Si autre, précisez | longtext |
| fiche_clinique | **Nutrition entérale** | enum('Non renseigné','Oui','Non') |
| fiche_clinique | Mode d'administration | set('Sonde naso-gastrique','Gastrostomie') |
| fiche_clinique | Produit | varchar(50) |
| fiche_clinique | Dose | varchar(50) |
| fiche_clinique | Durée (h) | varchar(50) |
| fiche_clinique | Volume (ml) | varchar(50) |
| fiche_clinique | Débit (ml/h) | varchar(50) |
| fiche_clinique | **Traitement du RGO** | enum('Non renseigné','Oui','Non') |
| fiche_clinique | Si oui, précisez | set('IPP','Prokinétiques','Pansement gastrique','Autre') |
| fiche_clinique | Si autre, précisez | varchar(50) |
| fiche_clinique | **Immunosuppresseurs** | enum('Non renseigné','Oui','Non') |
| fiche_clinique | Débit nocturne (l/mn) | varchar(50) |
| fiche_clinique | Type du débit | enum('Non renseigné','Continu','Discontinu') |
| fiche_clinique | Si discontinu, durée (en h/24h) | varchar(50) |
| fiche_clinique | Molécule(s) | set('Hydroxychloroquine','Mycophénolate','Cyclophosphamide','Autre') |
| fiche_clinique | Si autre, précisez | varchar(50) |
| fiche_clinique | Date de début | date |
| fiche_clinique | Date de fin | date |
| fiche_clinique | Autre(s) traitement(s) | longtext |
| fiche_clinique | Commentaire(s) | longtext |
| fiche_clinique | **Corticothérapie** | enum('Non renseigné','Oui','Non') |
| fiche_clinique | **Prednisone** | enum('Non renseigné','Oui','Non') |
| fiche_clinique | Posologie (mg/kg) | varchar(50) |
| fiche_clinique | Posologie (mg) | varchar(50) |
| fiche_clinique | **Prednisolone** | enum('Non renseigné','Oui','Non') |
| fiche_clinique | **Kinésithérapie** | longtext |
| fiche_clinique | **Diététique** | longtext |
| fiche_clinique | **Psychologie** | longtext |
| fin_suivi | identifiant de fin_suivi |  |
| fin_suivi | patient | int(10) unsigned |
| fin_suivi | **Date de saisie du suivi** | date |
| fin_suivi | Circonstances particulières du décès | set('Lié à un cancer (préciser ci-contre)','Lié à une transplantation d''organes','Septicémie','Autre (préciser ci-contre)') |
| fin_suivi | Autre | varchar(150) |
| fin_suivi | **Malade vu cette année** | enum('Non renseigné','Oui','Non') |
| fin_suivi | Le patient est-il | enum('Non renseigné','Vivant','Transféré','Décédé','Pas de renseignement') |
| fin_suivi | Si pas de renseignement, date de la dernière information | date |
| fin_suivi | Date du transfert | date |
| fin_suivi | Nom du centre | varchar(50) |
| fin_suivi | Médecin référent | varchar(50) |
| fin_suivi | **Si le patient est décédé** |  |
| fin_suivi | Date de décès | date |
| fin_suivi | Cause principale du décès | enum('Non renseigné','Non applicable','Cardiaque','Respiratoire','Hépatique','Traumatique','Suicide','Cause non documentée','Autre cause de décès (préciser ci-contre)') |
| fin_suivi | Autre | varchar(150) |
| fratrie | identifiant de fratrie |  |
| fratrie | clé étrangère vers patient | int(10) unsigned |
| fratrie | Rang de ce frère ou de cette sœur | varchar(50) |
| fratrie | Sexe | enum('Non renseigné','Féminin','Masculin') |
| fratrie | Atteint(e) d'une pathologie interstitielle | enum('Non renseigné','Oui','Non') |
| fratrie | Décédé(e) d'une pathologie interstitielle | enum('Non renseigné','Oui','Non') |
| fratrie | Référencé(e) dans e-Pi | enum('Non renseigné','Oui','Non') |
| fratrie | Lieu de suivi | varchar(50) |
| genetique | ID_genetique |  |
| genetique | ID_patient | int(10) unsigned |
| genetique | **Génotype de la mère** | enum('Non renseigné','Fait','Non fait') |
| genetique | Mutation dans les exons 1 à 6 | enum('Non renseigné','Présente','Absente') |
| genetique | Si présente, préciser | set('Allèle 1','Allèle 2') |
| genetique | Nom de la mutation | varchar(50) |
| genetique | Polymorphisme | varchar(50) |
| genetique | **Séquençage du gène SP-C** | enum('Non renseigné','Oui','Non') |
| genetique | Mutation I73T | enum('Non renseigné','Présente','Absente') |
| genetique | Si présente, préciser | set('Allèle 1','Allèle 2') |
| genetique | Mutation dans les exons 1 à 6 | enum('Non renseigné','Présente','Absente') |
| genetique | Si présente, préciser | set('Allèle 1','Allèle 2') |
| genetique | Nom de la mutation | varchar(50) |
| genetique | Polymorphisme | varchar(50) |
| genetique | **Séquençage du gène ABCA3** | enum('Non renseigné','Oui','Non') |
| genetique | Mutation E292V | enum('Non renseigné','Présente','Absente') |
| genetique | Si présente, préciser | set('Allèle 1','Allèle 2') |
| genetique | Origine géographique de la mère | set('Europe','Afrique du nord et Moyen orient','Afrique noire','Asie','Autre') |
| genetique | Autre origine | varchar(20) |
| genetique | Mutation dans les exons 1 à 30 | enum('Non renseigné','Présente','Absente') |
| genetique | Si présente, préciser | set('Allèle 1','Allèle 2') |
| genetique | Nom de la mutation | varchar(50) |
| genetique | Polymorphisme | varchar(50) |
| genetique | **Séquençage du gène TTF-1** | enum('Non renseigné','Oui','Non') |
| genetique | Mutation dans les exons 1 à 3 | enum('Non renseigné','Présente','Absente') |
| genetique | Si présente, préciser | set('Allèle 1','Allèle 2') |
| genetique | Nom de la mutation | varchar(50) |
| genetique | Polymorphisme | varchar(50) |
| genetique | **Syndrome Hermansky-Pudlak (type 2)** | enum('Non renseigné','Oui','Non') |
| genetique | Mutation du gène adaptine béta3A (ADBT3A) | enum('Non renseigné','Présente','Absente') |
| genetique | Si présente, préciser | set('Allèle 1','Allèle 2') |
| genetique | Nom de la mutation | varchar(50) |
| genetique | **Consentement signé** | enum('Non renseigné','Oui','Non') |
| genetique | Si oui, date | date |
| genetique | **Anomalie GMCSF** | enum('Non renseigné','Oui','Non') |
| genetique | Mutation chaîne béta du récepteur du GM-CSF (GM-CSFRbétac) | enum('Non renseigné','Présente','Absente') |
| genetique | Si présente, préciser | set('Allèle 1','Allèle 2') |
| genetique | Nom de la mutation | varchar(50) |
| genetique | **Autres mutations recherchées** | enum('Non renseigné','Oui','Non') |
| genetique | Mutation 1 | enum('Non renseigné','Présente','Absente') |
| genetique | Si présente, préciser | set('Allèle 1','Allèle 2') |
| genetique | Nom de la mutation | varchar(50) |
| genetique | Mutation 2 | enum('Non renseigné','Présente','Absente') |
| genetique | Si présente, préciser | set('Allèle 1','Allèle 2') |
| genetique | Nom de la mutation | varchar(50) |
| genetique | Mutation 3 | enum('Non renseigné','Présente','Absente') |
| genetique | Si présente, préciser | set('Allèle 1','Allèle 2') |
| genetique | Nom de la mutation | varchar(50) |
| genetique | Mutation 4 | enum('Non renseigné','Présente','Absente') |
| genetique | Si présente, préciser | set('Allèle 1','Allèle 2') |
| genetique | Nom de la mutation | varchar(50) |
| genetique | **Prélèvement sanguin effectué** | enum('Non renseigné','Oui','Non') |
| genetique | Si oui, date | date |
| genetique | Mutation 5 | enum('Non renseigné','Présente','Absente') |
| genetique | Si présente, préciser | set('Allèle 1','Allèle 2') |
| genetique | Nom de la mutation | varchar(50) |
| genetique | **Génotype du père** | enum('Non renseigné','Fait','Non fait') |
| genetique | Origine géographique du père | set('Europe','Afrique du nord et Moyen orient','Afrique noire','Asie','Autre') |
| genetique | Autre origine | varchar(20) |
| genetique | **Consentement signé** | enum('Non renseigné','Oui','Non') |
| genetique | Si oui, date | date |
| genetique | **Prélèvement sanguin effectué** | enum('Non renseigné','Oui','Non') |
| genetique | Si oui, date | date |
| genetique | **Laboratoire** | enum('Non renseigné','Trousseau','Autre') |
| genetique | Si autre laboratoire, préciser | varchar(50) |
| genetique | **N° de lot** | varchar(50) |
| genetique | **Séquençage du gène SP-B** | enum('Non renseigné','Oui','Non') |
| genetique | Mutation 121ins2 | enum('Non renseigné','Présente','Absente') |
| genetique | Si présente, préciser | set('Allèle 1','Allèle 2') |
| genetique | Mutation dans les exons 1 à 6 | enum('Non renseigné','Présente','Absente') |
| genetique | Si présente, préciser | set('Allèle 1','Allèle 2') |
| genetique | Nom de la mutation | varchar(50) |
| genetique | Polymorphisme | varchar(50) |
| genetique | **Séquençage du gène SP-C** | enum('Non renseigné','Oui','Non') |
| genetique | Mutation I73T | enum('Non renseigné','Présente','Absente') |
| genetique | Si présente, préciser | set('Allèle 1','Allèle 2') |
| genetique | **Laboratoire** | enum('Non renseigné','Trousseau','Autre') |
| genetique | *Si autre laboratoire, préciser* | varchar(50) |
| genetique | **N° de lot** | varchar(50) |
| genetique | Mutation dans les exons 1 à 6 | enum('Non renseigné','Présente','Absente') |
| genetique | Si présente, préciser | set('Allèle 1','Allèle 2') |
| genetique | Nom de la mutation | varchar(50) |
| genetique | Polymorphisme | varchar(50) |
| genetique | **Séquençage du gène ABCA3** | enum('Non renseigné','Oui','Non') |
| genetique | Mutation E292V | enum('Non renseigné','Présente','Absente') |
| genetique | Si présente, préciser | set('Allèle 1','Allèle 2') |
| genetique | Mutation dans les exons 1 à 30 | enum('Non renseigné','Présente','Absente') |
| genetique | Si présente, préciser | set('Allèle 1','Allèle 2') |
| genetique | Nom de la mutation | varchar(50) |
| genetique | Polymorphisme | varchar(50) |
| genetique | **Séquençage du gène TTF-1** | enum('Non renseigné','Oui','Non') |
| genetique | Mutation dans les exons 1 à 3 | enum('Non renseigné','Présente','Absente') |
| genetique | Si présente, préciser | set('Allèle 1','Allèle 2') |
| genetique | Nom de la mutation | varchar(50) |
| genetique | Polymorphisme | varchar(50) |
| genetique | **Syndrome Hermansky-Pudlak (type 2)** | enum('Non renseigné','Oui','Non') |
| genetique | Mutation du gène adaptine béta3A (ADBT3A) | enum('Non renseigné','Présente','Absente') |
| genetique | Si présente, préciser | set('Allèle 1','Allèle 2') |
| genetique | Nom de la mutation | varchar(50) |
| genetique | **Anomalie GMCSF** | enum('Non renseigné','Oui','Non') |
| genetique | Mutation chaîne béta du récepteur du GM-CSF (GM-CSFRbétac) | enum('Non renseigné','Présente','Absente') |
| genetique | Si présente, préciser | set('Allèle 1','Allèle 2') |
| genetique | Nom de la mutation | varchar(50) |
| genetique | **Autres mutations recherchées** | enum('Non renseigné','Oui','Non') |
| genetique | **Séquençage du gène SP-B** | enum('Non renseigné','Oui','Non') |
| genetique | Mutation 1 | enum('Non renseigné','Présente','Absente') |
| genetique | Si présente, préciser | set('Allèle 1','Allèle 2') |
| genetique | Nom de la mutation | varchar(50) |
| genetique | Mutation 2 | enum('Non renseigné','Présente','Absente') |
| genetique | Si présente, préciser | set('Allèle 1','Allèle 2') |
| genetique | Nom de la mutation | varchar(50) |
| genetique | Mutation 3 | enum('Non renseigné','Présente','Absente') |
| genetique | Si présente, préciser | set('Allèle 1','Allèle 2') |
| genetique | Nom de la mutation | varchar(50) |
| genetique | Mutation 4 | enum('Non renseigné','Présente','Absente') |
| genetique | Si présente, préciser | set('Allèle 1','Allèle 2') |
| genetique | Nom de la mutation | varchar(50) |
| genetique | Mutation 5 | enum('Non renseigné','Présente','Absente') |
| genetique | Si présente, préciser | set('Allèle 1','Allèle 2') |
| genetique | Nom de la mutation | varchar(50) |
| genetique | **Consanguinité** | enum('Non renseigné','Non','Parents cousins germains','Parents issus de germains','Autre') |
| genetique | Mutation 121ins2 | enum('Non renseigné','Présente','Absente') |
| genetique | Si présente, préciser | set('Allèle 1','Allèle 2') |
| genetique | **Commentaire(s)** | longtext |
| genetique | **Consentement signé** | enum('Non renseigné','Oui','Non') |
| genetique | Si oui, date | date |
| genetique | **Séquençage du gène SP-C** | enum('Non renseigné','Oui','Non') |
| genetique | Mutation I73T | enum('Non renseigné','Présente','Absente') |
| genetique | Si présente, préciser | set('Allèle 1','Allèle 2') |
| genetique | Mutation dans les exons 1 à 5 | enum('Non renseigné','Présente','Absente') |
| genetique | Si présente, préciser | set('Allèle 1','Allèle 2') |
| genetique | Nom de la mutation | varchar(50) |
| genetique | Polymorphisme | varchar(50) |
| genetique | **Séquençage du gène ABCA3** | enum('Non renseigné','Oui','Non') |
| genetique | Mutation E292V | enum('Non renseigné','Présente','Absente') |
| genetique | Si présente, préciser | set('Allèle 1','Allèle 2') |
| genetique | Mutation dans les exons 1 à 30 | enum('Non renseigné','Présente','Absente') |
| genetique | Si présente, préciser | set('Allèle 1','Allèle 2') |
| genetique | Nom de la mutation | varchar(50) |
| genetique | Polymorphisme | varchar(50) |
| genetique | **Prélèvement sanguin effectué** | enum('Non renseigné','Oui','Non') |
| genetique | Si oui, date | date |
| genetique | **Séquençage du gène TTF-1** | enum('Non renseigné','Oui','Non') |
| genetique | Mutation dans les exons 1 à 3 | enum('Non renseigné','Présente','Absente') |
| genetique | Si présente, préciser | set('Allèle 1','Allèle 2') |
| genetique | Nom de la mutation | varchar(50) |
| genetique | Polymorphisme | varchar(50) |
| genetique | **Syndrome Hermansky-Pudlak (type 2)** | enum('Non renseigné','Oui','Non') |
| genetique | Mutation du gène adaptine béta3A (ADBT3A) | enum('Non renseigné','Présente','Absente') |
| genetique | Si présente, préciser | set('Allèle 1','Allèle 2') |
| genetique | Nom de la mutation | varchar(50) |
| genetique | **Anomalie GMCSF** | enum('Non renseigné','Oui','Non') |
| genetique | Mutation chaîne béta du récepteur du GM-CSF (GM-CSFRbétac) | enum('Non renseigné','Présente','Absente') |
| genetique | Si présente, préciser | set('Allèle 1','Allèle 2') |
| genetique | Nom de la mutation | varchar(50) |
| genetique | **Autres mutations recherchées** | enum('Non renseigné','Oui','Non') |
| genetique | **Laboratoire** | enum('Non renseigné','Trousseau','Autre') |
| genetique | *Si autre laboratoire, préciser* | varchar(50) |
| genetique | **N° de lot** | varchar(50) |
| genetique | Mutation 1 | enum('Non renseigné','Présente','Absente') |
| genetique | Nom de la mutation | varchar(50) |
| genetique | Si présente, préciser | set('Allèle 1','Allèle 2') |
| genetique | Mutation 2 | enum('Non renseigné','Présente','Absente') |
| genetique | Nom de la mutation | varchar(50) |
| genetique | Si présente, préciser | set('Allèle 1','Allèle 2') |
| genetique | Mutation 3 | enum('Non renseigné','Présente','Absente') |
| genetique | Nom de la mutation | varchar(50) |
| genetique | Si présente, préciser | set('Allèle 1','Allèle 2') |
| genetique | Mutation 4 | enum('Non renseigné','Présente','Absente') |
| genetique | Nom de la mutation | varchar(50) |
| genetique | Si présente, préciser | set('Allèle 1','Allèle 2') |
| genetique | Mutation 5 | enum('Non renseigné','Présente','Absente') |
| genetique | Nom de la mutation | varchar(50) |
| genetique | Si présente, préciser | set('Allèle 1','Allèle 2') |
| genetique | **Séquençage du gène SP-B** | enum('Non renseigné','Oui','Non') |
| genetique | Mutation 121ins2 | enum('Non renseigné','Présente','Absente') |
| genetique | Si présente, préciser | set('Allèle 1','Allèle 2') |
| genetique | Mutation dans les exons 1 à 10 | enum('Non renseigné','Présente','Absente') |
| genetique | Si présente, préciser | set('Allèle 1','Allèle 2') |
| genetique | Nom de la mutation | varchar(50) |
| genetique | Polymorphisme | varchar(50) |
| iconographie | identifiant de iconographie |  |
| iconographie | clé étrangère fiche clinique | int(10) unsigned |
| iconographie | **Autres examens** | longtext |
| iconographie | **Coeur droit** | enum('Non renseigné','Exploré','Non exploré') |
| iconographie | Dilatation du ventricule | enum('Non renseigné','Oui','Non') |
| iconographie | Diamètre télédiastolique (mm) | varchar(5) |
| iconographie | Insuffisance tricuspide (m/s) | varchar(5) |
| iconographie | **Flux pulmonaire en doppler pulsé** | enum('Non renseigné','Mesuré','Non mesuré') |
| iconographie | **Flux d'insuffisance pulmonaire en doppler continu** | enum('Non renseigné','Mesuré','Non mesuré') |
| iconographie | Intestins | enum('Non renseigné','Epaisseur de la paroi normale','Epaisseur de la paroi augmentée') |
| iconographie | Mucocèle appendiculaire | varchar(5) |
| iconographie | Reins - Lithiase | enum('Non renseigné','Oui','Non') |
| iconographie | Commentaire échographie abdominale | longtext |
| iconographie | Gradient OD-VD + 5 ou 10 mm | varchar(5) |
| iconographie | Taille (diamètre sagittal maximal en cm) | varchar(5) |
| iconographie | Pression pulmonaire diastolique (mmHg) | varchar(5) |
| iconographie | Pression pulmonaire moyenne (mmHg) | varchar(5) |
| iconographie | Diamètre de la veine cave inférieure en expiration (mm) | varchar(5) |
| iconographie | Diamètre de la veine cave inférieure en inspiration (mm) | varchar(5) |
| iconographie | Commentaire | longtext |
| iconographie | Diamètre télédiastolique (mm) | varchar(5) |
| iconographie | **Densité minérale osseuse (Z(t))** | enum('Non renseigné','Oui','Non') |
| iconographie | Diamètre télésystolique(mm) | varchar(5) |
| iconographie | A la date du | date |
| iconographie | Fraction de raccourcissement (%) | varchar(5) |
| iconographie | Pourcentage de temps avec un pH < 4 | varchar(5) |
| iconographie | Foie | enum('Non renseigné','Normal','Hyperéchogène','Nodulaire','Scléroatrophique','Stéatose') |
| iconographie | Signes d'hypertension portale | varchar(4) |
| iconographie | Flèche hépatique (cm) | varchar(5) |
| iconographie | Septum interventriculaire (mm) | varchar(5) |
| iconographie | Paroi postérieure en TD (mm) | varchar(5) |
| iconographie | Flux mitral | varchar(5) |
| iconographie | Rate | enum('Non renseigné','Normal','Ptosée','Hypertrophique') |
| iconographie | Rapport E/A | varchar(5) |
| iconographie | Flèche (cm) | varchar(5) |
| iconographie | Temps de relaxation isovolumétrique | varchar(5) |
| iconographie | Radio normale | enum('Non renseigné','Oui','Non') |
| iconographie | Distension | enum('Non renseigné','Absent','Modéré','Important','Sévère') |
| iconographie | Epaississement péribronchique | enum('Non renseigné','Absent','Modéré','Important','Sévère') |
| iconographie | Impactions mucoïdes | enum('Non renseigné','Absent','Modéré','Important','Sévère') |
| iconographie | Bronchectasies | enum('Non renseigné','Absent','Modéré','Important','Sévère') |
| iconographie | Opacités alvéolaires et collapsus | enum('Non renseigné','Absent','Modéré','Important','Sévère') |
| iconographie | **Radiographie du thorax** | enum('Non renseigné','Oui','Non') |
| iconographie | A la date du | date |
| iconographie | **Indication** | varchar(50) |
| iconographie | Commentaire radiographie du thorax | longtext |
| iconographie | Date | date |
| iconographie | **Indication** | varchar(255) |
| iconographie | **TDM** | enum('Non renseigné','Fait','Non fait') |
| iconographie | Date de la TDM | date |
| iconographie | Bases (< vpi) | enum('Non renseigné','0','1','2','3') |
| iconographie | Central (2/3 int) | enum('Non renseigné','0','1','2','3') |
| iconographie | Périphérique (1/3 ext) | enum('Non renseigné','0','1','2','3') |
| iconographie | Bases (< vpi) | enum('Non renseigné','0','1','2','3') |
| iconographie | Central (2/3 int) | enum('Non renseigné','0','1','2','3') |
| iconographie | Périphérique (1/3 ext) | enum('Non renseigné','0','1','2','3') |
| iconographie | Antérieur (> trachée) | enum('Non renseigné','0','1','2','3') |
| iconographie | Postérieur (< trachée) | enum('Non renseigné','0','1','2','3') |
| iconographie | Aléatoire | enum('Non renseigné','0','1','2','3') |
| iconographie | **Nodules cavitaires** | enum('Non renseigné','Présent','Absent') |
| iconographie | Sommets (>bif) | enum('Non renseigné','0','1','2','3') |
| iconographie | Zone moyenne (bif-vpi) | enum('Non renseigné','0','1','2','3') |
| iconographie | Bases (< vpi) | enum('Non renseigné','0','1','2','3') |
| iconographie | Central (2/3 int) | enum('Non renseigné','0','1','2','3') |
| iconographie | Périphérique (1/3 ext) | enum('Non renseigné','0','1','2','3') |
| iconographie | Antérieur (> trachée) | enum('Non renseigné','0','1','2','3') |
| iconographie | Postérieur (< trachée) | enum('Non renseigné','0','1','2','3') |
| iconographie | Aléatoire | enum('Non renseigné','0','1','2','3') |
| iconographie | **Bronchectasie par traction** | enum('Non renseigné','Présent','Absent') |
| iconographie | Sommets (>bif) | enum('Non renseigné','0','1','2','3') |
| iconographie | Zone moyenne (bif-vpi) | enum('Non renseigné','0','1','2','3') |
| iconographie | Bases (< vpi) | enum('Non renseigné','0','1','2','3') |
| iconographie | Central (2/3 int) | enum('Non renseigné','0','1','2','3') |
| iconographie | Périphérique (1/3 ext) | enum('Non renseigné','0','1','2','3') |
| iconographie | Antérieur (> trachée) | enum('Non renseigné','0','1','2','3') |
| iconographie | Postérieur (< trachée) | enum('Non renseigné','0','1','2','3') |
| iconographie | Aléatoire | enum('Non renseigné','0','1','2','3') |
| iconographie | Antérieur (> trachée) | enum('Non renseigné','0','1','2','3') |
| iconographie | Postérieur (< trachée) | enum('Non renseigné','0','1','2','3') |
| iconographie | Aléatoire | enum('Non renseigné','0','1','2','3') |
| iconographie | **Perfusion en mosaique (si expiration)** | enum('Non renseigné','Présent','Absent') |
| iconographie | Sommets (>bif) | enum('Non renseigné','0','1','2','3') |
| iconographie | Zone moyenne (bif-vpi) | enum('Non renseigné','0','1','2','3') |
| iconographie | Bases (< vpi) | enum('Non renseigné','0','1','2','3') |
| iconographie | Central (2/3 int) | enum('Non renseigné','0','1','2','3') |
| iconographie | Périphérique (1/3 ext) | enum('Non renseigné','0','1','2','3') |
| iconographie | Antérieur (> trachée) | enum('Non renseigné','0','1','2','3') |
| iconographie | Postérieur (< trachée) | enum('Non renseigné','0','1','2','3') |
| iconographie | Aléatoire | enum('Non renseigné','0','1','2','3') |
| iconographie | **Adénopathies (si Inj)** | enum('Non renseigné','Présent','Absent') |
| iconographie | Sommets (>bif) | enum('Non renseigné','0','1','2','3') |
| iconographie | Zone moyenne (bif-vpi) | enum('Non renseigné','0','1','2','3') |
| iconographie | Bases (< vpi) | enum('Non renseigné','0','1','2','3') |
| iconographie | Central (2/3 int) | enum('Non renseigné','0','1','2','3') |
| iconographie | Périphérique (1/3 ext) | enum('Non renseigné','0','1','2','3') |
| iconographie | Antérieur (> trachée) | enum('Non renseigné','0','1','2','3') |
| iconographie | Postérieur (< trachée) | enum('Non renseigné','0','1','2','3') |
| iconographie | Aléatoire | enum('Non renseigné','0','1','2','3') |
| iconographie | **Lignes septales épaissies interlobulaires** | enum('Non renseigné','Présent','Absent') |
| iconographie | Sommets (>bif) | enum('Non renseigné','0','1','2','3') |
| iconographie | Zone moyenne (bif-vpi) | enum('Non renseigné','0','1','2','3') |
| iconographie | Bases (< vpi) | enum('Non renseigné','0','1','2','3') |
| iconographie | Central (2/3 int) | enum('Non renseigné','0','1','2','3') |
| iconographie | Périphérique (1/3 ext) | enum('Non renseigné','0','1','2','3') |
| iconographie | Antérieur (> trachée) | enum('Non renseigné','0','1','2','3') |
| iconographie | Postérieur (< trachée) | enum('Non renseigné','0','1','2','3') |
| iconographie | Aléatoire | enum('Non renseigné','0','1','2','3') |
| iconographie | **Lignes non septales (bandes parenchymateuses)** | enum('Non renseigné','Présent','Absent') |
| iconographie | Sommets (>bif) | enum('Non renseigné','0','1','2','3') |
| iconographie | Zone moyenne (bif-vpi) | enum('Non renseigné','0','1','2','3') |
| iconographie | Bases (< vpi) | enum('Non renseigné','0','1','2','3') |
| iconographie | Central (2/3 int) | enum('Non renseigné','0','1','2','3') |
| iconographie | Périphérique (1/3 ext) | enum('Non renseigné','0','1','2','3') |
| iconographie | **Statut** | enum('Non renseigné','Interprétable','Non interprétable') |
| iconographie | Antérieur (> trachée) | enum('Non renseigné','0','1','2','3') |
| iconographie | Postérieur (< trachée) | enum('Non renseigné','0','1','2','3') |
| iconographie | Aléatoire | enum('Non renseigné','0','1','2','3') |
| iconographie | **Epaississement Interstitium intralobulaire** | enum('Non renseigné','Présent','Absent') |
| iconographie | Sommets (>bif) | enum('Non renseigné','0','1','2','3') |
| iconographie | Zone moyenne (bif-vpi) | enum('Non renseigné','0','1','2','3') |
| iconographie | Bases (< vpi) | enum('Non renseigné','0','1','2','3') |
| iconographie | Central (2/3 int) | enum('Non renseigné','0','1','2','3') |
| iconographie | Périphérique (1/3 ext) | enum('Non renseigné','0','1','2','3') |
| iconographie | Antérieur (> trachée) | enum('Non renseigné','0','1','2','3') |
| iconographie | Postérieur (< trachée) | enum('Non renseigné','0','1','2','3') |
| iconographie | Aléatoire | enum('Non renseigné','0','1','2','3') |
| iconographie | **Epaississement pleural (interstitium sous-pleural)** | enum('Non renseigné','Présent','Absent') |
| iconographie | Sommets (>bif) | enum('Non renseigné','0','1','2','3') |
| iconographie | Zone moyenne (bif-vpi) | enum('Non renseigné','0','1','2','3') |
| iconographie | Bases (< vpi) | enum('Non renseigné','0','1','2','3') |
| iconographie | Central (2/3 int) | enum('Non renseigné','0','1','2','3') |
| iconographie | Périphérique (1/3 ext) | enum('Non renseigné','0','1','2','3') |
| iconographie | Antérieur (> trachée) | enum('Non renseigné','0','1','2','3') |
| iconographie | Postérieur (< trachée) | enum('Non renseigné','0','1','2','3') |
| iconographie | Aléatoire | enum('Non renseigné','0','1','2','3') |
| iconographie | *Si non interprétable, pourquoi* | longtext |
| iconographie | **Epaississement scissure** | enum('Non renseigné','Présent','Absent') |
| iconographie | Sommets (>bif) | enum('Non renseigné','0','1','2','3') |
| iconographie | Zone moyenne (bif-vpi) | enum('Non renseigné','0','1','2','3') |
| iconographie | Bases (< vpi) | enum('Non renseigné','0','1','2','3') |
| iconographie | Central (2/3 int) | enum('Non renseigné','0','1','2','3') |
| iconographie | Périphérique (1/3 ext) | enum('Non renseigné','0','1','2','3') |
| iconographie | Antérieur (> trachée) | enum('Non renseigné','0','1','2','3') |
| iconographie | Postérieur (< trachée) | enum('Non renseigné','0','1','2','3') |
| iconographie | Aléatoire | enum('Non renseigné','0','1','2','3') |
| iconographie | **Cavités en réseau (rayon de miel)** | enum('Non renseigné','Présent','Absent') |
| iconographie | Sommets (>bif) | enum('Non renseigné','0','1','2','3') |
| iconographie | Zone moyenne (bif-vpi) | enum('Non renseigné','0','1','2','3') |
| iconographie | Bases (< vpi) | enum('Non renseigné','0','1','2','3') |
| iconographie | Central (2/3 int) | enum('Non renseigné','0','1','2','3') |
| iconographie | Périphérique (1/3 ext) | enum('Non renseigné','0','1','2','3') |
| iconographie | Antérieur (> trachée) | enum('Non renseigné','0','1','2','3') |
| iconographie | Postérieur (< trachée) | enum('Non renseigné','0','1','2','3') |
| iconographie | Aléatoire | enum('Non renseigné','0','1','2','3') |
| iconographie | **Opacités nodulaires** | enum('Non renseigné','Présent','Absent') |
| iconographie | Si fait, préciser | enum('Non renseigné','Micronodules < 7 mm','Nodules 7-30','Zone de condensation > 30 mm (pseudo-masses)') |
| iconographie | Si micronodules < 7 mm, préciser | enum('Non renseigné','Centrolobulaire','Périlymphatique','Aléatoire') |
| iconographie | Sommets (>bif) | enum('Non renseigné','0','1','2','3') |
| iconographie | Zone moyenne (bif-vpi) | enum('Non renseigné','0','1','2','3') |
| iconographie | Bases (< vpi) | enum('Non renseigné','0','1','2','3') |
| iconographie | Central (2/3 int) | enum('Non renseigné','0','1','2','3') |
| iconographie | Périphérique (1/3 ext) | enum('Non renseigné','0','1','2','3') |
| iconographie | Antérieur (> trachée) | enum('Non renseigné','0','1','2','3') |
| iconographie | Postérieur (< trachée) | enum('Non renseigné','0','1','2','3') |
| iconographie | Aléatoire | enum('Non renseigné','0','1','2','3') |
| iconographie | Sommets (>bif) | enum('Non renseigné','0','1','2','3') |
| iconographie | Zone moyenne (bif-vpi) | enum('Non renseigné','0','1','2','3') |
| iconographie | Bases (< vpi) | enum('Non renseigné','0','1','2','3') |
| iconographie | Central (2/3 int) | enum('Non renseigné','0','1','2','3') |
| iconographie | Périphérique (1/3 ext) | enum('Non renseigné','0','1','2','3') |
| iconographie | Antérieur (> trachée) | enum('Non renseigné','0','1','2','3') |
| iconographie | Postérieur (< trachée) | enum('Non renseigné','0','1','2','3') |
| iconographie | Aléatoire | enum('Non renseigné','0','1','2','3') |
| iconographie | Sommets (>bif) | enum('Non renseigné','0','1','2','3') |
| iconographie | Zone moyenne (bif-vpi) | enum('Non renseigné','0','1','2','3') |
| iconographie | Bases (< vpi) | enum('Non renseigné','0','1','2','3') |
| iconographie | Central (2/3 int) | enum('Non renseigné','0','1','2','3') |
| iconographie | Périphérique (1/3 ext) | enum('Non renseigné','0','1','2','3') |
| iconographie | **Epaississement péribroncho-vasculaire** | enum('Non renseigné','Présent','Absent') |
| iconographie | *Si fait, préciser* | enum('Non renseigné','Régulier','Irrégulier') |
| iconographie | Antérieur (> trachée) | enum('Non renseigné','0','1','2','3') |
| iconographie | Postérieur (< trachée) | enum('Non renseigné','0','1','2','3') |
| iconographie | Aléatoire | enum('Non renseigné','0','1','2','3') |
| iconographie | Sommets (>bif) | enum('Non renseigné','0','1','2','3') |
| iconographie | Zone moyenne (bif-vpi) | enum('Non renseigné','0','1','2','3') |
| iconographie | Bases (< vpi) | enum('Non renseigné','0','1','2','3') |
| iconographie | Central (2/3 int) | enum('Non renseigné','0','1','2','3') |
| iconographie | Périphérique (1/3 ext) | enum('Non renseigné','0','1','2','3') |
| iconographie | Antérieur (> trachée) | enum('Non renseigné','0','1','2','3') |
| iconographie | Postérieur (< trachée) | enum('Non renseigné','0','1','2','3') |
| iconographie | Aléatoire | enum('Non renseigné','0','1','2','3') |
| iconographie | Sommets (>bif) | enum('Non renseigné','0','1','2','3') |
| iconographie | Zone moyenne (bif-vpi) | enum('Non renseigné','0','1','2','3') |
| iconographie | Bases (< vpi) | enum('Non renseigné','0','1','2','3') |
| iconographie | Central (2/3 int) | enum('Non renseigné','0','1','2','3') |
| iconographie | Périphérique (1/3 ext) | enum('Non renseigné','0','1','2','3') |
| iconographie | Antérieur (> trachée) | enum('Non renseigné','0','1','2','3') |
| iconographie | Postérieur (< trachée) | enum('Non renseigné','0','1','2','3') |
| iconographie | Aléatoire | enum('Non renseigné','0','1','2','3') |
| iconographie | **Images en verre dépoli** | enum('Non renseigné','Présent','Absent') |
| iconographie | Sommets (>bif) | enum('Non renseigné','0','1','2','3') |
| iconographie | Zone moyenne (bif-vpi) | enum('Non renseigné','0','1','2','3') |
| iconographie | Sommets (>bif) | enum('Non renseigné','0','1','2','3') |
| iconographie | Zone moyenne (bif-vpi) | enum('Non renseigné','0','1','2','3') |
| iconographie | Bases (< vpi) | enum('Non renseigné','0','1','2','3') |
| iconographie | Central (2/3 int) | enum('Non renseigné','0','1','2','3') |
| iconographie | Périphérique (1/3 ext) | enum('Non renseigné','0','1','2','3') |
| iconographie | Antérieur (> trachée) | enum('Non renseigné','0','1','2','3') |
| iconographie | Postérieur (< trachée) | enum('Non renseigné','0','1','2','3') |
| iconographie | Aléatoire | enum('Non renseigné','0','1','2','3') |
| iconographie | **'Crazy-paving'** | enum('Non renseigné','Présent','Absent') |
| iconographie | Sommets (>bif) | enum('Non renseigné','0','1','2','3') |
| iconographie | Zone moyenne (bif-vpi) | enum('Non renseigné','0','1','2','3') |
| iconographie | Bases (< vpi) | enum('Non renseigné','0','1','2','3') |
| iconographie | Central (2/3 int) | enum('Non renseigné','0','1','2','3') |
| iconographie | Périphérique (1/3 ext) | enum('Non renseigné','0','1','2','3') |
| iconographie | Antérieur (> trachée) | enum('Non renseigné','0','1','2','3') |
| iconographie | Postérieur (< trachée) | enum('Non renseigné','0','1','2','3') |
| iconographie | Aléatoire | enum('Non renseigné','0','1','2','3') |
| iconographie | **Opacités alvéolaires ou pseudo-alvéolaires** | enum('Non renseigné','Présent','Absent') |
| iconographie | Sommets (>bif) | enum('Non renseigné','0','1','2','3') |
| iconographie | Zone moyenne (bif-vpi) | enum('Non renseigné','0','1','2','3') |
| iconographie | Bases (< vpi) | enum('Non renseigné','0','1','2','3') |
| iconographie | Central (2/3 int) | enum('Non renseigné','0','1','2','3') |
| iconographie | Périphérique (1/3 ext) | enum('Non renseigné','0','1','2','3') |
| iconographie | Bases (< vpi) | enum('Non renseigné','0','1','2','3') |
| iconographie | Central (2/3 int) | enum('Non renseigné','0','1','2','3') |
| iconographie | Périphérique (1/3 ext) | enum('Non renseigné','0','1','2','3') |
| iconographie | Antérieur (> trachée) | enum('Non renseigné','0','1','2','3') |
| iconographie | Postérieur (< trachée) | enum('Non renseigné','0','1','2','3') |
| iconographie | Aléatoire | enum('Non renseigné','0','1','2','3') |
| iconographie | **Calcifications** | enum('Non renseigné','Présent','Absent') |
| iconographie | Sommets (>bif) | enum('Non renseigné','0','1','2','3') |
| iconographie | Zone moyenne (bif-vpi) | enum('Non renseigné','0','1','2','3') |
| iconographie | Bases (< vpi) | enum('Non renseigné','0','1','2','3') |
| iconographie | Central (2/3 int) | enum('Non renseigné','0','1','2','3') |
| iconographie | Périphérique (1/3 ext) | enum('Non renseigné','0','1','2','3') |
| iconographie | Antérieur (> trachée) | enum('Non renseigné','0','1','2','3') |
| iconographie | Postérieur (< trachée) | enum('Non renseigné','0','1','2','3') |
| iconographie | Aléatoire | enum('Non renseigné','0','1','2','3') |
| iconographie | Antérieur (> trachée) | enum('Non renseigné','0','1','2','3') |
| iconographie | Postérieur (< trachée) | enum('Non renseigné','0','1','2','3') |
| iconographie | Aléatoire | enum('Non renseigné','0','1','2','3') |
| iconographie | **Kystes (< 3 mm)** | enum('Non renseigné','Présent','Absent') |
| iconographie | Sommets (>bif) | enum('Non renseigné','0','1','2','3') |
| iconographie | Zone moyenne (bif-vpi) | enum('Non renseigné','0','1','2','3') |
| iconographie | Bases (< vpi) | enum('Non renseigné','0','1','2','3') |
| iconographie | Central (2/3 int) | enum('Non renseigné','0','1','2','3') |
| iconographie | Périphérique (1/3 ext) | enum('Non renseigné','0','1','2','3') |
| iconographie | Antérieur (> trachée) | enum('Non renseigné','0','1','2','3') |
| iconographie | Postérieur (< trachée) | enum('Non renseigné','0','1','2','3') |
| iconographie | Aléatoire | enum('Non renseigné','0','1','2','3') |
| iconographie | **Emphysème** | enum('Non renseigné','Présent','Absent') |
| iconographie | Si fait, préciser | enum('Non renseigné','Centro-lobulaire','Pan-lobulaire','Para-septal') |
| iconographie | Sommets (>bif) | enum('Non renseigné','0','1','2','3') |
| iconographie | Zone moyenne (bif-vpi) | enum('Non renseigné','0','1','2','3') |
| iconographie | Bases (< vpi) | enum('Non renseigné','0','1','2','3') |
| iconographie | Central (2/3 int) | enum('Non renseigné','0','1','2','3') |
| iconographie | Périphérique (1/3 ext) | enum('Non renseigné','0','1','2','3') |
| iconographie | Antérieur (> trachée) | enum('Non renseigné','0','1','2','3') |
| iconographie | Postérieur (< trachée) | enum('Non renseigné','0','1','2','3') |
| iconographie | Aléatoire | enum('Non renseigné','0','1','2','3') |
| iconographie | Sommets (>bif) | enum('Non renseigné','0','1','2','3') |
| iconographie | Zone moyenne (bif-vpi) | enum('Non renseigné','0','1','2','3') |
| iconographie | Sommets (>bif) | enum('Non renseigné','0','1','2','3') |
| iconographie | Zone moyenne (bif-vpi) | enum('Non renseigné','0','1','2','3') |
| iconographie | Bases (< vpi) | enum('Non renseigné','0','1','2','3') |
| iconographie | Central (2/3 int) | enum('Non renseigné','0','1','2','3') |
| iconographie | Périphérique (1/3 ext) | enum('Non renseigné','0','1','2','3') |
| iconographie | Antérieur (> trachée) | enum('Non renseigné','0','1','2','3') |
| iconographie | Postérieur (< trachée) | enum('Non renseigné','0','1','2','3') |
| iconographie | Aléatoire | enum('Non renseigné','0','1','2','3') |
| iconographie | Sommets (>bif) | enum('Non renseigné','0','1','2','3') |
| iconographie | Zone moyenne (bif-vpi) | enum('Non renseigné','0','1','2','3') |
| iconographie | Bases (< vpi) | enum('Non renseigné','0','1','2','3') |
| iconographie | Central (2/3 int) | enum('Non renseigné','0','1','2','3') |
| iconographie | Périphérique (1/3 ext) | enum('Non renseigné','0','1','2','3') |
| iconographie | Antérieur (> trachée) | enum('Non renseigné','0','1','2','3') |
| iconographie | Postérieur (< trachée) | enum('Non renseigné','0','1','2','3') |
| iconographie | Aléatoire | enum('Non renseigné','0','1','2','3') |
| iconographie | **Bulles** | enum('Non renseigné','Présent','Absent') |
| iconographie | Sommets (>bif) | enum('Non renseigné','0','1','2','3') |
| iconographie | Zone moyenne (bif-vpi) | enum('Non renseigné','0','1','2','3') |
| iconographie | **Scintigraphie pulmonaire** | enum('Non renseigné','Oui','Non') |
| iconographie | A la date du | date |
| iconographie | Si oui, préciser | set('Ventilation','Perfusion') |
| iconographie | **Indication** | varchar(50) |
| iconographie | Résultat | longtext |
| iconographie | **Conclusion** | longtext |
| iconographie | Image 1 | varchar(50) |
| iconographie | Image 2 | varchar(50) |
| iconographie | Image 3 | varchar(50) |
| iconographie | Image 4 | varchar(50) |
| iconographie | Image 5 | varchar(50) |
| immunologie | ID_immunologique |  |
| immunologie | ID_fiche_clinique | int(10) unsigned |
| immunologie | **Facteurs rhumatoïdes** |  |
| immunologie | **EBV** | enum('Non renseigné','Fait','Non fait') |
| immunologie | **Anticorps anti-tissus** |  |
| immunologie | **Sérologies postvaccinales** | enum('Non renseigné','Faites','Non faites') |
| immunologie | Ac anti-rougeole (UI/ml) | varchar(50) |
| immunologie | Titre | enum('Non renseigné','Normal','Faible') |
| immunologie | Ac anti-oreillons (UI/ml) | varchar(50) |
| immunologie | Titre | enum('Non renseigné','Normal','Faible') |
| immunologie | Ac anti-rubeole (UI/ml) | varchar(50) |
| immunologie | Titre | enum('Non renseigné','Normal','Faible') |
| immunologie | Ac anti-coqueluche (UI/ml) | varchar(50) |
| immunologie | Titre | enum('Non renseigné','Normal','Faible') |
| immunologie | **Sérologies des infections** | enum('Non renseigné','Faites','Non faites') |
| immunologie | Ac anti-diphtériques (UI/ml) | varchar(50) |
| immunologie | Titre | enum('Non renseigné','Normal','Faible') |
| immunologie | **Aspergillus fumigatus** | enum('Non renseigné','Fait','Non fait') |
| immunologie | Méthode utilisée | set('Electrophorèse','Immuno électrophorèse','Autre') |
| immunologie | *Si autre, préciser* | varchar(50) |
| immunologie | Antigène métabolique (nombre d'arcs) | varchar(50) |
| immunologie | Antigène somatique (nombre d'arcs) | varchar(50) |
| immunologie | **Hépatite A** | enum('Non renseigné','Fait','Non fait') |
| immunologie | IgG VHA (UI/L) | varchar(20) |
| immunologie | IgM VHA (UI/L) | varchar(50) |
| immunologie | **Hépatite B** | enum('Non renseigné','Fait','Non fait') |
| immunologie | Ag HbS (UI/L) | varchar(50) |
| immunologie | Anticorps anti-HbS (UI/L) | varchar(50) |
| immunologie | Ag Hbe (UI/L) | varchar(50) |
| immunologie | Anticorps anti-Hbe (UI/L) | varchar(50) |
| immunologie | IgM anti-HbC (UI/L) | varchar(50) |
| immunologie | DNA virase | varchar(50) |
| immunologie | Ac anti-tetaniques (UI/ml) | varchar(50) |
| immunologie | Titre | enum('Non renseigné','Normal','Faible') |
| immunologie | **HSV** | enum('Non renseigné','Fait','Non fait') |
| immunologie | IgG (UA/mL) | varchar(50) |
| immunologie | IgM (UA/mL) | varchar(50) |
| immunologie | **Hépatite C** | enum('Non renseigné','Fait','Non fait') |
| immunologie | Anticorps anti-HCV (UI/L) | varchar(50) |
| immunologie | PCR HCV | enum('Non renseigné','Oui','Non') |
| immunologie | **Autres virus** | enum('Non renseigné','Oui','Non') |
| immunologie | **CMV** | enum('Non renseigné','Fait','Non fait') |
| immunologie | IgG (UA/mL) | varchar(50) |
| immunologie | IgM (UA/mL) | varchar(50) |
| immunologie | **Adénovirus** | enum('Non renseigné','Fait','Non fait') |
| immunologie | IgG (UA/mL) | varchar(50) |
| immunologie | IgM (UA/mL) | varchar(50) |
| immunologie | **EBV MNI test** | enum('Non renseigné','Positif','Négatif') |
| immunologie | **VCA** | enum('Non renseigné','Fait','Non fait') |
| immunologie | IgG (UA/mL) | varchar(50) |
| immunologie | IgM (UA/mL) | varchar(50) |
| immunologie | Ac anti-polio type I (UI/ml) | varchar(50) |
| immunologie | Titre | enum('Non renseigné','Normal','Faible') |
| immunologie | **EBNA** | enum('Non renseigné','Fait','Non fait') |
| immunologie | IgG (UA/mL) | varchar(50) |
| immunologie | anti EA (UA/mL) | varchar(50) |
| immunologie | **VZV** | enum('Non renseigné','Fait','Non fait') |
| immunologie | IgG (UA/mL) | varchar(50) |
| immunologie | IgM (UA/mL) | varchar(50) |
| immunologie | **Toxoplasmose** | enum('Non renseigné','Fait','Non fait') |
| immunologie | IgG (UI/mL) | varchar(50) |
| immunologie | IgM (UI/mL) | varchar(50) |
| immunologie | **Syphillis** | enum('Non renseigné','Fait','Non fait') |
| immunologie | TPHA | enum('Non renseigné','Oui','Non') |
| immunologie | VDRL | enum('Non renseigné','Oui','Non') |
| immunologie | **HIV** | enum('Non renseigné','Positif','Négatif') |
| immunologie | HTLV 1 | enum('Non renseigné','Positif','Négatif') |
| immunologie | HTLV 2 | enum('Non renseigné','Positif','Négatif') |
| immunologie | Ac anti-polio type II (UI/ml) | varchar(50) |
| immunologie | Titre | enum('Non renseigné','Normal','Faible') |
| immunologie | **Mycoplasma Pneumoniae** | enum('Non renseigné','Fait','Non fait') |
| immunologie | IgG (UI/mL) | varchar(50) |
| immunologie | IgM (UI/mL) | varchar(50) |
| immunologie | **Chlamydia trachomatis** | enum('Non renseigné','Fait','Non fait') |
| immunologie | Résultat | enum('Non renseigné','Positif','Négatif') |
| immunologie | IgG (UA/mL) | varchar(50) |
| immunologie | IgM (UA/mL) | varchar(50) |
| immunologie | **Chlamydia pneumoniae** | enum('Non renseigné','Fait','Non fait') |
| immunologie | Résultat | enum('Non renseigné','Positif','Négatif') |
| immunologie | IgG (UA/mL) | varchar(50) |
| immunologie | IgM (UA/mL) | varchar(50) |
| immunologie | Chlamydia psittaci | enum('Non renseigné','Fait','Non fait') |
| immunologie | Résultat | enum('Non renseigné','Positif','Négatif') |
| immunologie | IgG (UA/mL) | varchar(50) |
| immunologie | IgM (UA/mL) | varchar(50) |
| immunologie | **Legionelle** | enum('Non renseigné','Fait','Non fait') |
| immunologie | IgG (UA/mL) | varchar(50) |
| immunologie | IgM (UA/mL) | varchar(50) |
| immunologie | Ac anti-polio type III (UI/ml) | varchar(50) |
| immunologie | Titre | enum('Non renseigné','Normal','Faible') |
| immunologie | **Ureaplasma urealyticum** | enum('Non renseigné','Fait','Non fait') |
| immunologie | IgG (UA/mL) | varchar(50) |
| immunologie | IgM (UA/mL) | varchar(50) |
| immunologie | **Poumon de fermier** | enum('Non renseigné','Fait','Non fait') |
| immunologie | Résultat | enum('Non renseigné','Positif','Négatif') |
| immunologie | Préciser | varchar(50) |
| immunologie | **Poumon eleveur d'oiseaux** | enum('Non renseigné','Fait','Non fait') |
| immunologie | Résultat | enum('Non renseigné','Positif','Négatif') |
| immunologie | Préciser | varchar(50) |
| immunologie | **Précipitines anti-aviaires (nombre d'arcs)** | varchar(50) |
| immunologie | **Commentaires** | longtext |
| immunologie | Ac anti-pneumocoque (UI/ml) | varchar(50) |
| immunologie | Titre | enum('Non renseigné','Normal','Faible') |
| immunologie | Ac anti-meningocoque (UI/ml) | varchar(50) |
| immunologie | Titre | enum('Non renseigné','Normal','Faible') |
| immunologie | Ac anti-haemophilus (UI/ml) | varchar(50) |
| immunologie | Titre | enum('Non renseigné','Normal','Faible') |
| immunologie | **IgG (g/L)** | varchar(50) |
| immunologie | **Immunité humorale** | enum('Non renseigné','Fait','Non fait') |
| immunologie | **Complément** | enum('Non renseigné','Fait','Non fait') |
| immunologie | C3 (mg/L) | varchar(50) |
| immunologie | C4 (mg/L) | varchar(50) |
| immunologie | CH50 ou complément total (%) | varchar(50) |
| immunologie | Hémagglutines de groupe sanguin | varchar(50) |
| immunologie | **Immunité cellulaire** | enum('Non renseigné','Fait','Non fait') |
| immunologie | Intradermoréaction à la tuberculine (IDR) | enum('Non renseigné','Fait','Non fait') |
| immunologie | Si fait, date | date |
| immunologie | Résultat IDR (mm) | varchar(50) |
| immunologie | Lue à | enum('Non renseigné','48h','72h') |
| immunologie | IgG 1 (g/L) | varchar(20) |
| immunologie | IgG 2 (g/L) | varchar(20) |
| immunologie | IgG 3 (g/L) | varchar(20) |
| immunologie | IgG 4 (g/L) | varchar(20) |
| immunologie | Phlycténulaire | enum('Non renseigné','Fait','Non fait') |
| immunologie | **Etude des lymphocytes** | enum('Non renseigné','Fait','Non fait') |
| immunologie | **Phénotypage lymphocytaire** |  |
| immunologie | **Lymphocytes totaux (10^9/l)** | varchar(50) |
| immunologie | **Population T** |  |
| immunologie | CD3+ (%) | varchar(50) |
| immunologie | CD3+ | varchar(50) |
| immunologie |  | enum('Non renseigné','10^9/L','/microL') |
| immunologie | CD4+ (%) | varchar(50) |
| immunologie | CD4+ | varchar(50) |
| immunologie |  | enum('Non renseigné','10^9/L','/microL') |
| immunologie | CD8+ (%) | varchar(50) |
| immunologie | CD8+ | varchar(50) |
| immunologie |  | enum('Non renseigné','10^9/L','/microL') |
| immunologie | Rapport CD4/CD8 | varchar(50) |
| immunologie | CD4+/ CD3+ (%) | varchar(50) |
| immunologie | CD4+/ CD3+ (10^9/l) | varchar(50) |
| immunologie | CD8+/CD3+ (%) | varchar(50) |
| immunologie | CD8+/CD3+ (10^9/l) | varchar(50) |
| immunologie | Test de prolifération lymphocytaire T | set('Non stimulé à J3','PHA','Non stimulé à J6','Tuberculine','Candidine','Anatoxine tétanique') |
| immunologie | Valeur 'Non stimulé à J3' (cpm/10^3) | varchar(50) |
| immunologie | Valeur 'PHA' (cpm/10^3) | varchar(50) |
| immunologie | Valeur 'Non stimulé à J6' (cpm/10^3) | varchar(50) |
| immunologie | Valeur 'Tuberculine' (cpm/10^3) | varchar(50) |
| immunologie | Valeur 'Candidine' (cpm/10^3) | varchar(50) |
| immunologie | Valeur 'Anatoxine tétanique' (cpm/10^3) | varchar(50) |
| immunologie | Résultat | enum('Non renseigné','Normal','Anormal') |
| immunologie | Commentaire(s) | longtext |
| immunologie | **Population B** |  |
| immunologie | CD19 (%) | varchar(50) |
| immunologie | CD19 (10^9/l) | varchar(50) |
| immunologie | **Population NK** |  |
| immunologie | CD56+/CD3- (%) | varchar(50) |
| immunologie | CD56+/CD3- (10^9/l) | varchar(50) |
| immunologie | Test de transformation lymphoblastique | set('Aux antigènes','Aux mitogènes') |
| immunologie | Résultat (aux antigènes) | enum('Non renseigné','Normal','Anormal') |
| immunologie |  | varchar(50) |
| immunologie | Résultat (aux mitogènes) | enum('Non renseigné','Normal','Anormal') |
| immunologie |  | varchar(50) |
| immunologie | **Etude des polynucléaires** | enum('Non renseigné','Fait','Non fait') |
| immunologie | NBT test (explosion oxydative) | enum('Non renseigné','Fait','Non fait') |
| immunologie | Si fait, précisez | enum('Non renseigné','Normal','Anormal') |
| immunologie | **IgA (g/L)** | varchar(20) |
| immunologie | **IgM (g/L)** | varchar(20) |
| immunologie | Si anormal, précisez | longtext |
| immunologie | Mouvement des polynucléaires neutrophiles | enum('Non renseigné','Fait','Non fait') |
| immunologie | Si fait, précisez | enum('Non renseigné','Normal','Anormal') |
| immunologie | Si anormal, précisez | longtext |
| immunologie | Commentaire | longtext |
| immunologie | Date de l'examen | date |
| immunologie | **Date de la prescription** | date |
| immunologie | Coombs direct | enum('Non renseigné','Positif','Négatif') |
| immunologie | Ac anti-antigènes solubles | enum('Non renseigné','Positif','Négatif') |
| immunologie | Anticorps anti HLA I | enum('Non renseigné','Positif','Négatif') |
| immunologie | Valeur (UI/L) | varchar(50) |
| immunologie | Anticorps anti HLA II | enum('Non renseigné','Positif','Négatif') |
| immunologie | Valeur (UI/L) | varchar(50) |
| immunologie | Anticorps anti DNA natif | enum('Non renseigné','Positif','Négatif') |
| immunologie | Valeur (UI/ml) | varchar(50) |
| immunologie | Ac anti-cytoplasme des PN | enum('Non renseigné','Positif','Négatif') |
| immunologie | Valeur | varchar(50) |
| immunologie | Ac anti-mitochondries | enum('Non renseigné','Positif','Négatif') |
| immunologie | Type | set('2','5','6') |
| immunologie | Valeur (type 2) | varchar(50) |
| immunologie | Valeur (type 5) | varchar(50) |
| immunologie | Valeur (type 6) | varchar(50) |
| immunologie | Ac anti-cytosol hépatique 1 (LC1) | enum('Non renseigné','Positif','Négatif') |
| immunologie | Valeur | varchar(50) |
| immunologie | Ac anti-muscle lisse | enum('Non renseigné','Positif','Négatif') |
| immunologie | Valeur | varchar(50) |
| immunologie | Coombs indirect | enum('Non renseigné','Positif','Négatif') |
| immunologie | Ac anti-muscle strié | enum('Non renseigné','Positif','Négatif') |
| immunologie | Valeur | varchar(50) |
| immunologie | Ac anti-membrane basale épidermique | enum('Non renseigné','Positif','Négatif') |
| immunologie | Valeur | varchar(50) |
| immunologie | Ac anti-reticulum endoplasmique | enum('Non renseigné','Positif','Négatif') |
| immunologie | Valeur | varchar(50) |
| immunologie | Ac anti-plaquettes | enum('Non renseigné','Positif','Négatif') |
| immunologie | Valeur | varchar(50) |
| immunologie | Ac anti-cardiolipine (isotype G) | enum('Non renseigné','Positif','Négatif') |
| immunologie | Valeur | varchar(50) |
| immunologie | Ac anti-cardiolipine (isotype M) | enum('Non renseigné','Positif','Négatif') |
| immunologie | Valeur | varchar(50) |
| immunologie | Ac anti-coagulants circulants de type lupique | enum('Non renseigné','Positif','Négatif') |
| immunologie | Valeur | varchar(50) |
| immunologie | RAI | enum('Non renseigné','Positif','Négatif') |
| immunologie | Ac anti-microsomes foie/rein (LKM1) | enum('Non renseigné','Positif','Négatif') |
| immunologie | Valeur | varchar(50) |
| immunologie | Ac anti-microsomes thyroïdien | enum('Non renseigné','Positif','Négatif') |
| immunologie | Valeur | varchar(50) |
| immunologie | Ac anti-thyroperoxydase | enum('Non renseigné','Positif','Négatif') |
| immunologie | Valeur | varchar(50) |
| immunologie | Ac anti-thyroglobuline | enum('Non renseigné','Positif','Négatif') |
| immunologie | Valeur | varchar(50) |
| immunologie | AC anti-histones | enum('Non renseigné','Positif','Négatif') |
| immunologie | Valeur | varchar(50) |
| immunologie | Ac anti-kératine | enum('Non renseigné','Positif','Négatif') |
| immunologie | Valeur | varchar(50) |
| immunologie | Cryoglobulinémie | enum('Non renseigné','Positif','Négatif') |
| immunologie | Valeur (g/L) | varchar(50) |
| immunologie | Ac anti-nucléosome | enum('Non renseigné','Positif','Négatif') |
| immunologie | Valeur (UDO) | varchar(50) |
| immunologie | CIC | enum('Non renseigné','Positif','Négatif') |
| immunologie | Valeur (microg/ml) | varchar(50) |
| immunologie | **Intolérance au gluten** | enum('Non renseigné','Fait','Non fait') |
| immunologie | Ac anti-gliadine (isotype A) | enum('Non renseigné','Positif','Négatif') |
| immunologie | Valeur | varchar(50) |
| immunologie | Ac anti-gliadine (isotype G) | enum('Non renseigné','Positif','Négatif') |
| immunologie | Valeur | varchar(50) |
| immunologie | Ac anti-endomysium (isotype A) | enum('Non renseigné','Positif','Négatif') |
| immunologie | Valeur | varchar(50) |
| immunologie | Ac anti-transglutaminase (isotype A) | enum('Non renseigné','Positif','Négatif') |
| immunologie | Valeur | varchar(50) |
| immunologie | Ac anti-reticuline (isotype A) | enum('Non renseigné','Positif','Négatif') |
| immunologie | Valeur | varchar(50) |
| immunologie | **Autre(s)** | longtext |
| immunologie | Test au Latex | enum('Non renseigné','Positif','Négatif') |
| immunologie | Valeur | varchar(50) |
| immunologie | **Synthèse de l'examen immunologique** | longtext |
| immunologie | Réaction de Waaler-Rose | enum('Non renseigné','Positif','Négatif') |
| immunologie | Valeur | varchar(50) |
| immunologie | Ac anti-membrane basale glomérulaire | enum('Non renseigné','Positif','Négatif') |
| immunologie | Valeur | varchar(50) |
| immunologie | Facteur rhumatoïde | enum('Non renseigné','Positif','Négatif') |
| immunologie | Valeur (UI/mL) | varchar(50) |
| immunologie | Ac anti-nucléraires | enum('Non renseigné','Positif','Négatif') |
| immunologie | Valeur (UI/ml) | varchar(50) |
| immunologie | **IgE totales (KUI/l)** | varchar(50) |
| immunologie | Si autre animal, précisez | varchar(50) |
| immunologie | RAST chat(valeur absolue) | varchar(50) |
| immunologie | RAST chien(valeur absolue) | varchar(50) |
| immunologie | RAST cheval(valeur absolue) | varchar(50) |
| immunologie | RAST blatte(valeur absolue) | varchar(50) |
| immunologie | RAST autre animal(valeur absolue) | varchar(50) |
| immunologie | Témoin positif | enum('Non renseigné','Non fait','Négatif','Positif') |
| immunologie | RAST (valeur absolue) | varchar(50) |
| immunologie | **Pollens de printemps** | enum('Non renseigné','Non fait','Négatif','Positif') |
| immunologie | Si positif, précisez | set('Graminées','Plantain','Bouleau','Frêne','Chêne','Platane','Olivier','Autre') |
| immunologie | Si autre pollen de printemps, précisez | varchar(50) |
| immunologie | RAST graminées(valeur absolue) | varchar(50) |
| immunologie | RAST plantain(valeur absolue) | varchar(50) |
| immunologie | RAST bouleau(valeur absolue) | varchar(50) |
| immunologie | RAST frêne(valeur absolue) | varchar(50) |
| immunologie | RAST chêne(valeur absolue) | varchar(50) |
| immunologie | RAST platane(valeur absolue) | varchar(50) |
| immunologie | RAST olivier(valeur absolue) | varchar(50) |
| immunologie | Témoin négatif | enum('Non renseigné','Non fait','Négatif','Positif') |
| immunologie | RAST (valeur absolue) | varchar(50) |
| immunologie | RAST autre pollen de printemps(valeur absolue) | varchar(50) |
| immunologie | **Pollens d'été** | enum('Non renseigné','Non fait','Négatif','Positif') |
| immunologie | Si positif, précisez | set('Armoise','Pariétaire','Chénopode','Autre') |
| immunologie | Si autre pollen d'été, précisez | varchar(50) |
| immunologie | RAST armoise(valeur absolue) | varchar(50) |
| immunologie | RAST pariétaire(valeur absolue) | varchar(50) |
| immunologie | RAST chénopode(valeur absolue) | varchar(50) |
| immunologie | RAST autre pollen d'été(valeur absolue) | varchar(50) |
| immunologie | **Acariens** | enum('Non renseigné','Non fait','Négatif','Positif') |
| immunologie | **Pollens d'hiver** | enum('Non renseigné','Non fait','Négatif','Positif') |
| immunologie | Si positif, précisez | set('Cyprès','Noisetier','Aulne','Autre') |
| immunologie | Si autre pollen d'hiver, précisez | varchar(50) |
| immunologie | RAST cyprès(valeur absolue) | varchar(50) |
| immunologie | RAST noisetier(valeur absolue) | varchar(50) |
| immunologie | RAST aulne(valeur absolue) | varchar(50) |
| immunologie | RAST autre pollen d'hiver(valeur absolue) | varchar(50) |
| immunologie | **Moisissures** | enum('Non renseigné','Non fait','Négatif','Positif') |
| immunologie | Alternaria | enum('Non renseigné','Non fait','Négatif','Positif') |
| immunologie | RAST (valeur absolue) | varchar(50) |
| immunologie | Dermatophagoïdes Pteronyssinus | enum('Non renseigné','Non fait','Négatif','Positif') |
| immunologie | RAST (valeur absolue) | varchar(50) |
| immunologie | Aspergillus | enum('Non renseigné','Non fait','Négatif','Positif') |
| immunologie | RAST (valeur absolue) | varchar(50) |
| immunologie | Autre(s) moisissure(s) | enum('Non renseigné','Oui','Non') |
| immunologie | Si oui, précisez | set('Cladosporium','Penicillium','M2','M3','M4') |
| immunologie | RAST cladosporium(valeur absolue) | varchar(50) |
| immunologie | RAST penicillium(valeur absolue) | varchar(50) |
| immunologie | RAST M2(valeur absolue) | varchar(50) |
| immunologie | RAST M3(valeur absolue) | varchar(50) |
| immunologie | RAST M4(valeur absolue) | varchar(50) |
| immunologie | **Aliments** | enum('Non renseigné','Non fait','Négatif','Positif') |
| immunologie | Dermatophagoides Farinae | enum('Non renseigné','Non fait','Négatif','Positif') |
| immunologie | RAST (valeur absolue) | varchar(50) |
| immunologie | Si positif, précisez | set('Arachide','Lait de vache','Blanc doeuf','Jaune doeuf','Poisson','Soja','Noisette','Autre') |
| immunologie | Si autre aliment, précisez | varchar(50) |
| immunologie | RAST arachide(valeur absolue) | varchar(50) |
| immunologie | RAST lait de vache(valeur absolue) | varchar(50) |
| immunologie | RAST blanc d'oeuf(valeur absolue) | varchar(50) |
| immunologie | RAST jaune d'oeuf(valeur absolue) | varchar(50) |
| immunologie | RAST poisson(valeur absolue) | varchar(50) |
| immunologie | RAST soja(valeur absolue) | varchar(50) |
| immunologie | RAST noisette(valeur absolue) | varchar(50) |
| immunologie | RAST autre aliment(valeur absolue) | varchar(50) |
| immunologie | Commentaire(s) | longtext |
| immunologie | **Animaux** | enum('Non renseigné','Non fait','Négatif','Positif') |
| immunologie | Si oui, précisez | set('Chat','Chien','Cheval','Blatte','Autre') |
| immunologie | Recherche de parasites | enum('Non renseigné','Faite','Non faite') |
| immunologie | Commentaire(s) | longtext |
| immunologie | Ascaridiose larvaire | enum('Non renseigné','Fait','Non fait') |
| immunologie | Résultat | enum('Non renseigné','Positif','Négatif') |
| immunologie | Valeur | varchar(50) |
| immunologie | Hydatidose | enum('Non renseigné','Fait','Non fait') |
| immunologie | Résultat | enum('Non renseigné','Positif','Négatif') |
| immunologie | Valeur | varchar(50) |
| immunologie | Toxocarose | enum('Non renseigné','Fait','Non fait') |
| immunologie | Résultat | enum('Non renseigné','Positif','Négatif') |
| immunologie | Valeur | varchar(50) |
| immunologie | Trichinellose | enum('Non renseigné','Fait','Non fait') |
| immunologie | Résultat | enum('Non renseigné','Positif','Négatif') |
| immunologie | Valeur | varchar(50) |
| immunologie | Autre | enum('Non renseigné','Fait','Non fait') |
| immunologie | *Si autre, préciser* | varchar(50) |
| immunologie | Résultat | enum('Non renseigné','Positif','Négatif') |
| immunologie | Valeur | varchar(50) |
| intolerance_medicamenteuse | ID_intolerance_medicamenteuse | |
| intolerance_medicamenteuse | ID_patient | int(10) unsigned |
| intolerance_medicamenteuse | **Allergies médicamenteuses** | enum('Non renseigné','Oui','Non') |
| intolerance_medicamenteuse | **Précisez** | longtext |
| intolerance_medicamenteuse | **Intolérances médicamenteuses** | enum('Non renseigné','Oui','Non') |
| intolerance_medicamenteuse | **Précisez** | longtext |
| lba | identifiant de lba |  |
| lba | identifiant de la fiche clinique | int(10) unsigned |
| lba | Germe 1 | varchar(50) |
| lba | Germe 2 | varchar(50) |
| lba | Germe 3 | varchar(50) |
| lba | Coproculture | enum('Non renseigné','Oui','Non') |
| lba | Autre Pseudomonas 1 | varchar(4) |
| lba | *Préciser* | varchar(50) |
| lba | Numération (UFC/ml) | enum('Non renseigné','10','10^2','10^3','10^4','10^5','10^6','10^7','10^8','10^9','10^10','10^11','10^12') |
| lba | Autre Pseudomonas 2 | varchar(4) |
| lba | *Préciser* | varchar(255) |
| lba | *Si sidérophages, richesse* | enum('Non renseigné','Nulle','Faible','Moyenne','Importante') |
| lba | Numération (UFC/ml) | enum('Non renseigné','10','10^2','10^3','10^4','10^5','10^6','10^7','10^8','10^9','10^10','10^11','10^12') |
| lba | Pseudomonas aeruginosa Ticarcilline R | varchar(4) |
| lba | Numération (UFC/ml) | enum('Non renseigné','10','10^2','10^3','10^4','10^5','10^6','10^7','10^8','10^9','10^10','10^11','10^12') |
| lba | Pseudomonas aeruginosa Ticarcilline S | varchar(4) |
| lba | Numération (UFC/ml) | enum('Non renseigné','10','10^2','10^3','10^4','10^5','10^6','10^7','10^8','10^9','10^10','10^11','10^12') |
| lba | Pseudomonas aeruginosa Ceftazidime R | varchar(255) |
| lba | Numération (UFC/ml) | enum('Non renseigné','10','10^2','10^3','10^4','10^5','10^6','10^7','10^8','10^9','10^10','10^11','10^12') |
| lba | Pseudomonas aeruginosa Ceftazidime S | varchar(255) |
| lba | Numération (UFC/ml) | enum('Non renseigné','10','10^2','10^3','10^4','10^5','10^6','10^7','10^8','10^9','10^10','10^11','10^12') |
| lba | Pseudomonas aeruginosa Imipenem R | varchar(255) |
| lba | Numération (UFC/ml) | enum('Non renseigné','10','10^2','10^3','10^4','10^5','10^6','10^7','10^8','10^9','10^10','10^11','10^12') |
| lba | Pseudomonas aeruginosa Imipenem S | varchar(4) |
| lba | Numération (UFC/ml) | enum('Non renseigné','10','10^2','10^3','10^4','10^5','10^6','10^7','10^8','10^9','10^10','10^11','10^12') |
| lba | Pseudomonas aeruginosa Tobramycine R | varchar(4) |
| lba | Numération (UFC/ml) | enum('Non renseigné','10','10^2','10^3','10^4','10^5','10^6','10^7','10^8','10^9','10^10','10^11','10^12') |
| lba | Pseudomonas aeruginosa Tobramycine S | varchar(4) |
| lba | Numération (UFC/ml) | enum('Non renseigné','10','10^2','10^3','10^4','10^5','10^6','10^7','10^8','10^9','10^10','10^11','10^12') |
| lba | Pseudomonas aeruginosa Ciprofloxacine R | varchar(4) |
| lba | Numération (UFC/ml) | enum('Non renseigné','10','10^2','10^3','10^4','10^5','10^6','10^7','10^8','10^9','10^10','10^11','10^12') |
| lba | Pseudomonas aeruginosa Ciprofloxacine S | varchar(4) |
| lba | Numération (UFC/ml) | enum('Non renseigné','10','10^2','10^3','10^4','10^5','10^6','10^7','10^8','10^9','10^10','10^11','10^12') |
| lba | **Burkholderia cepacia** | varchar(4) |
| lba | Numération (UFC/ml) | enum('Non renseigné','10','10^2','10^3','10^4','10^5','10^6','10^7','10^8','10^9','10^10','10^11','10^12') |
| lba | **Alcaligenes xylosoxidans** | varchar(4) |
| lba | Numération (UFC/ml) | enum('Non renseigné','10','10^2','10^3','10^4','10^5','10^6','10^7','10^8','10^9','10^10','10^11','10^12') |
| lba | **Commentaires** | longtext |
| lba | **Stenotrophomonas maltophilia** | varchar(4) |
| lba | Numération (UFC/ml) | enum('Non renseigné','10','10^2','10^3','10^4','10^5','10^6','10^7','10^8','10^9','10^10','10^11','10^12') |
| lba | *Si marqueurs T, préciser* | set('Cellules de Langerhans','CD4','CD8','CD3','Cellules NK','Autre') |
| lba | *Si marqueurs B, préciser* | set('CD19','Autre') |
| lba | *Si marqueurs divers, préciser* | set('CD45','Autre') |
| lba | Cellules de Langerhans : CD1a | enum('Non renseigné','Positif','Négatif') |
| lba | Si positif, pourcentage | varchar(50) |
| lba | Giemsa | enum('Non renseigné','Négatif','Faiblement positif','Fortement positif') |
| lba | Coloration de GROCOTT (et/ou GRAM WEIGERT) | varchar(50) |
| lba | Richesse en cellules bronchiques | enum('Non renseigné','Nulle','Faible','Moyenne','Importante') |
| lba | Date de l'examen | date |
| lba | **N° du lavage** | varchar(50) |
| lba | **Recherche de virus** | enum('Non renseigné','Oui','Non') |
| lba | **Méthode de recherche** | set('Culture','Immunofluorescence','Biologie moléculaire') |
| lba | **Virus retrouvé** | set('VRS','Influenzae','Parainfluenzae','Adenovirus','EBV','CMV','VIH','Autre') |
| lba | *Si autre(s) virus, précisez* | longtext |
| lba | **Flore** | enum('Non renseigné','Normale','Germes variés sans prédominance') |
| lba | Si germes variés, numération (UFC/ml) | enum('Non renseigné','10','10^2','10^3','10^4','10^5','10^6','10^7','10^8','10^9','10^10','10^11','10^12') |
| lba | **Mycobactéries** |  |
| lba | Mycobacterium tuberculosis | varchar(4) |
| lba | Numération (UFC/ml) | enum('Non renseigné','10','10^2','10^3','10^4','10^5','10^6','10^7','10^8','10^9','10^10','10^11','10^12') |
| lba | Mycobacteries atypiques | varchar(4) |
| lba | *Préciser* | varchar(50) |
| lba | Numération (UFC/ml) | enum('Non renseigné','10','10^2','10^3','10^4','10^5','10^6','10^7','10^8','10^9','10^10','10^11','10^12') |
| lba | Mycoplasme | enum('Non renseigné','Suspecté','Documenté') |
| lba | Chlamydiae | enum('Non renseigné','Suspecté','Documenté') |
| lba | Si documentée | enum('Non renseigné','Pneumoniae','Trachomatis','Psittaci') |
| lba | **Streptococcus pneumoniae** | varchar(4) |
| lba | Numération (UFC/ml) | enum('Non renseigné','10','10^2','10^3','10^4','10^5','10^6','10^7','10^8','10^9','10^10','10^11','10^12') |
| lba | Autre Streptococcus | varchar(4) |
| lba | Préciser | varchar(50) |
| lba | Numération (UFC/ml) | enum('Non renseigné','10','10^2','10^3','10^4','10^5','10^6','10^7','10^8','10^9','10^10','10^11','10^12') |
| lba | *Si autre(s) bactérie(s), précisez* | longtext |
| lba | **Haemophilus influenzae** | varchar(4) |
| lba | Numération (UFC/ml) | enum('Non renseigné','10','10^2','10^3','10^4','10^5','10^6','10^7','10^8','10^9','10^10','10^11','10^12') |
| lba | Haemophilus influenzae B-lactamase - | varchar(255) |
| lba | Numération (UFC/ml) | enum('Non renseigné','10','10^2','10^3','10^4','10^5','10^6','10^7','10^8','10^9','10^10','10^11','10^12') |
| lba | Haemophilus influenzae B-lactamase + | varchar(255) |
| lba | Numération (UFC/ml) | enum('Non renseigné','10','10^2','10^3','10^4','10^5','10^6','10^7','10^8','10^9','10^10','10^11','10^12') |
| lba | **Staphylococcus aureus** | varchar(50) |
| lba | Numération (UFC/ml) | enum('Non renseigné','10','10^2','10^3','10^4','10^5','10^6','10^7','10^8','10^9','10^10','10^11','10^12') |
| lba | Staphylococcus aureus Méti S | varchar(4) |
| lba | Numération (UFC/ml) | enum('Non renseigné','10','10^2','10^3','10^4','10^5','10^6','10^7','10^8','10^9','10^10','10^11','10^12') |
| lba | Staphylococcus aureus Méti R | varchar(4) |
| lba | Numération (UFC/ml) | enum('Non renseigné','10','10^2','10^3','10^4','10^5','10^6','10^7','10^8','10^9','10^10','10^11','10^12') |
| lba | Staphylococcus aureus Multirésistant | varchar(4) |
| lba | Numération (UFC/ml) | enum('Non renseigné','10','10^2','10^3','10^4','10^5','10^6','10^7','10^8','10^9','10^10','10^11','10^12') |
| lba | **Pseudomonas aeruginosa** | varchar(4) |
| lba | Numération (UFC/ml) | enum('Non renseigné','10','10^2','10^3','10^4','10^5','10^6','10^7','10^8','10^9','10^10','10^11','10^12') |
| lba | Type | enum('Non renseigné','Muqueux','Non muqueux') |
| lba | *Aspergillus fumigatus* | varchar(4) |
| lba | Numération (UFC/ml) | enum('Non renseigné','10','10^2','10^3','10^4','10^5','10^6','10^7','10^8','10^9','10^10','10^11','10^12') |
| lba | *Candida albicans* | varchar(4) |
| lba | Numération (UFC/ml) | enum('Non renseigné','10','10^2','10^3','10^4','10^5','10^6','10^7','10^8','10^9','10^10','10^11','10^12') |
| lba | *Pneumocystis jiroveci* | varchar(4) |
| lba | Numération (UFC/ml) | enum('Non renseigné','10','10^2','10^3','10^4','10^5','10^6','10^7','10^8','10^9','10^10','10^11','10^12') |
| lba | *Si autre(s) champignon(s), préciser* | longtext |
| lba | *Commentaire* | longtext |
| lba | Volume injecté | varchar(50) |
| lba | Volume recueilli | varchar(50) |
| lba | Volume examiné | varchar(50) |
| lba | **Aspects cytologiques** | enum('Non renseigné','Fait','Non fait') |
| lba | Macrophages | set('Spumeux','Multinucléés','Erythrophagocytoses','Pigmentés','Vacuoles lipidiques','Corps tingibles','Empoussiérés') |
| lba | Lymphocytes | set('Activés','Plasmocytoïdes','À grains azurophiles','Autre') |
| lba | Polynucléaires neutrophiles | set('Altérés','Germes','Pycnotiques') |
| lba | Polynucléaires éosinophiles : dystrophiques | enum('Non renseigné','Oui','Non') |
| lba | Cellules inhabituelles | enum('Non renseigné','Oui','Non') |
| lba | Si oui, préciser | set('Pneumocytes','Bronchiolaires','Cellules suspectes','Autre') |
| lba | *Si autre, préciser* | varchar(50) |
| lba | Matériel inhabituel | set('Agrégat lipoprotéique','Corps amylacés','Autre') |
| lba | Si autre, préciser | set('Corps ferrugineux','Corps étrangers','Membrane hyaline','Autre') |
| lba | *Préciser* | varchar(50) |
| lba | Aspect du lavage | enum('Non renseigné','Clair','Trouble','Lactescent','Hémorragique') |
| lba | **Coloration Oil Red O** | enum('Non renseigné','Fait','Non fait') |
| lba | Pourcentage de cellules positives | varchar(50) |
| lba | **Perls** | enum('Non renseigné','Fait','Non fait') |
| lba | Pourcentage | varchar(50) |
| lba | Score de Golde | varchar(50) |
| lba | **Immunocytochimie** | enum('Non renseigné','Fait','Non fait') |
| lba | CD4 (%/nb lymphocytes) | varchar(50) |
| lba | CD8 (%/nb lymphocytes) | varchar(50) |
| lba | CD1a (%/nb macrophages) | varchar(50) |
| lba | CD4+/CD8+ | varchar(50) |
| lba | *Autre* | varchar(50) |
| lba | Nombre de cellules / ml | varchar(50) |
| lba | **Immunophénotypage** | enum('Non renseigné','Fait','Non fait') |
| lba | Cytométrie de flux (CMF) | enum('Non renseigné','Oui','Non') |
| lba | **Conclusion** | longtext |
| lba | Contamination | enum('Non renseigné','Oui','Non') |
| lba | Hématies | enum('Non renseigné','Nulle','Faible','Moyenne','Importante') |
| lba | **Formule** | enum('Non renseigné','Faite','Non faite') |
| lba | Macrophages (%) | varchar(50) |
| lba | Lymphocytes (%) | varchar(50) |
| lba | Polynucléaires neutrophiles (%) | varchar(50) |
| lba | Polynucléaires eosinophiles (%) | varchar(50) |
| lba | Mastocytes (%) | varchar(50) |
| lba | **Analyse du profil des protéines du surfactant** | enum('Non renseigné','Faite','Non faite') |
| lba | SP-B (8 kDa) | enum('Non renseigné','Positif','Négatif') |
| lba | Commentaire | varchar(50) |
| lba | Pro SP-C | enum('Non renseigné','Oui','Non') |
| lba | Pro SP-C (24-26 kDa) | enum('Non renseigné','Positif','Négatif') |
| lba | Commentaire | varchar(50) |
| lba | Pro SP-C (16 kDa) | enum('Non renseigné','Positif','Négatif') |
| lba | Commentaire | varchar(50) |
| lba | Pro SP-C (14 kDa) | enum('Non renseigné','Positif','Négatif') |
| lba | Commentaire | varchar(50) |
| lba | SPC | enum('Non renseigné','Positif','Négatif') |
| lba | Commentaire | varchar(50) |
| lba | **Matériel congelé** | set('Surnageant','Cellules') |
| lba | **Conclusion** | longtext |
| lba | Pro SP-B | enum('Non renseigné','Oui','Non') |
| lba | Pro SP-B (25-26 kDa) | enum('Non renseigné','Positif','Négatif') |
| lba | Commentaire | varchar(50) |
| lba | Pro SP-B (19-21 kDa) | enum('Non renseigné','Positif','Négatif') |
| lba | Commentaire | varchar(50) |
| lba | Pro SP-B (15 kDa) | enum('Non renseigné','Positif','Négatif') |
| lba | Commentaire | varchar(50) |
| lba | SP-B | enum('Non renseigné','Oui','Non') |
| lba_therapeutiq | identifiant de lba_therapeutiq |  |
| lba_therapeutiq | ID_fiche_clinique | int(10) unsigned |
| lba_therapeutiq | **LBA thérapeutique poumon droit** | enum('Non renseigné','Oui','Non') |
| lba_therapeutiq | Si oui, nombre | enum('Non renseigné','1','2','3') |
| lba_therapeutiq | Date du 1er LBA | date |
| lba_therapeutiq | Volume du 1er LBA (ml) | varchar(50) |
| lba_therapeutiq | Ventilation au décours 1er LBA | enum('Non renseigné','Oui','Non') |
| lba_therapeutiq | Si oui, durée | varchar(50) |
| lba_therapeutiq |  | enum('Non renseigné','jour(s)','heure(s)') |
| lba_therapeutiq | Date du 2ème LBA | date |
| lba_therapeutiq | Volume du 2ème LBA (ml) | varchar(50) |
| lba_therapeutiq | Ventilation au décours 2ème LBA | enum('Non renseigné','Oui','Non') |
| lba_therapeutiq | Si oui, durée | varchar(50) |
| lba_therapeutiq |  | enum('Non renseigné','jour(s)','heure(s)') |
| lba_therapeutiq | Date du 3ème LBA | date |
| lba_therapeutiq | Volume du 3ème LBA (ml) | varchar(50) |
| lba_therapeutiq | Ventilation au décours 3ème LBA | enum('Non renseigné','Oui','Non') |
| lba_therapeutiq | Si oui, durée | varchar(50) |
| lba_therapeutiq |  | enum('Non renseigné','jour(s)','heure(s)') |
| lba_therapeutiq | Date du 1er LBA | date |
| lba_therapeutiq | Volume du 1er LBA (ml) | varchar(50) |
| lba_therapeutiq | Ventilation au décours 1er LBA | enum('Non renseigné','Oui','Non') |
| lba_therapeutiq | Si oui, durée | varchar(50) |
| lba_therapeutiq |  | enum('Non renseigné','jour(s)','heure(s)') |
| lba_therapeutiq | Date du 2ème LBA | date |
| lba_therapeutiq | Volume du 2ème LBA (ml) | varchar(50) |
| lba_therapeutiq | Ventilation au décours 2ème LBA | enum('Non renseigné','Oui','Non') |
| lba_therapeutiq | Si oui, durée | varchar(50) |
| lba_therapeutiq |  | enum('Non renseigné','jour(s)','heure(s)') |
| lba_therapeutiq | Date du 3ème LBA | date |
| lba_therapeutiq | Volume du 3ème LBA (ml) | varchar(50) |
| lba_therapeutiq | Ventilation au décours 3ème LBA | enum('Non renseigné','Oui','Non') |
| lba_therapeutiq | Si oui, durée | varchar(50) |
| lba_therapeutiq |  | enum('Non renseigné','jour(s)','heure(s)') |
| lba_therapeutiq | **LBA thérapeutique poumon gauche** | enum('Non renseigné','Oui','Non') |
| lba_therapeutiq | Si oui, nombre | enum('Non renseigné','1','2','3 ou +') |
| medicament | identifiant de medicament |  |
| medicament | Catégorie | int(50) |
| medicament | Nom du médicament | varchar(50) |
| medicament | Unité de prescription du médicament (cachets/jour, ...) | varchar(255) |
| medicament | Commentaire par défaut | longtext |
| medicament | Pied de page | longtext |
| medicament_categorie | identifiant de medicament_categorie | |
| medicament_categorie | Nom de la catégorie | varchar(50) |
| microbiologie | identifiant de microbiologie |  |
| microbiologie | identifiant de la fiche clinique | int(10) unsigned |
| microbiologie | Germe 2 | varchar(50) |
| microbiologie | Germe 3 | varchar(50) |
| microbiologie | Autre Pseudomonas 1 | varchar(4) |
| microbiologie | *Préciser* | varchar(50) |
| microbiologie | Numération (UFC/ml) | enum('Non renseigné','10','10^2','10^3','10^4','10^5','10^6','10^7','10^8','10^9','10^10','10^11','10^12') |
| microbiologie | Autre Pseudomonas 2 | varchar(4) |
| microbiologie | *Préciser* | varchar(255) |
| microbiologie | Numération (UFC/ml) | enum('Non renseigné','10','10^2','10^3','10^4','10^5','10^6','10^7','10^8','10^9','10^10','10^11','10^12') |
| microbiologie | Pseudomonas aeruginosa Ticarcilline R | varchar(4) |
| microbiologie | Numération (UFC/ml) | enum('Non renseigné','10','10^2','10^3','10^4','10^5','10^6','10^7','10^8','10^9','10^10','10^11','10^12') |
| microbiologie | Pseudomonas aeruginosa Ticarcilline S | varchar(4) |
| microbiologie | Numération (UFC/ml) | enum('Non renseigné','10','10^2','10^3','10^4','10^5','10^6','10^7','10^8','10^9','10^10','10^11','10^12') |
| microbiologie | Pseudomonas aeruginosa Ceftazidime R | varchar(255) |
| microbiologie | Numération (UFC/ml) | enum('Non renseigné','10','10^2','10^3','10^4','10^5','10^6','10^7','10^8','10^9','10^10','10^11','10^12') |
| microbiologie | Pseudomonas aeruginosa Ceftazidime S | varchar(255) |
| microbiologie | Numération (UFC/ml) | enum('Non renseigné','10','10^2','10^3','10^4','10^5','10^6','10^7','10^8','10^9','10^10','10^11','10^12') |
| microbiologie | Pseudomonas aeruginosa Imipenem R | varchar(255) |
| microbiologie | Numération (UFC/ml) | enum('Non renseigné','10','10^2','10^3','10^4','10^5','10^6','10^7','10^8','10^9','10^10','10^11','10^12') |
| microbiologie | Pseudomonas aeruginosa Imipenem S | varchar(4) |
| microbiologie | Numération (UFC/ml) | enum('Non renseigné','10','10^2','10^3','10^4','10^5','10^6','10^7','10^8','10^9','10^10','10^11','10^12') |
| microbiologie | Pseudomonas aeruginosa Tobramycine R | varchar(4) |
| microbiologie | Numération (UFC/ml) | enum('Non renseigné','10','10^2','10^3','10^4','10^5','10^6','10^7','10^8','10^9','10^10','10^11','10^12') |
| microbiologie | Pseudomonas aeruginosa Tobramycine S | varchar(4) |
| microbiologie | Numération (UFC/ml) | enum('Non renseigné','10','10^2','10^3','10^4','10^5','10^6','10^7','10^8','10^9','10^10','10^11','10^12') |
| microbiologie | Pseudomonas aeruginosa Ciprofloxacine R | varchar(4) |
| microbiologie | Numération (UFC/ml) | enum('Non renseigné','10','10^2','10^3','10^4','10^5','10^6','10^7','10^8','10^9','10^10','10^11','10^12') |
| microbiologie | Pseudomonas aeruginosa Ciprofloxacine S | varchar(4) |
| microbiologie | Numération (UFC/ml) | enum('Non renseigné','10','10^2','10^3','10^4','10^5','10^6','10^7','10^8','10^9','10^10','10^11','10^12') |
| microbiologie | **Burkholderia cepacia** | varchar(4) |
| microbiologie | Numération (UFC/ml) | enum('Non renseigné','10','10^2','10^3','10^4','10^5','10^6','10^7','10^8','10^9','10^10','10^11','10^12') |
| microbiologie | **Alcaligenes xylosoxidans** | varchar(4) |
| microbiologie | **Commentaires** | longtext |
| microbiologie | **Mode de prélèvement** | set('Aspiration nasale','Autre') |
| microbiologie | *Si autre mode de prélèvement, précisez* | longtext |
| microbiologie | Date de l'examen | date |
| microbiologie | Germes retrouvés | longtext |
| microbiologie | **Commentaire libre** | longtext |
| microbiologie | Hémoculture | enum('Non renseigné','Oui','Non') |
| microbiologie | ECBU | enum('Non renseigné','Oui','Non') |
| microbiologie | Coproculture | enum('Non renseigné','Oui','Non') |
| microbiologie | Virus retrouvé | set('VRS','Influenzae','Parainfluenzae','Adenovirus','EBV','CMV','VIH','Autre') |
| microbiologie | *Si autre(s) virus, précisez* | longtext |
| microbiologie | **Recherche de virus** | enum('Non renseigné','Oui','Non') |
| microbiologie | Méthode de recherche | set('Culture','Immunofluorescence','Biologie moléculaire') |
| microbiologie | Résultat | enum('Non renseigné','Positif','Négatif','Non interprétable') |
| microbiologie | **Flore** | enum('Non renseigné','Normale','Germes variés sans prédominance') |
| microbiologie | Si germes variés, numération (UFC/ml) | enum('Non renseigné','10','10^2','10^3','10^4','10^5','10^6','10^7','10^8','10^9','10^10','10^11','10^12') |
| microbiologie | **Mycobactéries** |  |
| microbiologie | Mycobacterium tuberculosis | varchar(4) |
| microbiologie | Numération (UFC/ml) | enum('Non renseigné','10','10^2','10^3','10^4','10^5','10^6','10^7','10^8','10^9','10^10','10^11','10^12') |
| microbiologie | Mycobacteries atypiques | varchar(4) |
| microbiologie | *Préciser* | varchar(50) |
| microbiologie | Numération (UFC/ml) | enum('Non renseigné','10','10^2','10^3','10^4','10^5','10^6','10^7','10^8','10^9','10^10','10^11','10^12') |
| microbiologie | Mycoplasme | enum('Non renseigné','Suspecté','Documenté') |
| microbiologie | Chlamydiae | enum('Non renseigné','Suspecté','Documenté') |
| microbiologie | Si documentée | enum('Non renseigné','Pneumoniae','Trachomatis','Psittaci') |
| microbiologie | **Streptococcus pneumoniae** | varchar(4) |
| microbiologie | Numération (UFC/ml) | enum('Non renseigné','10','10^2','10^3','10^4','10^5','10^6','10^7','10^8','10^9','10^10','10^11','10^12') |
| microbiologie | Autre Streptococcus | varchar(4) |
| microbiologie | Préciser | varchar(50) |
| microbiologie | Numération (UFC/ml) | enum('Non renseigné','10','10^2','10^3','10^4','10^5','10^6','10^7','10^8','10^9','10^10','10^11','10^12') |
| microbiologie | *Si autre(s) bactérie(s), précisez* | longtext |
| microbiologie | **Commentaire(s)** | longtext |
| microbiologie | **Haemophilus influenzae** | varchar(4) |
| microbiologie | Numération (UFC/ml) | enum('Non renseigné','10','10^2','10^3','10^4','10^5','10^6','10^7','10^8','10^9','10^10','10^11','10^12') |
| microbiologie | Haemophilus influenzae B-lactamase - | varchar(255) |
| microbiologie | Numération (UFC/ml) | enum('Non renseigné','10','10^2','10^3','10^4','10^5','10^6','10^7','10^8','10^9','10^10','10^11','10^12') |
| microbiologie | Haemophilus influenzae B-lactamase + | varchar(255) |
| microbiologie | Numération (UFC/ml) | enum('Non renseigné','10','10^2','10^3','10^4','10^5','10^6','10^7','10^8','10^9','10^10','10^11','10^12') |
| microbiologie | **Staphylococcus aureus** | varchar(50) |
| microbiologie | Numération (UFC/ml) | enum('Non renseigné','10','10^2','10^3','10^4','10^5','10^6','10^7','10^8','10^9','10^10','10^11','10^12') |
| microbiologie | Staphylococcus aureus Méti S | varchar(4) |
| microbiologie | Numération (UFC/ml) | enum('Non renseigné','10','10^2','10^3','10^4','10^5','10^6','10^7','10^8','10^9','10^10','10^11','10^12') |
| microbiologie | Staphylococcus aureus Méti R | varchar(4) |
| microbiologie | Numération (UFC/ml) | enum('Non renseigné','10','10^2','10^3','10^4','10^5','10^6','10^7','10^8','10^9','10^10','10^11','10^12') |
| microbiologie | Staphylococcus aureus Multirésistant | varchar(4) |
| microbiologie | Numération (UFC/ml) | enum('Non renseigné','10','10^2','10^3','10^4','10^5','10^6','10^7','10^8','10^9','10^10','10^11','10^12') |
| microbiologie | **Pseudomonas aeruginosa** | varchar(4) |
| microbiologie | Numération (UFC/ml) | enum('Non renseigné','10','10^2','10^3','10^4','10^5','10^6','10^7','10^8','10^9','10^10','10^11','10^12') |
| microbiologie | Type | enum('Non renseigné','Muqueux','Non muqueux') |
| microbiologie | *Aspergillus fumigatus* | varchar(4) |
| microbiologie | Numération (UFC/ml) | enum('Non renseigné','10','10^2','10^3','10^4','10^5','10^6','10^7','10^8','10^9','10^10','10^11','10^12') |
| microbiologie | *Candida albicans* | varchar(4) |
| microbiologie | Numération (UFC/ml) | enum('Non renseigné','10','10^2','10^3','10^4','10^5','10^6','10^7','10^8','10^9','10^10','10^11','10^12') |
| microbiologie | *Pneumocystis jiroveci* | varchar(4) |
| microbiologie | Numération (UFC/ml) | enum('Non renseigné','10','10^2','10^3','10^4','10^5','10^6','10^7','10^8','10^9','10^10','10^11','10^12') |
| microbiologie | *Si autre(s) champignon(s), préciser* | longtext |
| microbiologie | Traitement prescrit | enum('Non renseigné','Oui','Non') |
| microbiologie | *Si oui, préciser* | longtext |
| patient | Nom | varchar(100) |
| patient | Prénom | varchar(100) |
| patient | Nom marital | varchar(50) |
| patient | Dossier du patient | enum('Actif','Inactif') |
| patient | Si inactif, situation | enum('Non renseigné','Perdu de vue','Décédé') |
| patient | Statut | enum('Non renseigné','Non applicable','Célibataire','Marié','Divorcé','Veuf') |
| patient | Enfants | enum('Non renseigné','Non applicable','Oui','Non') |
| patient | Nombre d'enfants du patient | varchar(50) |
| patient | **Date d'inclusion dans e-Pi** | date |
| patient | Sexe | enum('Féminin','Masculin') |
| patient | Date de naissance | date |
| patient | Lieu de naissance | varchar(50) |
| patient | Code postal de naissance (ou pays si patient étranger) | varchar(10) |
| patient | Code patient hôpital | varchar(100) |
| patient | Nom du centre de compétence ou de référence | int(10) unsigned |
| patient | Nom du centre d'origine | varchar(50) |
| patient | Nom du centre de suivi | int(10) unsigned |
| patient | **Coordonnées du patient** |  |
| patient | **Coordonnées de la mère** |  |
| patient | **Coordonnées du père** |  |
| patient | Téléphone fixe du patient | varchar(20) |
| patient | Téléphone portable du patient | varchar(20) |
| patient | e-mail du patient | varchar(50) |
| patient | Téléphone fixe de la mère | varchar(50) |
| patient | Téléphone portable de la mère | varchar(20) |
| patient | e-mail de la mère | varchar(50) |
| patient | Téléphone fixe du père | varchar(50) |
| patient | Téléphone portable du père | varchar(20) |
| patient | e-mail du père | varchar(50) |
| patient | **Coordonnées du conjoint** |  |
| patient | Téléphone fixe du conjoint | varchar(50) |
| patient | Téléphone portable du conjoint | varchar(50) |
| patient | e-mail du conjoint | varchar(50) |
| patient | ID_patient |  |
| patient | postit | longtext |
| patient | Partage des données avec un autre centre | int(10) unsigned |
| patient | Nom de la ville où le patient est suivi | varchar(50) |
| patient | Btiment | varchar(50) |
| patient | Numéro | varchar(50) |
| patient | Rue | varchar(60) |
| patient | Code postal | varchar(20) |
| patient | Ville | varchar(60) |
| patient | Pays | int(11) |
| patient | Si autre pays, préciser | varchar(50) |
| patient | **Activité du patient** |  |
| patient | **Activité de la mère** |  |
| patient | **Activité du père** |  |
| patient | Patient en activité | enum('Non renseigné','Non applicable','Oui','Non') |
| patient | Si oui, type d'activité | set('Scolaire','Professionnelle') |
| patient | Scolarité (classe) ou activité professionnelle | varchar(50) |
| patient | En activité | enum('Non renseigné','Oui','Non') |
| patient | Profession de la mère | varchar(50) |
| patient | En activité | enum('Non renseigné','Oui','Non') |
| patient | Profession du père | varchar(50) |
| patient | Btiment | varchar(50) |
| patient | **Préciser** | varchar(50) |
| patient | Numéro | varchar(50) |
| patient | Rue | varchar(60) |
| patient | Code postal | varchar(20) |
| patient | Ville | varchar(60) |
| patient | Pays | int(11) |
| patient | Si autre pays, préciser | varchar(50) |
| patient_carnet_d_adresses | identificateur du patient_carnet d'adresses | |
| patient_carnet_d_adresses | Patient | int(10) unsigned |
| patient_carnet_d_adresses | Professionel | int(10) unsigned |
| patient_carnet_d_adresses | Nature des soins | set('Médecin hospitalier','Médecin traitant','Médecin référent') |
| patient_societe | indicateur patient_société |  |
| patient_societe | Date de début des soins | date |
| patient_societe | Date de fin des soins | date |
| patient_societe | Patient | int(10) unsigned |
| patient_societe | Société | int(10) unsigned |
| personne_a_contacter | Patient | int(10) unsigned |
| personne_a_contacter | ID_personne_a_contacter |  |
| personne_a_contacter | Titre | enum('Madame','Madame et Monsieur','Mademoiselle','Monsieur') |
| personne_a_contacter | Nom | varchar(80) |
| personne_a_contacter | Prénom | varchar(80) |
| personne_a_contacter | Lien de parenté | enum('père','mère','parents','oncle','tante','soeur','frère','grand-père','grand-mère','tuteur','conjoint') |
| personne_a_contacter | Btiment | varchar(50) |
| personne_a_contacter | Numéro | varchar(50) |
| personne_a_contacter | Rue | varchar(60) |
| personne_a_contacter | Code postal | varchar(20) |
| personne_a_contacter | Ville | varchar(60) |
| personne_a_contacter | Pays | int(11) |
| personne_a_contacter | Si autre pays, préciser | varchar(50) |
| personne_a_contacter | Téléphone du domicile | varchar(100) |
| personne_a_contacter | Téléphone professionnel 1 | varchar(100) |
| personne_a_contacter | Téléphone portable 1 | varchar(100) |
| personne_a_contacter | Téléphone professionnel 2 | varchar(100) |
| personne_a_contacter | Téléphone portable 2 | varchar(100) |
| personne_a_contacter | e-mail | varchar(50) |
| plevre | identifiant de plevre |  |
| plevre | identifiant de la fiche clinique | int(10) unsigned |
| plevre | Germe 1 | varchar(50) |
| plevre | Germe 2 | varchar(50) |
| plevre | Germe 3 | varchar(50) |
| plevre | Coproculture | enum('Non renseigné','Oui','Non') |
| plevre | Autre Pseudomonas 1 | varchar(4) |
| plevre | *Préciser* | varchar(50) |
| plevre | Numération (UFC/ml) | enum('Non renseigné','10','10^2','10^3','10^4','10^5','10^6','10^7','10^8','10^9','10^10','10^11','10^12') |
| plevre | Autre Pseudomonas 2 | varchar(4) |
| plevre | *Préciser* | varchar(255) |
| plevre | Numération (UFC/ml) | enum('Non renseigné','10','10^2','10^3','10^4','10^5','10^6','10^7','10^8','10^9','10^10','10^11','10^12') |
| plevre | Pseudomonas aeruginosa Ticarcilline R | varchar(4) |
| plevre | Numération (UFC/ml) | enum('Non renseigné','10','10^2','10^3','10^4','10^5','10^6','10^7','10^8','10^9','10^10','10^11','10^12') |
| plevre | Pseudomonas aeruginosa Ticarcilline S | varchar(4) |
| plevre | Numération (UFC/ml) | enum('Non renseigné','10','10^2','10^3','10^4','10^5','10^6','10^7','10^8','10^9','10^10','10^11','10^12') |
| plevre | Pseudomonas aeruginosa Ceftazidime R | varchar(255) |
| plevre | Numération (UFC/ml) | enum('Non renseigné','10','10^2','10^3','10^4','10^5','10^6','10^7','10^8','10^9','10^10','10^11','10^12') |
| plevre | Pseudomonas aeruginosa Ceftazidime S | varchar(255) |
| plevre | Numération (UFC/ml) | enum('Non renseigné','10','10^2','10^3','10^4','10^5','10^6','10^7','10^8','10^9','10^10','10^11','10^12') |
| plevre | Pseudomonas aeruginosa Imipenem R | varchar(255) |
| plevre | Numération (UFC/ml) | enum('Non renseigné','10','10^2','10^3','10^4','10^5','10^6','10^7','10^8','10^9','10^10','10^11','10^12') |
| plevre | Pseudomonas aeruginosa Imipenem S | varchar(4) |
| plevre | Numération (UFC/ml) | enum('Non renseigné','10','10^2','10^3','10^4','10^5','10^6','10^7','10^8','10^9','10^10','10^11','10^12') |
| plevre | Pseudomonas aeruginosa Tobramycine R | varchar(4) |
| plevre | Numération (UFC/ml) | enum('Non renseigné','10','10^2','10^3','10^4','10^5','10^6','10^7','10^8','10^9','10^10','10^11','10^12') |
| plevre | Pseudomonas aeruginosa Tobramycine S | varchar(4) |
| plevre | Numération (UFC/ml) | enum('Non renseigné','10','10^2','10^3','10^4','10^5','10^6','10^7','10^8','10^9','10^10','10^11','10^12') |
| plevre | Pseudomonas aeruginosa Ciprofloxacine R | varchar(4) |
| plevre | Numération (UFC/ml) | enum('Non renseigné','10','10^2','10^3','10^4','10^5','10^6','10^7','10^8','10^9','10^10','10^11','10^12') |
| plevre | Pseudomonas aeruginosa Ciprofloxacine S | varchar(4) |
| plevre | Numération (UFC/ml) | enum('Non renseigné','10','10^2','10^3','10^4','10^5','10^6','10^7','10^8','10^9','10^10','10^11','10^12') |
| plevre | **Burkholderia cepacia** | varchar(4) |
| plevre | Numération (UFC/ml) | enum('Non renseigné','10','10^2','10^3','10^4','10^5','10^6','10^7','10^8','10^9','10^10','10^11','10^12') |
| plevre | **Alcaligenes xylosoxidans** | varchar(4) |
| plevre | Numération (UFC/ml) | enum('Non renseigné','10','10^2','10^3','10^4','10^5','10^6','10^7','10^8','10^9','10^10','10^11','10^12') |
| plevre | **Commentaires** | longtext |
| plevre | **Stenotrophomonas maltophilia** | varchar(4) |
| plevre | Numération (UFC/ml) | enum('Non renseigné','10','10^2','10^3','10^4','10^5','10^6','10^7','10^8','10^9','10^10','10^11','10^12') |
| plevre | Date de l'examen | date |
| plevre | **Recherche de virus** | enum('Non renseigné','Oui','Non') |
| plevre | **Méthode de recherche** | set('Culture','Immunofluorescence','Biologie moléculaire') |
| plevre | **Virus retrouvé** | set('VRS','Influenzae','Parainfluenzae','Adenovirus','EBV','CMV','VIH','Autre') |
| plevre | *Si autre(s) virus, précisez* | longtext |
| plevre | Ag solubles | enum('Non renseigné','Positif','Négatif') |
| plevre | **Flore** | enum('Non renseigné','Normale','Germes variés sans prédominance') |
| plevre | Si germes variés, numération (UFC/ml) | enum('Non renseigné','10','10^2','10^3','10^4','10^5','10^6','10^7','10^8','10^9','10^10','10^11','10^12') |
| plevre | Streptocoque B | varchar(4) |
| plevre | Numération (UFC/ml) | enum('Non renseigné','10','10^2','10^3','10^4','10^5','10^6','10^7','10^8','10^9','10^10','10^11','10^12') |
| plevre | E-coli K1 | varchar(4) |
| plevre | Numération (UFC/ml) | enum('Non renseigné','10','10^2','10^3','10^4','10^5','10^6','10^7','10^8','10^9','10^10','10^11','10^12') |
| plevre | Meningocoque B | varchar(4) |
| plevre | Numération (UFC/ml) | enum('Non renseigné','10','10^2','10^3','10^4','10^5','10^6','10^7','10^8','10^9','10^10','10^11','10^12') |
| plevre | Meningocoque A | varchar(4) |
| plevre | Numération (UFC/ml) | enum('Non renseigné','10','10^2','10^3','10^4','10^5','10^6','10^7','10^8','10^9','10^10','10^11','10^12') |
| plevre | Meningocoque C | varchar(4) |
| plevre | Numération (UFC/ml) | enum('Non renseigné','10','10^2','10^3','10^4','10^5','10^6','10^7','10^8','10^9','10^10','10^11','10^12') |
| plevre | Meningocoque YW135 | varchar(4) |
| plevre | Numération (UFC/ml) | enum('Non renseigné','10','10^2','10^3','10^4','10^5','10^6','10^7','10^8','10^9','10^10','10^11','10^12') |
| plevre | **Mycobactéries** |  |
| plevre | Mycobacterium tuberculosis | varchar(4) |
| plevre | Numération (UFC/ml) | enum('Non renseigné','10','10^2','10^3','10^4','10^5','10^6','10^7','10^8','10^9','10^10','10^11','10^12') |
| plevre | Mycobacteries atypiques | varchar(4) |
| plevre | *Préciser* | varchar(50) |
| plevre | Numération (UFC/ml) | enum('Non renseigné','10','10^2','10^3','10^4','10^5','10^6','10^7','10^8','10^9','10^10','10^11','10^12') |
| plevre | Mycoplasme | enum('Non renseigné','Suspecté','Documenté') |
| plevre | Chlamydiae | enum('Non renseigné','Suspecté','Documenté') |
| plevre | Si documentée | enum('Non renseigné','Pneumoniae','Trachomatis','Psittaci') |
| plevre | **Streptococcus pneumoniae** | varchar(4) |
| plevre | Numération (UFC/ml) | enum('Non renseigné','10','10^2','10^3','10^4','10^5','10^6','10^7','10^8','10^9','10^10','10^11','10^12') |
| plevre | Autre Streptococcus | varchar(4) |
| plevre | Préciser | varchar(50) |
| plevre | Numération (UFC/ml) | enum('Non renseigné','10','10^2','10^3','10^4','10^5','10^6','10^7','10^8','10^9','10^10','10^11','10^12') |
| plevre | *Si autre(s) bactérie(s), précisez* | longtext |
| plevre | **Haemophilus influenzae** | varchar(4) |
| plevre | Numération (UFC/ml) | enum('Non renseigné','10','10^2','10^3','10^4','10^5','10^6','10^7','10^8','10^9','10^10','10^11','10^12') |
| plevre | Haemophilus influenzae B-lactamase - | varchar(255) |
| plevre | Numération (UFC/ml) | enum('Non renseigné','10','10^2','10^3','10^4','10^5','10^6','10^7','10^8','10^9','10^10','10^11','10^12') |
| plevre | Haemophilus influenzae B-lactamase + | varchar(255) |
| plevre | Numération (UFC/ml) | enum('Non renseigné','10','10^2','10^3','10^4','10^5','10^6','10^7','10^8','10^9','10^10','10^11','10^12') |
| plevre | **Staphylococcus aureus** | varchar(50) |
| plevre | Numération (UFC/ml) | enum('Non renseigné','10','10^2','10^3','10^4','10^5','10^6','10^7','10^8','10^9','10^10','10^11','10^12') |
| plevre | Staphylococcus aureus Méti S | varchar(4) |
| plevre | Numération (UFC/ml) | enum('Non renseigné','10','10^2','10^3','10^4','10^5','10^6','10^7','10^8','10^9','10^10','10^11','10^12') |
| plevre | Staphylococcus aureus Méti R | varchar(4) |
| plevre | Numération (UFC/ml) | enum('Non renseigné','10','10^2','10^3','10^4','10^5','10^6','10^7','10^8','10^9','10^10','10^11','10^12') |
| plevre | Staphylococcus aureus Multirésistant | varchar(4) |
| plevre | Numération (UFC/ml) | enum('Non renseigné','10','10^2','10^3','10^4','10^5','10^6','10^7','10^8','10^9','10^10','10^11','10^12') |
| plevre | **Pseudomonas aeruginosa** | varchar(4) |
| plevre | Numération (UFC/ml) | enum('Non renseigné','10','10^2','10^3','10^4','10^5','10^6','10^7','10^8','10^9','10^10','10^11','10^12') |
| plevre | Type | enum('Non renseigné','Muqueux','Non muqueux') |
| plevre | *Aspergillus fumigatus* | varchar(4) |
| plevre | Numération (UFC/ml) | enum('Non renseigné','10','10^2','10^3','10^4','10^5','10^6','10^7','10^8','10^9','10^10','10^11','10^12') |
| plevre | *Candida albicans* | varchar(4) |
| plevre | Numération (UFC/ml) | enum('Non renseigné','10','10^2','10^3','10^4','10^5','10^6','10^7','10^8','10^9','10^10','10^11','10^12') |
| plevre | *Pneumocystis jiroveci* | varchar(4) |
| plevre | Numération (UFC/ml) | enum('Non renseigné','10','10^2','10^3','10^4','10^5','10^6','10^7','10^8','10^9','10^10','10^11','10^12') |
| plevre | *Si autre(s) champignon(s), préciser* | longtext |
| plevre | *Commentaire* | longtext |
| plevre | **Aspect du liquide** | set('Citrin','Purulent','Hémorragique','Lactescent') |
| plevre | **Conclusion** | longtext |
| plevre | Nombre de cellules / ml | varchar(50) |
| plevre | Type de cellules prédominantes | varchar(255) |
| plevre | **Présence de cellules anormales** | enum('Non renseigné','Oui','Non') |
| plevre | Si oui, précisez lesquelles | longtext |
| plevre | Commentaires | longtext |
| plevre | Protides (g/l) | varchar(50) |
| plevre | Glucose (g/l) | varchar(50) |
| plevre | Triglycérides (g/l) | varchar(50) |
| plevre | Chylomicrons | varchar(50) |
| plevre | pH | varchar(50) |
| plevre | Amylase (UI/l) | varchar(50) |
| plevre | LDH (UI/l) | varchar(50) |
| plevre | Autres | longtext |
| postit | identifiant de postit |  |
| postit | Identifiant du patient | int(11) |
| postit | Post-it | longtext |
| prescription | identifiant de prescription |  |
| prescription | Ordonnance | int(50) |
| prescription | Médicament | int(50) |
| prescription | Quantité prescrite | varchar(150) |
| prescription | Durée | varchar(50) |
| prescription | Mode d'administration | varchar(150) |
| prescription | Commentaire | longtext |
| prescription | Traitement non exonéré | varchar(5) |
| protocole | identifiant de protocole |  |
| protocole | ID_patient | int(10) unsigned |
| protocole | Date d'inclusion dans le protocole | date |
| protocole | Date de sortie du protocole | date |
| protocole | Inclusion du patient dans un protocole d'investigation clinique | enum('Non renseigné','Oui','Non') |
| protocole | Date d'inclusion dans le protocole | date |
| protocole | Date de sortie du protocole | date |
| protocole | **Commentaire(s)** | longtext |
| protocole | Nom du protocole | set('PHRC 2007 APSE : "Anomalies du surfactant"','Autre') |
| protocole | Date d'inclusion dans le protocole APSE | date |
| protocole | Date de sortie du protocole APSE | date |
| protocole | N° de centre (APSE) | varchar(50) |
| protocole | N° d'inclusion (APSE) | varchar(50) |
| protocole | Signature du consentement (APSE) | enum('Non renseigné','Oui','Non') |
| protocole | Si oui, par qui | set('Père','Mère','Patient') |
| protocole | Commentaire(s) APSE | longtext |
| societe | id_societe |  |
| societe | Nom | varchar(20) |
| societe | Type de société | int(10) unsigned |
| societe | Btiment | varchar(50) |
| societe | Numéro | varchar(50) |
| societe | Rue | varchar(60) |
| societe | Code postal | varchar(20) |
| societe | Ville | varchar(60) |
| societe | Pays | int(11) |
| societe | Si autre pays, préciser | varchar(50) |
| societe | Téléphone de la société | varchar(20) |
| societe | Téléphone portable | varchar(20) |
| societe | Numéro de fax | varchar(20) |
| traitement | identifiant de traitement |  |
| traitement | Fiche clinique | int(10) unsigned |
| traitement | Durée | smallint(4) |
| traitement | Nom | varchar(50) |
| vaccinations | identifiant de vaccinations |  |
| vaccinations | clé étrangère patient | int(10) unsigned |
| vaccinations | **BCG (à partir de 1 mois)** | enum('Non renseigné','Oui','Non') |
| vaccinations | Date | date |
| vaccinations | **DTpolio, Haemophilus, Coqueluche (INFANRIX QUINTA/PENTAVAC/PENTACOQ)** | enum('Non renseigné','Oui','Non') |
| vaccinations | Injection 1 (1 mois) Date | date |
| vaccinations | Injection 2 (2 mois) Date | date |
| vaccinations | Injection 3 (3 mois) Date | date |
| vaccinations | 1er Rappel (18 mois) Date | date |
| vaccinations | **DTpolio et Coqueluche (INFANRIX TETRA/TETRAVAC)** | enum('Non renseigné','Oui','Non') |
| vaccinations | 5-6 ans Date | date |
| vaccinations | 16-18 ans Date | date |
| vaccinations | **DTpolio** | enum('Non renseigné','Oui','Non') |
| vaccinations | Autre Rappel | date |
| vaccinations | Injection 1 (12-18 mois) Date | date |
| vaccinations | Rappel (13-19 mois) Date | date |
| vaccinations | **Varicelle** | enum('Non renseigné','Oui','Non') |
| vaccinations | Date | date |
| vaccinations | **Hépatite B (ENGERIX B10/HB VAC PRO)** | enum('Non renseigné','Oui','Non') |
| vaccinations | Injection 1 (1 mois) Date | date |
| vaccinations | Injection 2 (2 mois) Date | date |
| vaccinations | Injection 3 (3 mois) Date | date |
| vaccinations | 1er Rappel (18 mois) Date | date |
| vaccinations | **Hépatite A (HAVRIX)** | enum('Non renseigné','Oui','Non') |
| vaccinations | Date | date |
| vaccinations | **Grippe** | enum('Non renseigné','Oui','Non') |
| vaccinations | Injections annuelles (début hiver) | enum('Non renseigné','Oui','Non') |
| vaccinations | vaccin anti-grippe |  |
| vaccinations | **Méningocoque** | enum('Non renseigné','Oui','Non') |
| vaccinations | Date | date |
| vaccinations | **Synagis (injection mensuelle d'octobre à février inclus)** | enum('Non renseigné','Oui','Non') |
| vaccinations | 1ère année | set('Octobre','Novembre','Décembre','Janvier','Février') |
| vaccinations | 2ème année | set('Octobre','Novembre','Décembre','Janvier','Février') |
| vaccinations | **Rotavirus (ROTARIX)** | enum('Non renseigné','Oui','Non') |
| vaccinations | Injection 1 Date | date |
| vaccinations | Injection 2 Date | date |
| vaccinations | **Autre(s) vaccin(s)** | longtext |
| vaccinations | **Pneumocoque (PREVENAR)** | enum('Non renseigné','Oui','Non') |
| vaccinations | Injection 1 (1 mois) Date | date |
| vaccinations | Injection 2 (2 mois) Date | date |
| vaccinations | Injection 3 (3 mois) Date | date |
| vaccinations | Rappel Date | date |
| vaccinations | **Pneumocoque (PNEUMO 23)** | enum('Non renseigné','Oui','Non') |
| vaccinations | Injection Date | date |
| vaccinations | Rappel Date | date |
| vaccinations | **Rougeole, Oreillons, Rubéole (ROR/PRIORIX)** | enum('Non renseigné','Oui','Non') |
| vaccinations | 1ère injection (1 an) | enum('Non renseigné','Oui','Non') |
| vaccinations | Date de 1ère demi dose | date |
| vaccinations | Date de 2ème demi dose (à + 1 mois) | date |
| vaccinations_grippe | identifiant de vaccinations_grippe | |
| vaccinations_grippe | identifiant du patient | int(50) |
| vaccinations_grippe | Date de vaccination | date |
