# Supplementary material for: A national internet-linked based database for pediatric interstitial lung diseases: the French network
Source: Orphanet J Rare Dis. 2012 Jun 15;7:40. doi: 10.1186/1750-1172-7-40 (PMC3458912; doi:10.1186/1750-1172-7-40)
Supplement: Additional file 5: — S5. Characteristics of interstitial lung disease (ILD) patients with no precise diagnosis. [file 1750-1172-7-40-S5.doc]

**Supplemental file 5: Characteristics of Interstitial lung diseases (ILD) patients with no precise diagnosis**

| **Patient number** | **Sex** | **Current age**  **(years)** | **Age at diagnosis**  **(years)** | **Follow up**  **(years)** | ***SFTPC/ABCA3* sequencing** | **Lung biopsy** |
| --- | --- | --- | --- | --- | --- | --- |
| 1 | Female | 0.4 | 0.0 | MD | No | No |
| 2 | Male | 2.2 | 0.0 | 2.0 | Yes | No |
| 3 | Female | 0.9 | 0.1 | 0.6 | Yes | Yes |
| 4 | Female | 0.5 | 0.1 | 0.1 | No | No |
| 5 | Male | 1.1 | 0.2 | 0.0 | No | No |
| 6 | Male | 10.1 | 0.4 | 5.2 | No | No |
| 7 | Female | 2.6 | 0.6 | MD | Yes | Yes. |
| 8 | Male | 4.2 | 0.7 | 2.9 | Yes | Yes |
| 9 | Female | 1.5 | 0.7 | 0.0 | Yes | No |
| 10 | Male | 0.8 | 0.7 | 0.0 | Yes | No |
| 11 | Male | 2.4 | 0.9 | 1.3 | Yes | Yes |
| 12 | Male | 5.7 | 1.0 | 4.6 | Yes | Yes |
| 13 | Male | 2.0 | 1.2 | 0.1 | Yes | No |
| 14 | Female | 3.3 | 1.9 | 0.0 | Yes | No |
| 15 | Male | 2.9 | 2.0 | 0.0 | No | No |
| 16 | Male | 9.5 | 3.0 | 6.5 | Yes | Yes |
| 17 | Female | 4.6 | 3.1 | 1.4 | Yes | No |
| 18 | Female | 14.4 | 3.5 | 9.9 | No | No |
| 19 | Female | 6.1 | 3.7 | 2.3 | Yes | No |
| 20 | Female | 11.7 | 5.4 | 6.1 | Yes | No |
| 21 | Male | 18.3 | 8.5 | 7.1 | Yes | Yes |
| 22 | Male | 9.9 | 8.7 | 0.5 | No | No |
| 23 | Female | 11.9 | 9.6 | 0.8 | No | No |
| 24 | Female | 12.6 | 11.8 | 0.2 | No | No |
| 25 | Male | 17.5 | 12.6 | 4.0 | No | No |
| 26 | Female | 17.3 | 16.4 | 0.9 | No | No |
| 27 | Male | 0.5 | 0.0 | 0.5 | Yes | No |
| 28 | Male | 1.8 | 0.0 | 0.2 | Yes | No |
| 29 | Male | 0.9 | 0.0 | 0.7 | Yes | No |
| 30 | Male | 1.8 | 0.1 | 0.1 | Yes | No |
| 31 | Male | 2.8 | 0.1 | 2.1 | Yes | Yes |
| 32 | Female | 1.4 | 0.2 | 0.0 | No | No |
| 33 | Female | 1.2 | 0.2 | 1.0 | Yes | Yes |
| 34 | Male | 10.7 | 0.2 | 10.4 | Yes | Yes |
| 35 | Female | 17.0 | 0.3 | 13.2 | No | Yes |
| 36 | Male | 12.3 | 0.3 | 11.9 | Yes | Yes |
| 37 | Female | 6.8 | 0.3 | 6.5 | Yes | Yes |
| 38 | Female | 1.5 | 0.4 | 1.1 | Yes | No |
| 39 | Female | 11.5 | 0.6 | 3.3 | No | No |
| 40 | Male | 6.1 | 0.6 | 5.4 | Yes | No |
| 41 | Female | 4.3 | 0.6 | 0.3 | Yes | No |
| 42 | Male | 15.3 | 0.7 | 12.4 | Yes | Yes |
| 43 | Male | 4.3 | 0.8 | 2.7 | Yes | No |
| 44 | Male | 2.7 | 1.0 | 0.3 | Yes | No |
| 45 | Male | 5.2 | 1.5 | 1.1 | No | Yes |
| 46 | Female | 6.8 | 2.9 | 2.0 | Yes | Yes |
| 47 | Male | 12.6 | 3.8 | 5.4 | Yes | No |
| 48 | Male | 6.9 | 4.3 | 1.8 | Yes | No |
| 49 | Female | 7.5 | 5.1 | 1.3 | Yes | Yes |
| 50 | Female | 17.4 | 10.8 | 6.0 | No | Yes |
| 51 | Female | 17.1 | 12.3 | 4.6 | Yes | Yes |
| 52 | Female | 0.2 | 0.2 | 0.1 | Yes | No |
| 53 | Female | 6.7 | 0.4 | 6.2 | Yes | Yes |
| 54 | Female | 1.1 | 0.0 | 1.1 | Yes | Yes |
| 55 | Female | 3.9 | 1.0 | 2.3 | Yes | Yes |
| 56 | Male | 2.1 | 0.8 | 0.1 | Yes | No |
| **Total**  **N=56** | **Male=28, Female=28**  **Sex ratio =1** | **Median = 4.5**  **(0.2-18.3)** | **Median = 0.7**  **(0.0-16.4)** | **Median = 1.4**  **(0-13.2)** | **Total**  **N=40 (71%)** | **Total**  **N=22 (39%)** |
